# Spatial pinning of globally inert pores in a superhydrophobic hydrogen-bonded organic framework for inverse ethane/ethylene separation

**DOI:** 10.1039/d5sc08507a

**Published:** 2025-12-29

**Authors:** Youlie Cai, Jing-Hong Li, Xiaoyan Xiao, Runzhi Wei, Rui-Biao Lin, Banglin Chen, Junkuo Gao

**Affiliations:** a China-Uzbekistan Joint Laboratory on Advanced Porous Materials, State Key Laboratory of Bio-based Fiber Materials, School of Materials Science and Engineering, Zhejiang Sci-Tech University Hangzhou 310018 China jkgao@zstu.edu.cn; b Key Laboratory of Bioinorganic and Synthetic Chemistry of Ministry of Education, School of Chemistry, IGCME, Sun Yat-Sen University Guangzhou 510275 China linruibiao@mail.sysu.edu.cn; c State Key Laboratory of Silicon and Advanced Semiconductor Materials, School of Materials Science and Engineering, Zhejiang University Hangzhou 310027 China; d Fujian Provincial Key Laboratory of Polymer Materials, College of Chemistry and Materials Science, Fujian Normal University Fuzhou 350007 China banglin.chen@fjnu.edu.cn; e Key Laboratory of the Ministry of Education for Advanced Catalysis Materials, College of Chemistry and Materials Science, Zhejiang Normal University Jinhua 321004 China

## Abstract

Overcoming the intrinsic polarity of hydrogen bonds to construct a C_2_H_6_-affinitive nonpolar pore environment using an entirely pore-oriented π-conjugated core presents a formidable challenge within hydrogen-bonded organic frameworks (HOFs). Herein, we propose a spatial pinning strategy for HOF pore construction. Hydrophobic molecular struts are pre-pinned within the precursor to restrict the conformational freedom of hydrogen-bonding arms, thereby governing framework stereochemistry, suppressing undesired π–π stacking, and generating four-way interconnected cavities between π-conjugated layers. Importantly, multiple interpenetrations shield polar hydrogen bonds, enabling a globally inert framework, as evidenced by an impressive contact angle exceeding 153° and an ultralow water vapor uptake of 0.057 g g^−1^. Gas sorption experiments demonstrate a C_2_H_6_ adsorption capacity of 91.5 cm^3^ g^−1^ and a C_2_H_6_/C_2_H_4_ selectivity of 2.0. Gas-loaded single crystals and theoretical calculations reveal that this globally inert pore environment profoundly enhances van der Waals forces between the host framework and C_2_H_6_, facilitating efficient gas packing. Furthermore, this HOF can produce high-purity C_2_H_4_ (>99.9%) from dynamic breakthrough experiments, with a maximum productivity of 36.0 L kg^−1^. This work introduces a pivotal advancement in the precursor design strategy to precisely modulate secondary interaction mechanisms within porous organic frameworks, offering new horizons for customized pore engineering.

## Abstract 2

By spatially pinning struts, we eliminate π–π stacking and shield the H-bond sites. This design affords a framework with engineered superhydrophobic and inert cavities, leading to a preferential uptake of C_2_H_6_ and superior C_2_H_6_/C_2_H_4_ separation.

## Graphical abstract

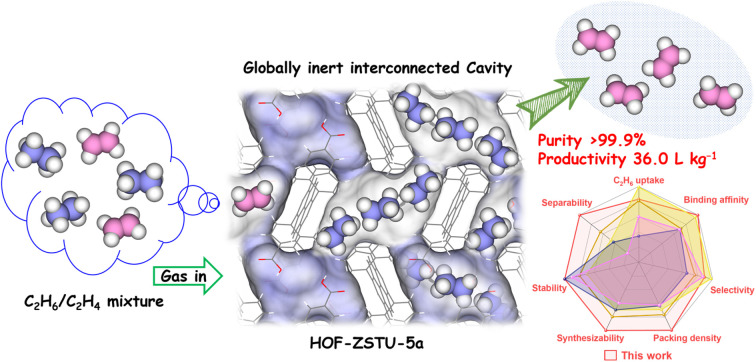

## Introduction

Ethylene (C_2_H_4_) is the cornerstone of the petrochemical industry, serving as a fundamental feedstock for numerous essential chemical products and intermediates.^1^ With global production capacity reaching 228 million tons in 2023 and a projected average annual growth rate (AAGR) exceeding 6%, the demand for high-purity (≥99.9%) C_2_H_4_ is escalating.^2^ Over 90% of C_2_H_4_ is produced *via* steam cracking, yielding a mixture rich in C_2_H_4_ and the byproduct ethane (C_2_H_6_). The stringent requirement for polymer-grade C_2_H_4_ necessitates a highly efficient separation process.^3^ Currently, this separation is overwhelmingly accomplished *via* cryogenic distillation. However, the similar boiling points (∼15 °C difference) and kinetic diameters (∼0.28 Å difference) between C_2_H_6_ and C_2_H_4_ result in distillation accounting for up to 40% of energy consumption, alongside costly cryogenic equipment and operational expenses.^4^ In this context, adsorptive separation using porous materials—particularly the development of C_2_H_6_-selective adsorbents—has emerged as a highly promising alternative strategy due to its potential for simplified operation and energy savings.^5^

Porous materials, including metal–organic frameworks (MOFs) and zeolites, have demonstrated impressive capabilities in this separation.^6–10^ These adsorbents selectively capture one component over another by exploiting subtle differences in molecular size, polarity, or specific interactions such as π-complexation.^11,12^ Nevertheless, a pervasive impediment to practical deployment lies in the inevitable presence of water vapor within the cracking gas.^13^ Despite upstream dehydration processes, residual moisture persists. Such polar molecules readily interact with active sites and compete with the target gases for adsorption sites.^14,15^ For size-exclusion-based adsorbents, water condenses into liquid films or clusters within micropores, impeding target gas access. Moreover, adsorbents exhibiting limited hydrolytic stability face the risk of structural degradation.^16–18^ Overcoming this pervasive challenge thus necessitates a paradigm shift in material design, focusing on the creation of robust adsorbents with engineered water resistance and inherent hydrophobicity.

Over the past decade, hydrogen-bonded organic frameworks (HOFs) have emerged as an innovative class of porous materials for gas separation.^19–24^ Carboxylic acid-based HOFs, distinguished by their highly directional hydrogen-bonded dimer motifs, constitute a key subclass.^25–37^ Precursors containing aromatic rings facilitate the formation of hydrophobic frameworks, offering distinct advantages for achieving C_2_H_6_/C_2_H_4_ separation. Because C_2_H_6_ (44.7 × 10^−25^ cm^3^) is more polarizable than C_2_H_4_ (42.5 × 10^−25^ cm^3^),^38^ nonpolar aromatic surfaces preferentially bind C_2_H_6_ through enhanced dispersion interactions while avoiding the electrostatic interactions associated with the quadrupole moment of C_2_H_4_. Indeed, several reported carboxylic acid-based HOFs leverage this principle, using precursors that direct benzene rings toward the pore surface to improve separation performance.^39–41^ Nevertheless, the inherent polarity of carboxylic acid dimers as hydrogen bond donors/acceptors introduces hydrophilic domains on the pore surface. This hydrophilicity compromises C_2_H_6_ selectivity and curtails the potential of these HOFs as robust hydrophobic adsorbents for engineering purposes. Beyond mitigating surface polarity, achieving optimal C_2_H_6_ recognition demands a precisely tailored pore geometry. Herein lies a critical limitation of HOFs assembled primarily through π–π stacking.^42–45^ The intrinsic cofacial geometry of this interaction hinders the effective projection of the π-electron cloud onto the pore interior, creating a spatially incongruous environment for C_2_H_6_. As a result, the pore walls are deficient in contact points precisely oriented along the molecular axis of C_2_H_6_, which severely curtails the van der Waals forces and thus limits the ultimate binding affinity. A conceptually new strategy to circumvent this limitation is to spatially lock the aromatic cores *via* programmable hydrogen-bonding arms, thereby creating tailored pore chemistry, yet it has never been realized in carboxylic acid-based HOFs.

**Scheme 1:**
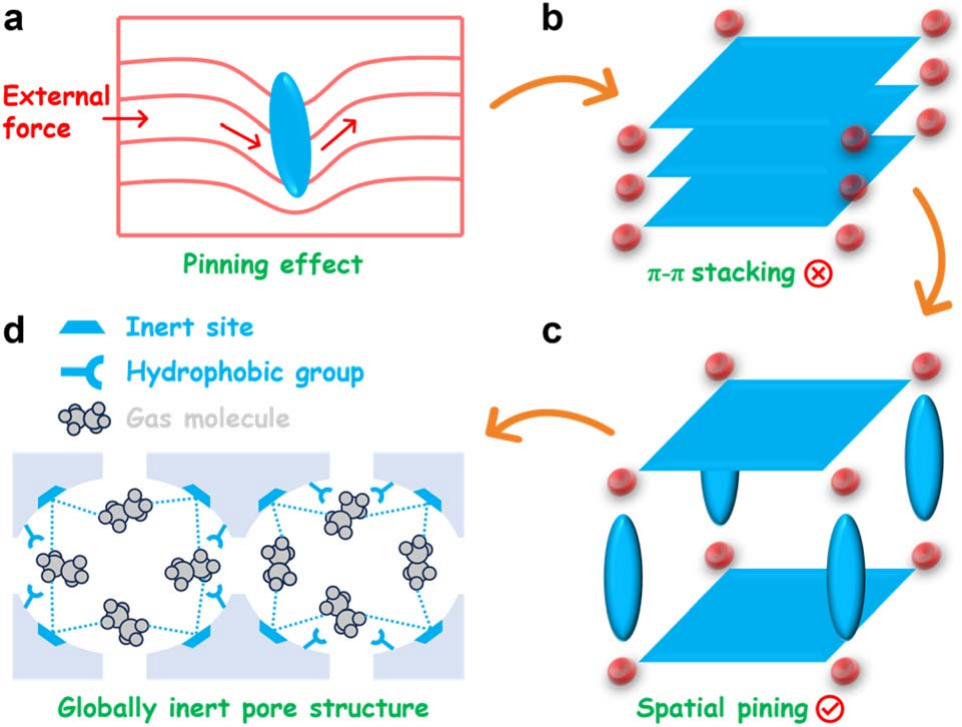
Spatial pinning strategy for an overall hydrophobic HOF for preferential adsorption of C_2_H_6_. (a) Illustration of dislocation pinning. (b) Elimination of π–π stacking by (c) molecular pins separating adjacent layers to generate pore space. (d) Globally inert pore structure produced by this strategy, featuring pyrene cores fully oriented toward the pore surface and methyl sites.

Dislocation pinning is a cornerstone strengthening mechanism in metallurgy, in which microstructural obstacles immobilize dislocations and thereby suppress plastic deformation.^46,47^ By anchoring these line defects, pinning sites impede their glide under stress, necessitating a substantially higher energy input for their movement. This powerful concept of frustrating periodic arrangements *via* engineered obstacles can be translated into molecular design. Specifically, introducing localized steric hindrances within molecular precursors can act as ''molecular pins'' to disrupt periodic π–π stacking, enabling rational control over the resulting supramolecular configuration (Scheme 1).

Inspired by this concept, we present a spatial pinning strategy for HOFs to force the rotation of hydrogen-bonding arms by pinning molecular struts in the precursor. Herein, we report an exceptional inert HOF, designated HOF-ZSTU-5 (ZSTU represents Zhejiang Sci-Tech University). The deliberate installation of methyl groups imparts pronounced steric repulsion between the hydrogen-bonding arms and the aromatic core, forming a stereoscopic precursor conformation. Consequently, the assembled HOF eliminates π–π stacking and establishes a distinctive four-way interconnected cavity within the interlayer voids. The pyrene core is fully oriented toward the pore walls, significantly enhancing the inertness of the framework. Notably, the carboxylic acid hydrogen-bonded dimer is embedded within the framework rather than exposed at the pore surface, realizing global inertness in carboxylic acid-based HOFs, as evidenced by a water contact angle of up to 153° and a saturated water adsorption capacity of only 0.057 g g^−1^ at room temperature. Gas sorption isotherms reveal that the inert environment enables high C_2_H_6_ adsorption capacity (91.5 cm^3^ g^−1^) and packing density (0.349 kg L^−1^), achieving a C_2_H_6_/C_2_H_4_ selectivity of 2.0. Gas-loaded single crystals and theoretical calculations reveal that the optimal binding configuration of C_2_H_6_ within the cavities yields a high adsorption enthalpy while minimizing van der Waals forces with C_2_H_4_. Dynamic breakthrough tests further validate that HOF-ZSTU-5 can achieve high-purity C_2_H_4_ (>99.9%) in one step from mixed gases with varying flow rates and ratios, with a productivity of up to 36.0 L kg^−1^. Remarkably, this high-performance separation is robust against humidity, highlighting the potential of HOF-ZSTU-5 for efficient C_2_H_4_ purification.

## Results and discussion

### Synthesis, structure, and characterization

**Fig. 1:**
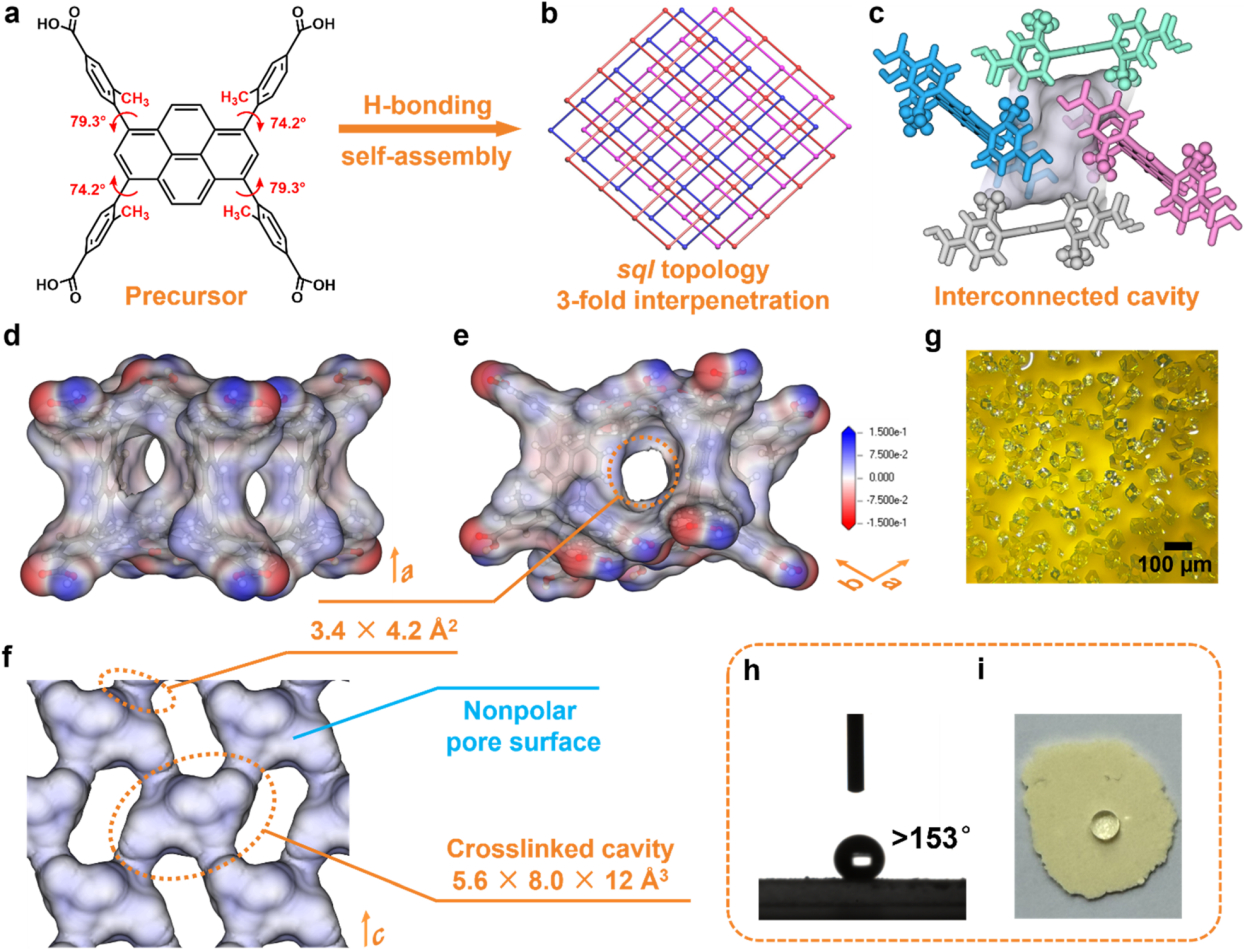
Structure and properties of HOF-ZSTU-5. (a) Conformation of the TBAPy-3-CH_3_ precursor, showing two dihedral angles between the carboxyphenyl and pyrene core; (b) the *sql* topology with 3-fold interpenetration; (c) the four-way interconnected cavities established by interlayer interpenetration; (d)–(f) electrostatic potential mapped onto the pore surface of HOF-ZSTU-5, with pore dimensions indicated. Blue and red colors represent positive and negative potentials, respectively; (g) an optical micrograph of a typical as-synthesized crystal; (h) water contact angle measurement and contact angle of HOF-ZSTU-5; and (i) snapshot of a water droplet on the sample surface.

The light yellow-green rhombic crystals of HOF-ZSTU-5 were obtained by diffusing *n*-hexane or cyclohexane into a methanol (MeOH) solution of 4,4′,4″,4‴-(pyrene-1,3,6,8-tetrayl)tetrakis(3-methylbenzoic acid) (TBAPy-3-CH_3_) at room temperature (Fig. 1a). Single crystal X-ray diffraction (SCXRD) analysis revealed that HOF-ZSTU-5 crystallizes in the orthorhombic space group *Pbca* (Table S1). The asymmetric unit of HOF-ZSTU-5 contains one half of a TBAPy-3-CH_3_ molecule, which is symmetric about the para-axis of the carboxyphenyl arm. Each precursor forms four hydrogen-bonding units, consisting of carboxyl⋯carboxyl hydrogen-bonded dimers with O⋯O distances of 2.651 and 2.725 Å, which extend into a two-dimensional (2D) hydrogen-bonded network (Fig. S1). In the TBAPy-3-CH_3_ precursor, strong steric hindrance arises between the pyrene core and the carboxyphenyl group due to methyl pinning, resulting in high dihedral angles of 74.2 and 79.3° (Fig. S2), which eliminates π–π stacking induced by the proximity of π-conjugated planes. By comparison, the dihedral angle in the parent HOF-101 (also known as PFC-1)^48,49^ precursor is only 37.9°. This highly twisted steric configuration of TBAPy-3-CH_3_ enhances the stability of the framework through the formation of C–H⋯O hydrogen bonds and multiple van der Waals interactions (Fig. S3). Each hydrogen-bonded layer of HOF-ZSTU-5 entangles with two other layers at a planar angle of 48.4°, forming a 3-fold interpenetrated *sql* framework (Fig. 1b and S1). Within this three-dimensional (3D) framework, an interconnected cavity with dimensions of 5.6 × 8.0 × 12 Å^3^ is defined between the interpenetrating layers, which is surrounded by four TBAPy-3-CH_3_ from different layers (Fig. 1c). Among these, the pyrene cores of one pair of parallel TBAPy-3-CH_3_ are oriented toward the cavity, lining its surface with an inert environment. The other two TBAPy-3-CH_3_ delimit four pore windows of 3.4 × 4.2 Å^2^ that connect the adjacent cavities (Fig. 1d, e and S4). Each pore window is gated by two methyl groups, which serve dual functions as spatial confinement agents and binding sites. Importantly, the interlayer interpenetration strategically shields the polar carboxylic acid dimers within the framework and away from the pore region. The resulting inert pore environment, which is confirmed by electrostatic potential mapping of the cavities, significantly enhances the binding affinity for nonpolar gas molecules (Fig. 1f, S5 and S6).

The phase purity of HOF-ZSTU-5 was verified by powder X-ray diffraction (PXRD) analysis, with the experimental patterns for both the as-synthesized and activated samples showing excellent agreement with the pattern simulated from single crystal diffraction data (Fig. S7). The high structural order is visualized by optical microscopy (OM) and scanning electron microscopy (SEM), which show uniformly shaped crystals whose morphology is predicted using the Bravais–Friedel–Donnay–Harker (BFDH) algorithm for thermodynamically stable growth (Fig. 1g, S8 and S9). Thermogravimetric analysis (TGA) of the activated HOF-ZSTU-5 (denoted HOF-ZSTU-5a) revealed a thermal decomposition onset above 340 °C under a N_2_ atmosphere (Fig. S10). This superior thermal stability, which surpasses that of the parent framework, can be attributed to the robust interpenetrated architecture stabilized by the pinned, rigid conformation.

### Contact angle and water sorption experiments

**Fig. 2:**
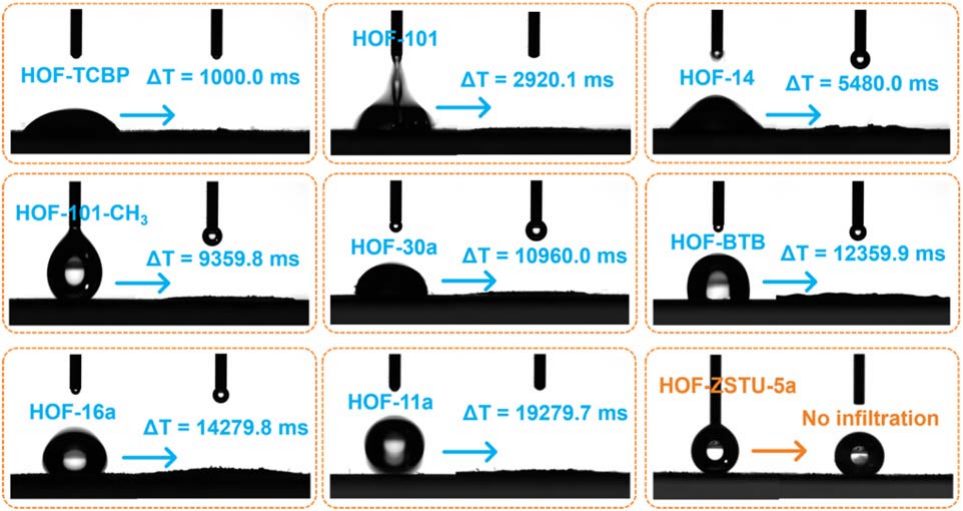
Snapshots of contact angle measurements for a series of carboxylic acid-based HOFs, showing the time required for water droplets to fully infiltrate from the moment of contact with the HOF surface. Among them, HOF-ZSTU-5a exhibits no infiltration due to its superhydrophobicity.

Water contact angle tests were conducted on a series of carboxylic acid-based HOFs to assess their apparent hydrophobicity (Fig. 2 and S11–S14). The infiltration of water droplets was observed on the surfaces of the other eight activated carboxylic acid-based HOFs, although the time required for complete infiltration varied among these HOFs. In contrast, only gravitational deformation without discernible spreading was observed on the surface of HOF-ZSTU-5a. Five consecutive contact angle measurements confirmed that HOF-ZSTU-5a exhibited a water contact angle of at least 153°, demonstrating superhydrophobicity (Fig. S15). This finding confirms our hypothesis that the spatial pinning strategy, which structurally buries the polar hydrogen-bonding networks, is highly effective in minimizing surface wettability. The superhydrophobicity of HOF-ZSTU-5a is remarkable, exceeding not only typical carboxylic acid-based HOFs but also prominent non-carboxylic examples such as HOF-FJU-1a (141°, Fig. S16),^50^MTHOF-1 (121.4°),^51^ and HOF-NKU-1 (87.8°),^42^ as well as other classes of porous materials (Table S4).

**Fig. 3:**
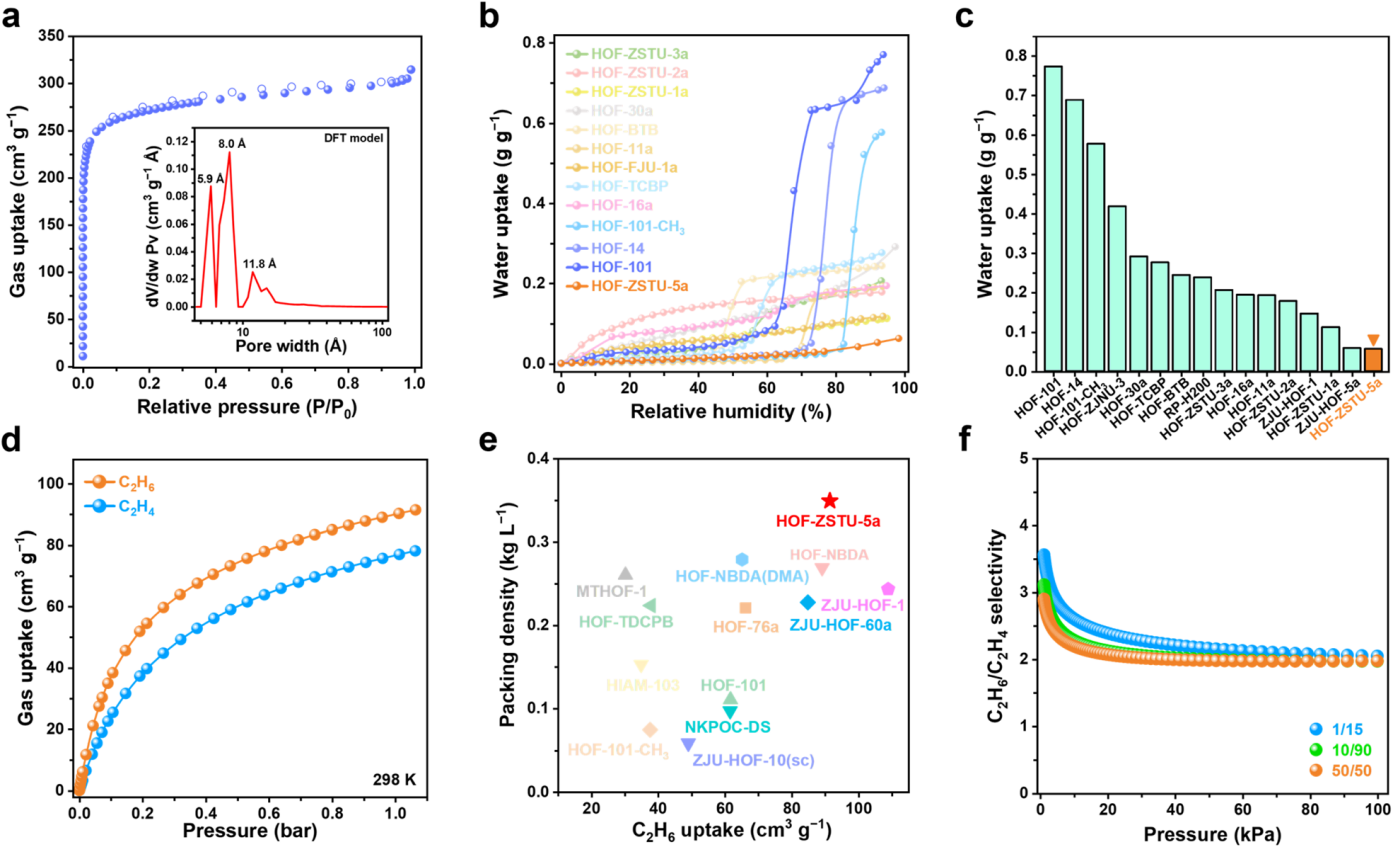
(a) The 77 K N_2_ sorption isotherm of HOF-ZSTU-5a and its pore size distribution. (b) Water uptake isotherms of a series of carboxylic acid-based HOFs at 298 K. (c) Water uptake of carboxylic acid-based HOFs at 298 K and near-saturation humidity. (d) Single-component gas sorption isotherms of HOF-ZSTU-5a at 298 K. (e) The uptake and packing density of C_2_H_6_ for C_2_H_6_-selective HOFs were comprehensively compared. (f) C_2_H_6_/C_2_H_4_ selectivity of HOF-ZSTU-5a at 298 K.

To probe the intrinsic hydrophobicity of the pore interior, water vapor sorption isotherms of carboxylic acid-based HOFs were measured at 298 K (Fig. 3b and S17–S21). The water uptake of HOF-ZSTU-5a at near saturation is only 0.057 g g^−1^, which is 1/13 and 1/10 of that of HOF-101 (0.773 g g^−1^) and HOF-101-CH_3_ (0.577 g g^−1^),^52^ respectively, and comparable to that of ZJU-HOF-5a (0.059 g g^−1^).^53^ Even when compared to other carboxylic acid-based HOFs and C_2_H_6_-selective adsorbents, it remains at a low value (Fig. 3c and Table S5). Combined with its superhydrophobic surface, HOF-ZSTU-5a represents a rare example of an overall hydrophobic material, similar to but superior to SMS-POC-1.^54^ This is a rare attribute for hydrogen-bonded architectures, implying that the spatial pinning strategy effectively prevents water clustering within the pores, thereby paving the way for robust separation performance in humid environments.

### Porosity and C_2_ gas uptake measurements

The potential porosity of HOF-ZSTU-5a was calculated to be 33.4% using the PLATON solvent model.^55^ The pore volume calculated from the crystal structure is 0.358 cm^3^ g^−1^. The experimental permanent porosity was determined from the N_2_ sorption isotherm at 77 K (Fig. 3a and S22). HOF-ZSTU-5a exhibits a typical type I microporous isotherm, with a total N_2_ uptake of 314 cm^3^ g^−1^, corresponding to a Brunauer–Emmett–Teller (BET) surface area of 1060 cm^2^ g^−1^ (Fig. S23). The DFT pore size model revealed that the apertures of HOF-ZSTU-5a are concentrated at 5.9, 8.0, and 11.8 Å, which fits well with the dimensions of the interconnected cavity. This relatively shrunken nonpolar pore structure is expected to enhance the binding affinity to the target gas and facilitate dense packing.

Single-component sorption isotherms were used to evaluate the actual effect of different pore structures on gas sorption capacity. At 298 K and 1 bar, HOF-ZSTU-5a exhibits a remarkably high C_2_H_6_ uptake of 91.5 cm^3^ g^−1^ and a C_2_H_4_ uptake of 78.2 cm^3^ g^−1^ (Fig. 3d). This performance significantly surpasses that of the parent HOF-101 (61.7 and 52.0 cm^3^ g^−1^ of C_2_H_6_ and C_2_H_4_ respectively) and HOF-101-CH_3_ (37.6 and 25.9 cm^3^ g^−1^). The superior capacity of HOF-ZSTU-5a highlights the advantage of the spatial pinning strategy: the resulting tighter pore confinement enhances van der Waals contacts, whereas the larger, open pores of the reference frameworks fail to interact strongly with the gas molecules. At 273 K, the uptake of HOF-ZSTU-5a for C_2_H_6_ and C_2_H_4_ further increases to 110.0 and 98.9 cm^3^ g^−1^ (Fig. S24). Notably, the C_2_H_6_ capacity of HOF-ZSTU-5a at 298 K ranks among the top tier of reported HOFs, second only to ZJU-HOF-1 (109 cm^3^ g^−1^),^39^ and outperforms a range of prominent HOFs including HOF-NBDA (89.2 cm^3^ g^−1^),^41^HOF-76a (66.1 cm^3^ g^−1^),^56^ and NKPOC-DS (61.6 cm^3^ g^−1^),^57^ as well as benchmark MOFs such as Fe_2_(O_2_)(dobdc) (74.3 cm^3^ g^−1^),^9^Cu(Qc)_2_ (41.5 cm^3^ g^−1^),^58^ and PCP-IPA (41.5 cm^3^ g^−1^).^59^ To quantify the filling efficiency, the C_2_H_6_ packing density in HOF-ZSTU-5a was determined to be 0.349 kg L^−1^, which is 286 times that of gaseous C_2_H_6_ under identical conditions (1.22 × 10^−3^ kg L^−1^), underscoring its exceptional pore-filling efficiency (Fig. 3e).

The isosteric heat of adsorption (*Q*_st_) was derived from the isotherms at 298 and 273 K using the Virial equation (Fig. S25–S28). At zero coverage, HOF-ZSTU-5a exhibits a *Q*_st_ of 32.9 kJ mol^−1^. This value falls within the ideal range for physisorption, indicating that the nonpolar pore walls provide adequate van der Waals attraction for efficient capture, while avoiding the excessive energy penalties associated with difficult regeneration. Dynamically, the *Q*_st_ curve initially declines as preferred binding sites are saturated, but subsequently rises at higher loadings. This inflection signifies the onset of strong cooperative guest–guest interactions, providing thermodynamic evidence for the dense packing mechanism within the confined pores. Ultimately, by achieving a synergy of high capacity, exceptional packing density, and energy-efficient binding (*Q*_st_), HOF-ZSTU-5a sets a new performance standard among C_2_H_6_-selective porous materials (Fig. S29 and Tables S6, S7).

To evaluate the separation efficiency for binary mixtures, selectivities were calculated based on Ideal Adsorbed Solution Theory (IAST) (Fig. S30–S32). HOF-ZSTU-5a maintained the highest selectivity across the entire pressure range, with a selectivity of 2.0 at 1 bar, representing a marked improvement over HOF-101-CH_3_ (1.45) and HOF-101 (1.3) (Fig. 3f). Even for a typical low-concentration cracked gas mixture (v/v = 1/15, C_2_H_6_/C_2_H_4_), HOF-ZSTU-5a exhibits a high selectivity of 2.05 (Fig. S33). Furthermore, the separation potential (Δ*q*) was used as a combined indicator to weigh the trade-off between the capacity and selectivity of adsorbents (Fig. S34 and S35). Remarkably, the Δ*q* value of HOF-ZSTU-5a (1.22 mmol g^−1^) was superior to that of HOF-101-CH_3_ (0.25 mmol g^−1^), HOF-101 (0.31 mmol g^−1^), MTHOF-1 (0.51 mmol g^−1^), and HOF-76a (0.81 mmol g^−1^), as well as many C_2_H_6_-selective MOFs (Table S8). These results confirm that the spatial pinning configuration successfully creates a stereochemical environment that is optimally tuned for C_2_H_6_, simultaneously maximizing uptake and selectivity to achieve superior separation performance.

### GCMC simulation study

The binding behavior between guest molecules and the host framework was investigated using grand canonical Monte Carlo (GCMC) simulations. At 298 K and 100 kPa, the simulations for HOF-ZSTU-5a identified three high-density regions for C_2_H_6_ at the cavity wall (site I) and center (site II), whereas C_2_H_4_ presented only two symmetrical distributions, corresponding to a saturation loading of three C_2_H_6_ or two C_2_H_4_ molecules per unit cavity (Fig. S36 and S37). These guest molecules exhibit anisotropic distribution, favoring the longer pathway between opposing cavity openings rather than uniform distribution across all four openings. This directional preference arises from favorable molecular diffusion kinetics, possibly driven by energy barriers or steric constraints. In contrast, both homologous HOFs based on π–π stacking exhibit a marked underfilling of the guests (Fig. S38–S41), implying that larger pore sizes and heterogeneous pore polarity is detrimental to the formation of affinity sites. Due to the presence of *ortho-*methyl groups, the guest approaches the pyrene sidewalls of the HOF-101-CH_3_ pore in a co-planar orientation, whereas in HOF-101 without methyl group sites, it is in a perpendicular orientation. This suggests that guest molecules are adaptively captured in the framework according to the pore structural properties. Overall, the combination of a homogeneous, globally inert cavity surface and an optimized diffusive pore topology represents a key advantage of HOF-ZSTU-5a for maximizing pore utilization.

### Gas-loaded single crystal configuration

**Fig. 4:**
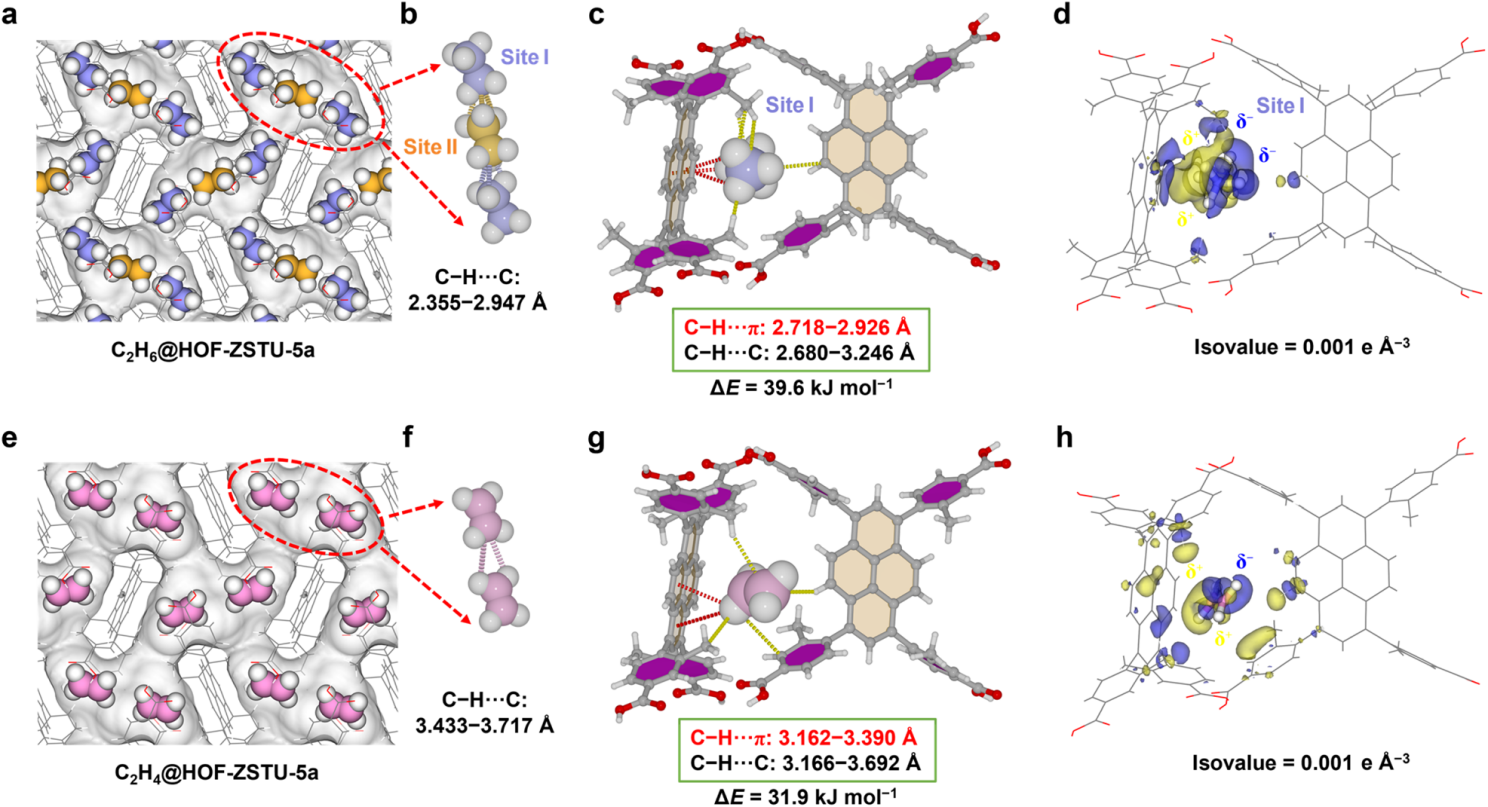
HOF-ZSTU-5a crystal structure of *in situ* loaded (a) C_2_H_6_ and (e) C_2_H_4_ at 150 K. The guest–guest interactions between (b) three C_2_H_6_ and (f) two C_2_H_4_ molecules in a single cavity. The host–guest interaction forms between the framework and (c) site I C_2_H_6_, and (g) C_2_H_4_ in the gas-loaded single crystal. Differential electron density map between the framework and (d) site I C_2_H_6_, and (h) C_2_H_4_, where the blue region denotes charge aggregation and the yellow region denotes charge dissipation.

The crystal structures of HOF-ZSTU-5a loaded C_2_H_6_ or C_2_H_4_ were determined by *in situ* SCXRD (Fig. S42 and Tables S2, S3). The gas-loaded single crystals (C_2_H_4_@HOF-ZSTU-5a at 273 K and C_2_H_6_@HOF-ZSTU-5a at 273 K) reveal two symmetrically distributed molecules of C_2_H_6_ or C_2_H_4_ per cavity (Fig. S43). The gas-loaded conformation of C_2_H_4_ is consistent with theoretical simulations, while only an incomplete symmetric distribution form of C_2_H_6_ in the cavity is observed. The lack of central C_2_H_6_ implies unresolved kinetic or thermodynamic factors, which motivates us to investigate the interactions and adsorption kinetics of guests in the pores at low temperatures. Two crystallographically independent C_2_H_6_ molecules were identified using a C_2_H_6_-loaded HOF-ZSTU-5a crystal at 150 K (C_2_H_6_@HOF-ZSTU-5a at 150 K), forming guest–guest interactions with three short-range orientational forces at distances of 2.355–2.947 Å for dense packing in the pore (Fig. 4a and b). The two C_2_H_6_ occupy distinct adsorption sites at the cavity edge and center, consistent with theoretical calculations (Fig. S44 and S45). The C_2_H_6_ at site I was close to the cavity wall consisting of two TBAPy-3-CH_3_, forming three moderately strong C–H⋯π hydrogen bonds (2.718–2.926 Å) with one pyrene core, and a C–H⋯C van der Waals dispersion force (3.214 Å) with the other precursor. In addition, it is subject to four C–H⋯C induced dipole interactions from two *meta-*methyl groups, with lengths of 2.680–3.246 Å (Fig. 4c). Such spatial confinement arises from the enthalpic trap that C_2_H_6_ needs to overcome when passing through cavity apertures narrower than their kinetic diameter. For the C_2_H_6_ at site II (Fig. S46), only two sets of C–H⋯C van der Waals interactions (3.479 and 3.494 Å) with the two symmetric precursors were observed, indicating weakened host–guest interaction in the central region. This weak binding tends to be hidden by molecular thermal motions with increasing temperature, explaining the inability of C_2_H_6_ from site II to be resolved at ambient temperature. The C_2_H_4_-loaded single crystals (C_2_H_4_@HOF-ZSTU-5a at 150 K) show only one crystallographically symmetric distribution of C_2_H_4_ in the cavity (Fig. 4e). Owing to its larger quadrupole moment (Table S9), C_2_H_4_ preferentially resides in regions of enhanced polarity. As a result, the two symmetric C_2_H_4_ molecules are positioned closer to the cavity center, exhibiting guest–guest interactions with lengths of 3.433–3.717 Å (Fig. 4f), which are longer than the interactions between C_2_H_6_ molecules. The C_2_H_4_ forms two C–H⋯π hydrogen bonds (3.162 and 3.390 Å) with the pyrene core and two C–H⋯C van der Waals dispersion interactions (3.318 and 3.692 Å) with aromatic rings, and two C–H⋯C interactions (3.166 and 3.443 Å) with two *meta-*methyl groups (Fig. 4g). The weak host–guest interactions indicate a low compatibility of C_2_H_4_ with the inert pore environment. A comparison of the four gas-loaded single crystals revealed a moderate lattice expansion in C_2_H_6_-loaded crystals at ambient temperature (+0.9%) compared to those at low temperature, whereas C_2_H_4_-loaded crystals exhibited only a slight expansion (+0.2%). The differential expansion phenomenon is related to the occupancy of the guest molecules and the host–guest interactions, which suggests a thermodynamically driven framework adaptive effect. The above results show a dual distribution of C_2_H_4_ in the cavity without additional adsorption sites. This binding behavior is attributed to the incompatibility between the π-electrons from the C_2_H_4_ C=C bond and the nonpolar pore environment, blocking C_2_H_4_ from occupying the “sweet spot” of site binding and thereby reducing uptake.

The host–guest energies of the optimized gas-loaded single crystals were calculated using first-principles dispersion-corrected density functional theory (DFT-D). The static binding energies of C_2_H_6_ at the edge and center of the cavity are 39.6 and 29.7 kJ mol^−1^. The calculated weighted average of 36.3 kJ mol^−1^ is notably higher than that of C_2_H_4_ (31.9 kJ mol^−1^). These values align well with the *Q*_st_ at zero coverage, confirming that guest molecules exhibit enhanced binding affinity near the pore walls. The preferential binding sites for C_2_H_6_/C_2_H_4_ in the other two HOFs are located on the pyrene sidewalls, with binding energies of 30.5/24.2 kJ mol^−1^ to HOF-101 and 20.4/18.4 kJ mol^−1^ to HOF-101-CH_3_ (Fig. S47 and S48). This comparative result indicates that while the pores of HOF-101 permit effective interaction with the pyrene surface, the steric bulk of the *ortho*-methyl groups in HOF-101-CH_3_ displaces the guest molecules away from the π-surfaces. This increased separation distance drastically diminishes the dominant dispersion forces, which cannot be sufficiently compensated for by the methyl–guest interactions, resulting in the lowest binding affinity.

The variations of the charge distributions of the guest and the framework were further calculated. For the gas-loaded single crystal of HOF-ZSTU-5a at 150 K, C_2_H_6_ exhibits a stronger charge shift than C_2_H_4_ at the same isovalue (Fig. 4d). In particular, the methyl group electron cloud of C_2_H_6_ at site I shifts toward the pyrene core and the *meta-*methyl group. This rearrangement facilitates a C^*δ*−^_(ethane)_–H^*δ*+^_(ethane)_⋯C^*δ*−^_(framework)_ dispersion interaction. Concurrently, the carboxyphenyl groups shift electron density toward the C_2_H_6_, reinforcing the C^*δ*−^_(framework)_–H^*δ*+^_(framework)_⋯C^*δ*−^_(ethane)_ interaction. The charge shift of the central C_2_H_6_ is relatively weak and relevant to its lower binding energy (Fig. S46). A noticeable electron rearrangement also occurs as the π-electron of C_2_H_4_ shifts toward the aromatic rings and pyrene core (Fig. 4h), although to a lesser extent than in C_2_H_6_. We also describe the host–guest contact distances by mapping the *d*_norm_ values onto the Hirshfeld surface of the guest molecules to quantify these interactions.^60^ The contact distance becomes shorter than the sum of the van der Waals radii when *d*_norm_ is negative, which is shown in red, indicating a strong intermolecular interaction. In HOF-ZSTU-5a, the strong contact of C_2_H_6_ exists not only with the pyrene core and *meta-*methyl group, but also between the C_2_H_6_ molecules, with the minimum *d*_norm_ values of −0.5717 and −0.4870 for site I and site II (Fig. S49). A much weaker contact for C_2_H_4_ is evident, with a minimum *d*_norm_ value of 0.2095 (Fig. S50). The types of interactions between the guest molecules and the framework were analyzed through 2D fingerprint plots. The short-range and concentrated distances of C_2_H_6_ (0.6–2.3 Å) and the framework (0.8–2.9 Å) on the Hirshfeld surface are composed of 79.7% H⋯H and 20.3% H⋯C, embodied in C–H⋯π and C–H⋯C interactions (Fig. S49). The distance between C_2_H_4_ and the framework on the Hirshfeld surface is longer and more dispersed, with 77.9% H⋯H and 19.5% H⋯C, and the remaining 2.6% C⋯C reflecting repulsion between the C^*δ*−^_(ethylene)_ and C^*δ*−^_(framework)_ (Fig. S50). The above discussion strongly confirms that a globally inert pore environment not only provides multiple interactions for C_2_H_6_, but also creates suitable steric confinement, which is essential for achieving preferential adsorption of C_2_H_6_ over C_2_H_4_.

### Dynamic breakthrough experiments

**Fig. 5:**
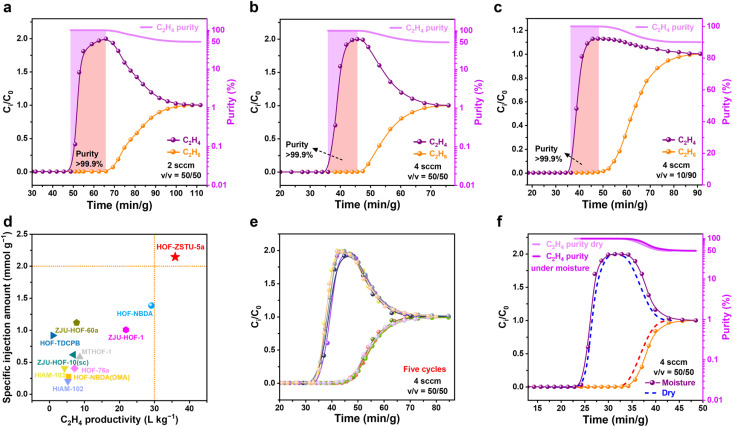
The breakthrough tests of HOF-ZSTU-5a were performed at 298 K and 1 bar for equimolar C_2_H_6_/C_2_H_4_ mixtures with flow rates of (a) 2 mL min^−1^ and (b) 4 mL min^−1^ and (c) C_2_H_6_/C_2_H_4_ of 10/90 (v/v) at a flow rate of 4 mL min^−1^. (d) Comparison of the specific injection amount and C_2_H_4_ productivity of C_2_H_6_-selective HOFs. (e) Five consecutive breakthrough tests at 298 K. (f) Breakthrough test at near-saturation humidity and compared to dry conditions.

Considering the selective compatibility of the interconnected cavity with C_2_H_6_, the dynamic separation performance of HOF-ZSTU-5a for C_2_H_6_/C_2_H_4_ mixed gases under different conditions was evaluated from a practical application perspective. First, dynamic breakthrough experiments were conducted at 298 K using equimolar C_2_H_6_/C_2_H_4_ mixtures at low total flow rates of 1 and 2 mL min^−1^ (Fig. 5a and S51). As the binary gas mixture passes through the fixed bed packed with the activated adsorbent, polymer-grade C_2_H_4_ (99.9%) is initially detected at the outlet. Once the adsorbent reaches dynamic saturation, the C_2_H_4_ outflow concentration begins to decrease, at which point C_2_H_6_ breaks through from the fixed bed. After calibration by adsorbent mass, the retention times of C_2_H_4_ at the two flow rates were 27.3 and 17.1 min g^−1^. Breakthrough curve calculations for HOF-ZSTU-5a yield C_2_H_4_ productivities of 12.2 and 13.3 L kg^−1^ for purities >99.9% in one cycle. The good separation performance was maintained even when half the adsorbent mass is used (Fig. S51). To optimize the process space-time yield and suppress mass-transfer zone tailing, the flow rate was elevated to 4 mL min^−1^ under the same conditions (Fig. 5b). The C_2_H_4_ yield per cycle reached 17.5 L kg^−1^, comparable to that of Fe_2_(O_2_)(dobdc) (17.7 L kg^−1^), while the total dynamic separation time was reduced to approximately 60 min g^−1^, optimizing the balance between production efficiency and separation performance. Moreover, using a 10/90 (v/v) C_2_H_6_/C_2_H_4_ mixture to simulate industrial feed conditions yielded a C_2_H_4_ productivity of 36.0 L kg^−1^ (Fig. 5c), which is more than twice that of an equimolar mixture and comparable to that of SNNU-181-Mn_3+6_ (39.6 L kg^−1^) as a benchmark material.^61^ The specific injection amount of the gas mixture was used as a normalized indicator of the breakthrough flow rate and runtime to evaluate various HOFs and MOFs.^62^ In equimolar mixtures, HOF-ZSTU-5a showed the optimal balance among HOFs with the highest specific injection amount (2.14 mmol g^−1^), which was 1.6 times higher than the second-ranked HOF-NBDA and NKPOC-DS (1.38 mmol g^−1^) (Fig. 5d and Table S6) and also higher than MOF materials such as Fe_2_(O_2_)(dobdc) (1.99 mmol g^−1^), Zn-atz-ipa (0.5 mmol g^−1^),^63^ and PCP-IPA (1.41 mmol g^−1^), second only to TYUT-17 (3.13 mmol g^−1^) (Fig. S52 and Table S10).^64^ We also performed breakthrough tests on HOF-101 and HOF-101-CH_3_ at room temperature (Fig. S53 and S54). For 10/90 (v/v) mixtures, their penetration time intervals were 2.5 and 3.6 min g^−1^. Despite the short intervals, C_2_H_4_ productivities of 2.8 and 1.14 L kg^−1^ (>99.9%) were also obtained, respectively. However, C_2_H_6_ penetrated quickly out of the beds in equimolar mixtures, with C_2_H_4_ purities reaching only 96.0%. These breakthrough results verified that HOF-ZSTU-5a with a unique pore structure was able to intercept C_2_H_6_ for the one-step efficient separation and purification of C_2_H_4_.

### Stability and comprehensive performance

**Fig. 6:**
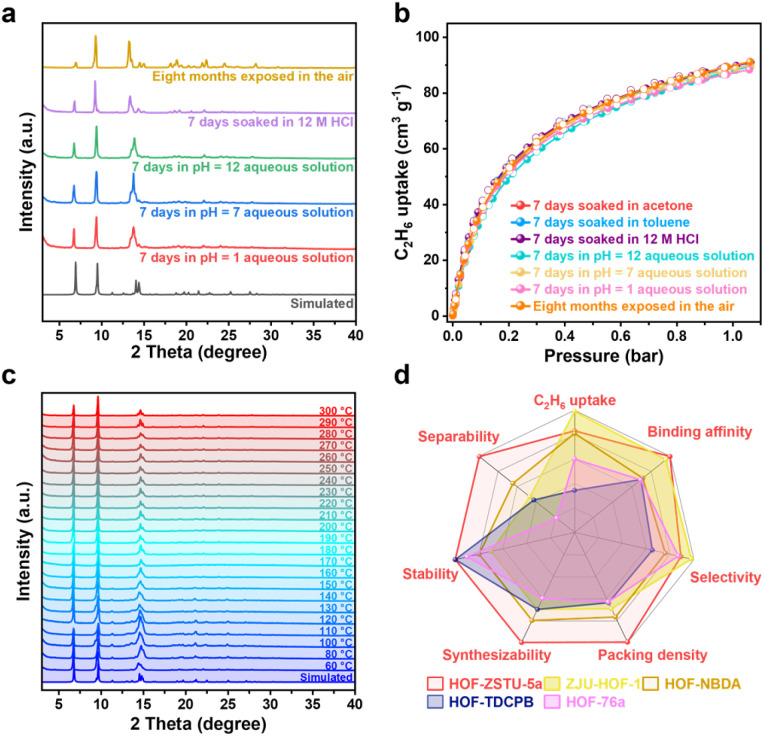
(a) PXRD and (b) C_2_H_6_ sorption isotherms of HOF-ZSTU-5a after undergoing several different environmental stability experiments. (c) Variable-temperature PXRD curve of HOF-ZSTU-5a. (d) A comprehensive comparison of C_2_H_6_ uptake, binding affinity, selectivity, packing density, separability, stability, and synthesizability of HOF-ZSTU-5a and other high-performance carboxylic acid-based HOFs.

Given the harsh nature of industrial environments, the robustness and practical feasibility of HOF-ZSTU-5a were rigorously evaluated. The material exhibited exceptional cycling stability, maintaining its uptake capacity over five static sorption–desorption cycles at 298 K (Fig. S55). This stability translates to dynamic conditions, as evidenced by five consecutive breakthrough runs without performance loss (Fig. 5e). Notably, complete regeneration was achieved under mild conditions (argon purge at 50 °C), a temperature significantly lower than typical thermal regeneration requirements. This regeneration temperature is well within the typical range of 40–100 °C used for industrial processes like pressure swing adsorption (PSA), positioning HOF-ZSTU-5a within the low-energy window for industrial separations. The chemical durability of HOF-ZSTU-5a is particularly striking. The framework retained its crystallinity and porosity after week-long immersion in diverse organic solvents and aqueous solutions ranging from pH 1 to 12. Most impressively, it withstood exposure to 12 M HCl, exhibiting negligible structural degradation (Fig. 6a, b and S56). Thermal stability was also assessed by exposing the samples to air at different temperatures for at least one hour, and the PXRD curves showed that HOF-ZSTU-5a remained highly crystalline up to at least 300 °C (Fig. 6c). Notably, an enhanced breakthrough capacity was observed under near-saturated humidity, which is attributed to a weak synergistic effect between C_2_H_6_ and trace water molecules retained in the pore channels (Fig. 5f and S57).^65^ Beyond performance, the material offers solution processability, a distinct advantage of HOFs. HOF-ZSTU-5a can be easily recrystallized from methanol, and the mother liquor is recyclable, underscoring its potential for sustainable, green manufacturing. Summarized by the radar chart comparing stability, capacity, selectivity, and productivity (Fig. 6d and Table S11), HOF-ZSTU-5a represents a holistic solution for robust and energy-efficient C_2_H_4_ purification.

## Conclusions

In summary, we have engineered a distinct supramolecular conformation through a spatial pinning strategy. By strategically installing methyl groups to act as steric barriers, we successfully suppressed the conventional π–π stacking and assembled a robust HOF with a four-way interconnected pore structure. A multi-interpenetrating hydrogen-bonding network shields the polar carboxylic acid dimers, enabling the construction of a completely inert HOF. This defined pore structure enables a cooperative mechanism for C_2_H_6_/C_2_H_4_ separation: the pyrene core acts as the primary binding site for C_2_H_6_, while the methyl groups create constricted apertures that enhance selectivity through spatial confinement. The mechanism of preferential capture is rigorously validated by gas-loaded single crystals and DFT-D calculations. As a result, this HOF achieves the one-step production of high-purity C_2_H_4_ with a high productivity of 36.0 L kg^−1^ in dynamic breakthrough experiments. Its exceptional hydrophobicity ensures stable separation performance under humid conditions, underscoring industrial relevance. This study overcomes the intrinsic limitations of conventional π–π stacked HOFs through a target-oriented control of supramolecular conformation and further elucidates how the synergistic integration of functional sites and spatial strategies dictates the structure–property relationships in supramolecular frameworks.

## Author contributions

Conceptualization: B. C., R.-B. L., J. G., and Y. C.; methodology and investigation: Y. C. and R. W.; single crystal analysis and refinement: J.-H. L., X. X., and Y. C.; writing—original draft: Y. C.; writing—review & editing: R.-B. L., J. G., and Y. C.; funding acquisition: B. C., R.-B. L., and J. G.

## Conflicts of interest

The authors declare no competing interests.

## Supplementary Material

SC-OLF-D5SC08507A-s001

SC-OLF-D5SC08507A-s002

## Data Availability

All other data supporting the findings of this study are included in the article and the supplementary information (SI) or are available from the lead contact upon reasonable request. CCDC 2425853 (HOF-ZSTU-5a), 2425854 (HOF-ZSTU-5), 2425855 (C_2_H_4_@HOF-ZSTU-5a at 150 K), 2425856 (C_2_H_6_@HOF-ZSTU-5a at 150 K), 2425857 (C_2_H_4_@HOF-ZSTU-5a at 273 K) and 2425858 (C_2_H_6_@HOF-ZSTU-5a at 273 K) contain the supplementary crystallographic data for this paper.^66*a*–*f*^ Supplementary information: data collection and refinement details are listed in Tables S1–S3. See DOI: https://doi.org/10.1039/d5sc08507a.
