# Supplementary material for: Spatial pinning of globally inert pores in a superhydrophobic hydrogen-bonded organic framework for inverse ethane/ethylene separation
Source: Chem Sci. 2025 Dec 29;17(8):4256–67. doi: 10.1039/d5sc08507a (PMC12787314; doi:10.1039/d5sc08507a)
Supplement: SC-017-D5SC08507A-s001 [file SC-017-D5SC08507A-s001.pdf]

Supplementary Information (SI) for Chemical Science.
This journal is © The Royal Society of Chemistry 2025

Supporting Information

Spatial Pinning of Globally Inert Pores in Superhydrophobic Hydrogen-Bonded Organic Framework for Inverse Ethane/Ethylene Separation

Youlie Cai, Jing-Hong Li, Xiaoyan Xiao, Runzhi Wei, Rui-Biao Lin,* Banglin Chen,* and Junkuo Gao*\

Materials and supplies

1,3,6,8-tetrakis(p-benzoic acid)pyrene (TBAPy, CAS: 933047-52-0) was purchased from Zhengzhou Alfa Chemical Co., Ltd., 1,3,6,8-tetra(2-methylbenzoic acid-4-yl)pyrene(TBAPy-2-CH_3, CAS: 2682204-05-1) and 4,4',4'',4'''-(pyrene-1,3,6,8-tetrayl)tetrakis(3-methylbenzoic acid) (TBAPy-3-CH_3, CAS: 2982238-30-0) were purchased from Nanchang Chouhechem Pharmatech Co., Ltd., methanol (MeOH, CAS:67-56-1), cyclohexane (CAS: 110-82-7), n-hexane (CAS: 110-54-3), *N,N*-dimethylformamide (DMF, CAS: 68-12-2), and dimethylacetamide (DMA, CAS: 127-19-5) were purchased from Energy Chemical, propanone (PA, CAS: 67-64-1) and methylbenzene (MB, CAS: 108-88-3) were purchased from Huzhou Shuanglin Chemical Technology Co., Ltd. He (99.999%), N_2 (99.999%), C_2H_4 (99.99%), C_2H_6 (99.99%), mixed gases of C_2H_4/C_2H_6 (50/50, v/v), and C_2H_4/C_2H_6 (90/10, v/v) mixtures were purchased from JinGong Company (China). All chemicals purchased from commercial companies were used directly without further treatment.

1. Characterization

Powder X-ray diffraction (PXRD) patterns (3 to 40°) were recorded on Bruker D8 Advance diffractometer with Cu Kα radiation. Thermogravimetric analysis (TGA) was performed on a NETZSCH TG 209 F1 Thermogravimetric Analyzer at a heating rate of 20 °C min^{-1} from 30 °C to 800 °C under N_2 atmosphere. The scanning electron microscope images were taken with a thermal field emission scanning electron microscope (SEM) manufactured by Carl Zeiss SMT Pte Ltd, model vltra55. The optical images of the crystals were taken using an optical microscope (OM, model CX40M) manufactured by Ningbo Sunyu Instrument Co., Ltd. The water contact angle tests were carried out on an Optical Surface Analyzer model OSA100S manufactured by Ningbo NB Scientific Instruments Co., Ltd. The specific surface areas of the samples were measured with N_2 sorption isotherms by the Brunauer-Emmett-Teller (BET) method at 77 K with a fully automated analyzer model BSD-660M A3M from BeiShiDe Instrument. The gas sorption isotherms were performed with an automatic volumetric adsorption apparatus Micromeritics 3-Flex. Before the adsorption test, **HOF-101**, **HOF-**

101-CH_3, and **HOF-ZSTU-5** were degassed on Micromeritics VacPrep061 at 90 °C until pressure stabilization. Prior to conducting water adsorption measurements, distilled water is rapidly frozen in liquid nitrogen. Subsequently, it undergoes at least three degassing cycles under dynamic vacuum conditions to thoroughly remove residual gases from the water storage vessel. The vapor adsorption container is then heated to 40 °C, and degassing is repeated six times or until the pressure falls below 55 mmHg.

2. Synthesis of HOF-101, HOF-101-CH_3, and other HOFs

2.1. Synthesis of HOF-101 (also called PFC-1)

The synthesis steps were reported in previous literature.[1,2] TBAPy (200 mg) was dissolved in 25 ml of DMF at 120 °C and then filtered. Then 100 mL of MeOH was slowly poured into it under stirring, continued for 10 minutes and then left to stand overnight. The product was centrifuged and immersed in PA for two days, during which time the solution was continuously changed. The yield of the thus obtained product was approximately 60% (based on TBAPy-pristine, CAUTION: highly susceptible to loss during sample collection!).

For single crystal samples: TBAPy (25 mg) was dissolved in 3 ml of DMA and heated at 120 °C until the solution was clarified, filtered while still hot and then cooled. The glass vial containing the solution was allowed to stand in acetone vapour for a fortnight to obtain yellow rod-shaped crystals. The yield of the thus obtained product was approximately 32% (based on TBAPy-pristine).

2.2. Synthesis of HOF-101-CH_3 (also called HOF-101-CH_3)

The synthesis steps were reported in previous literature.[3] TBAPy-2-CH_3 (300 mg) was dissolved in 10 ml of DMF at 80 °C and then filtered. Then 80 ml of MeOH was slowly poured into it under stirring, continued for 10 minutes and then left to stand overnight. The product was centrifuged and immersed in PA for two days, during which time the solution was continuously changed. The yield of the thus obtained product was approximately 83% (based on TBAPy-2-CH_3-pristine, CAUTION: highly susceptible to loss during sample collection!).

For single crystal samples: TBAPy-2-CH_3 (10 mg) was dissolved in a 5 ml vial containing 1 ml of DMF and left open to diffuse for three weeks in a sealed container containing 5 ml of MB to obtain yellow needle crystals. The yield of the thus obtained product was approximately 72% (based on TBAPy-2-CH_3-pristine).

2.3. Synthesis of HOF-ZSTU-5

The synthesis pathway for TBAPy-3-CH_3 is as follows[4]:

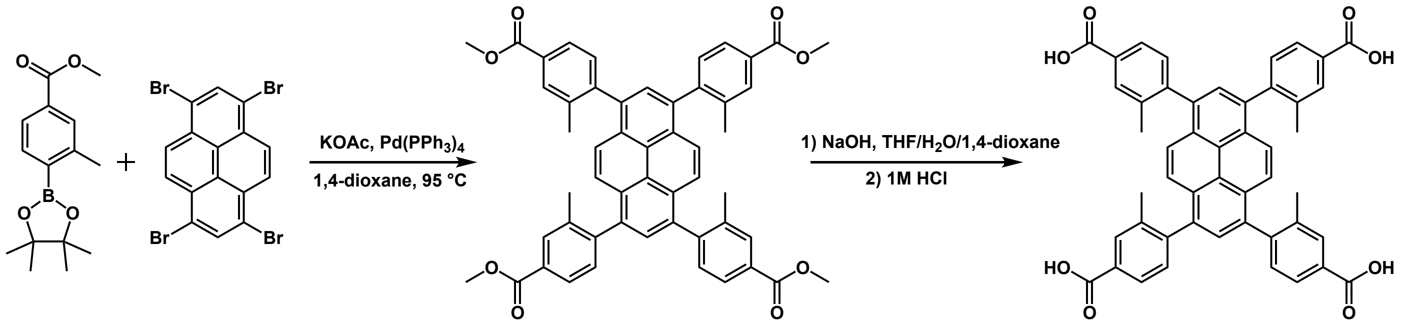

Although we purchased the ligand for laboratory use, its synthesis is well-established in the literature. It follows a convergent route involving a standard bromination of pyrene (an inexpensive coal-tar derivative) followed by a Suzuki-Miyaura coupling with a benzene derivative. As Suzuki coupling is a mature reaction widely used in the fine chemical industry, the synthesis is highly scalable and cost-effective for mass production.

Interfacial diffusion growth: TBAPy-3-CH_3 (100 mg) was added to 6 mL of MeOH with slight shaking to dissolve and the solution was filtered. Then 20mL of n-hexane or cyclohexane was spread on top of the solution and placed in an oven at 60 °C for at least 3 hours to obtain light yellow-green rhombic crystals. The yield of the thus obtained product was approximately 76% (based on TBAPy-3-CH_3-pristine). Notably, crystal formation was observed at the interface of the solution layering after a few minutes at room temperature.

Direct mixing synthesis: It is also possible to mix the MeOH solution with the dissolved TBAPy-3-CH_3 directly with the n-hexane or cyclohexane solvent keeping it at 60 °C for several hours or leaving it at room temperature overnight, which gives crystals of smaller size, mostly in the form of polyhedral prisms. The yields thus obtained are essentially the same as the synthesis method described above.

Gaseous diffusion growth: 50 mg of TBAPy-3-CH_3 was dissolved in 6 mL of MeOH and then filtered and transferred to a 20 mL open-topped vial, which was then left to stand overnight in a sealed environment at room temperature containing 10 mL of n-hexane or cyclohexane. The yield of the thus obtained product was approximately 90% (based on TBAPy-3-CH_3-pristine).

Regeneration of HOF-ZSTU-5: High quality single crystals were obtained by adding 50 mg of **HOF-ZSTU-5** sample to 5 mL of MeOH (recovered mother liquor/new liquor = 1/1, v/v) and allowing it to fully dissolve

at 60 °C. After filtration, the filtration was placed in a sealed vial containing 20 mL of n-hexane or cyclohexane overnight.

2.4. Synthesis of other HOFs

The synthesis of **HOF-11**,[5] **HOF-14**,[6] **HOF-16**,[7] **HOF-BTB**,[8] **HOF-TCBP**,[9] **HOF-ZSTU-1~3**,[10] **HOF-30**,[11] and **HOF-FJU-1**[12] were performed according to the original literature.

3. Fitting of single component gas adsorption isotherm

The C_2H_4 and C_2H_6 sorption isotherms collected at 298 K were fitted on the basis of the dual-site Langmuir-Freundlich equation or its variants:

$$q = q_{A,sat}\frac{b_A p^{vA}}{1 + b_A p^{vA}} + q_{B,sat}\frac{b_B p^{vB}}{1 + b_B p^{vB}}$$

with T-dependent parameters b_A and b_B:

$$b_A = b_{A0} exp\left(\frac{E_A}{RT}\right); \; b_B = b_{B0} exp\left(\frac{E_B}{RT}\right)$$

Where p is the pressure of the bulk gas at equilibrium with the adsorbed phase (kPa), q is the adsorbed amount per mass of adsorbent (mmol g^{-1}), $q_{A,sat}$ and $q_{B,sat}$ are the saturation capacities of sites A and B(mmol g^{-1}), b_A and b_B are the affinity coefficient, vA and vB are the Langmuir-Freundlich index (dimensionless).

4. IAST calculations of adsorption selectivity

For the separation of binary mixtures of components C_2H_6/C_2H_4, the adsorption selectivity is defined by the following equation:

$$S_{ads} = \frac{q_1/q_2}{p_1/p_2}$$

In the equation, q_1 and q_2 are the molar loadings in the adsorbed phase in equilibrium with the bulk gas phase with partial pressures p_1 and p_2.

5. Isosteric heat of adsorption *(Q_{st})*

A virial-type expression of comprising the temperature-independent parameters a_i and b_j was employed to calculate the enthalpies of adsorption for gas molecules at 273 and 298 K. In each case, the data were fitted using the equation:

$$\ln P = \ln N + \frac{1}{T}\sum_{i=0}^{m} a_i N^i + \sum_{i=0}^{n} b_i N^i$$

Where N is gas uptake (mg g^{-1}), P is pressure (mmHg), a and b are the virial coefficients, m and n are the number of coefficients required to adequately describe the isotherm. The values of the virial coefficients a_0 through a_m were then used to calculate the isosteric heat of absorption using the following expression:

$$Q_{st} =- R\sum_{i=0}^{m} a_i N_i$$

which R is the universal gas constant.

6. GCMC simulations

Grand Canonical Monte Carlo (GCMC) simulations of **HOF-101**, **HOF-101-CH_3**, and **HOF-ZSTU-5a** were performed using the sorption module. During the simulation, the three frameworks, C_2H_6, and C_2H_4 molecules are considered rigid. The simulations were carried out at 298 K, adopting the fixed pressure task, Metropolis method, and the Universal field. The electric charge used is Gasteiger. All three HOFs used the 1 × 1 × 1 cell. The interaction energy between hydrocarbon molecules and framework were computed through the Coulomb and Lennard-Jones (LJ) potentials. The cutoff radius was chosen as 15.5 Å for the LJ potential and the long-range electrostatic interactions were handled using the Ewald & Group summation method, the summation method for van der Waals interactions is atomic based. The loading step, equilibration step and production step are all 1×10^6.

The grand canonical Monte Carlo (GCMC) simulations were performed in the *NVT* ensemble to calculate the isosteric heats of adsorption Q_{st}. The internal energy ΔU was computed during the simulation, which is directly related to Q_{st}. The isosteric heat of adsorption Q_{st} was calculated from:

$$Q_{st} = RT - \frac{\langle U_{ff}N\rangle - \langle U_{ff}\rangle\langle N\rangle}{\langle N^2\rangle - \langle N\rangle\langle N\rangle} - \frac{\langle U_{sf}N\rangle - \langle U_{sf}\rangle\langle N\rangle}{\langle N^2\rangle - \langle N\rangle\langle N\rangle}$$

where R is the gas constant, N is the number of molecules adsorbed, and ⟨ ⟩ indicates the ensemble average. The U_{ff} in the first and second terms are the contributions from the molecular thermal energy and adsorbate-adsorbate interaction energy, respectively. The U_{sf} in the third term is the contribution from the adsorbent–adsorbate interaction energy.

7. Density-functional theory (DFT-D) calculations

The first-principles DFT-D simulations of the static binding energy were performed using the $DMol^3$

module.[13,14] The electrostatic potential mapped to the Connolly surface is also calculated using this module. The Generalized Gradient Approximation (GGA) with the Perdew-Burke-Ernzerhof (PBE) functional was used to perform all-electron spin-unrestricted DFT calculations. A semiempirical addition (the TS method) of dispersive forces to the conventional DFT was included in the calculation to account for van der Waals interactions. The energy, force and displacement convergence criterions were set as 1×10^{-5} Ha, 2×10^{-3} Ha and 5×10^{-3} Å, respectively. The double numerical including polarization (DNP) basis set was chosen for all atoms. To accelerate convergence, an SCF tolerance value of 1.0 e^{-4} and a smearing value of 0.05 Ha were used. The static binding energy (at T = 0 K) was then calculated using $E_B = E_{(MOF)} + E_{(gas)} - E_{(MOF + gas)}$.

8. Electron Density Difference (EDD) calculations

The calculation of the charge displacement between the host and the guest was carried out using the CASTEP module.[15,16] The Generalized Gradient Approximation (GGA) with the Perdew-Burke-Ernzerhof (PBE) functional was used to perform all-electron spin-unrestricted DFT calculations. The energy cutoff used for the plane wave basis set was as of 571.40 eV, the threshold used to determine SCF convergence was set to -10^{-6} eV $atom^{-1}$, and the maximum number of SCF cycles was set to 100. The unit energy tolerance was specified to be calculated as the energy change per atom. The number of integration points used to integrate the wavefunction in reciprocal space was set to 1 × 1 × 1.The type of pseudopotentials used was the default OTFG ultrasoft combined with Koelling-Harmon relativistic treatment. Fixing three electronic occupation numbers during electronic minimization is checked.

9. Dynamic breakthrough experiments

Dynamic breakthrough experiments under dry and near-saturation humidity conditions were performed on a home-made separation and analysis apparatus consisting of two stainless steel columns, at 298 K and 1 bar. One column was filled with activated HOF (0.25/0.548 g for **HOF-ZSTU-5a**, 0.25 g for **HOF-101**, and 0.56 g for **HOF-101-CH_3**) at room temperature for adsorption and the other column was used to stabilize the flow rate and to obtain the original composition of the gas mixture used prior to the experiment. Considering the distinct physical appearances of the three HOFs (**HOF-101** and **HOF-101-CH_3** are agglomerated flakes, while **HOF-ZSTU-5a** is fully crystalline), samples for breakthrough experiments underwent uniform mild physical grinding to reduce interparticle voids during column packing while avoiding excessive back pressure. The length of the adsorption bed is 200 mm, and the inner diameter of the adsorption bed is 4 mm. The adsorption column was placed in a thermostat to maintain a constant temperature. The adsorption column inlet and outlet

flow rates were controlled simultaneously by a manometer and a mass flow meter. During the experiment, the adsorption column outgas was monitored with a gas chromatograph (GC-2014C Shimadzu) equipped with a hydrogen flame ionization detector (FID). To continue the breakthrough experiments, at 50 °C, the adsorption column was purged with Argon at a flow rate of 10 mL min^{-1} for one hour to desorption.

10. C_2H_4 productivity calculations

The C_2H_4 productivity calculations of the three HOFs on the fixed bed can be determined as follows:

$$q_i = \frac{C_i V}{m} \times \int_0^t \left(1 - \frac{F}{F_0}\right) dt$$

Where q_i is the equilibrium adsorption capacity of gas i (cm^3 g^{-1}), C_i is the feed gas concentration, V is the volumetric feed flow rate (mL min^{-1}), t is the adsorption time (min), F_0 and F are the inlet and outlet gas molar flow rates, respectively, and m is the mass of the adsorbent (g).

11. Single Crystal X-ray Diffraction (SCXRD) measurement

Single crystal X-ray diffraction data were collected from a Pilatus XtaLAB P300DS with Cu Kα (λ =1.54184 Å) micro-focus X-ray sources at 293 K. Data collection and reduction were carried out in CrysAlisPro 1.171.39.46 (Rigaku Oxford Diffraction, 2015). The structure solution and refinement were performed with SHELXL-2018 and Olex2 1.5 program.[17] The structure was solved by direct methods and was refined against F2 by full-matrix least-squares techniques. All non-hydrogen atoms were refined with anisotropic displacement parameters.

SQUEEZE function from the PLATON program was used to remove disordered solvent molecules in voids. Data collection and refinement details are listed in Table S1-3 and supplementary crystallographic CIF data have been deposited on Cambridge Crystallographic Data Centre (CCDC) with the number 2425854 for **HOF-ZSTU-5**, 2425853 for **HOF-ZSTU-5a**, 2425855 for 150 K C_2H_4@**HOF-ZSTU-5a**, 2425856 for 150 K C_2H_6@**HOF-ZSTU-5a**, 2425857 for RT C_2H_4@**HOF-ZSTU-5a**, and 2425858 for RT C_2H_6@**HOF-ZSTU-5a**.

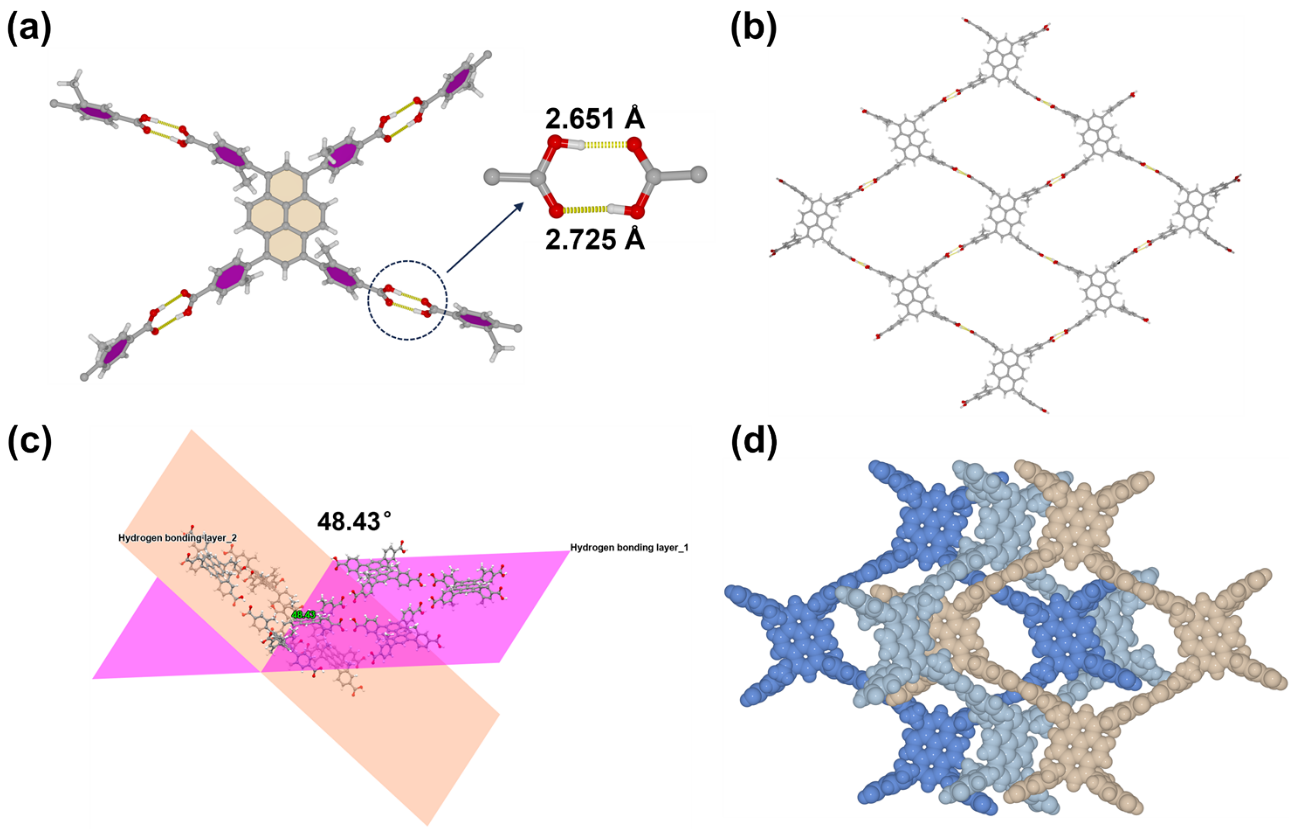

Fig. S1. (a) Hydrogen-bonding linkages and bond lengths of the TBAPy-3-CH_3 in **HOF-ZSTU-5**. (b) Layered hydrogen-bonding network formed by extension of the precursors through hydrogen-bonding units. (c) Dihedral angle (48.43°) between two interpenetrating hydrogen-bonded layers. (d) The 3-fold interpenetrating structure of **HOF-ZSTU-5**.

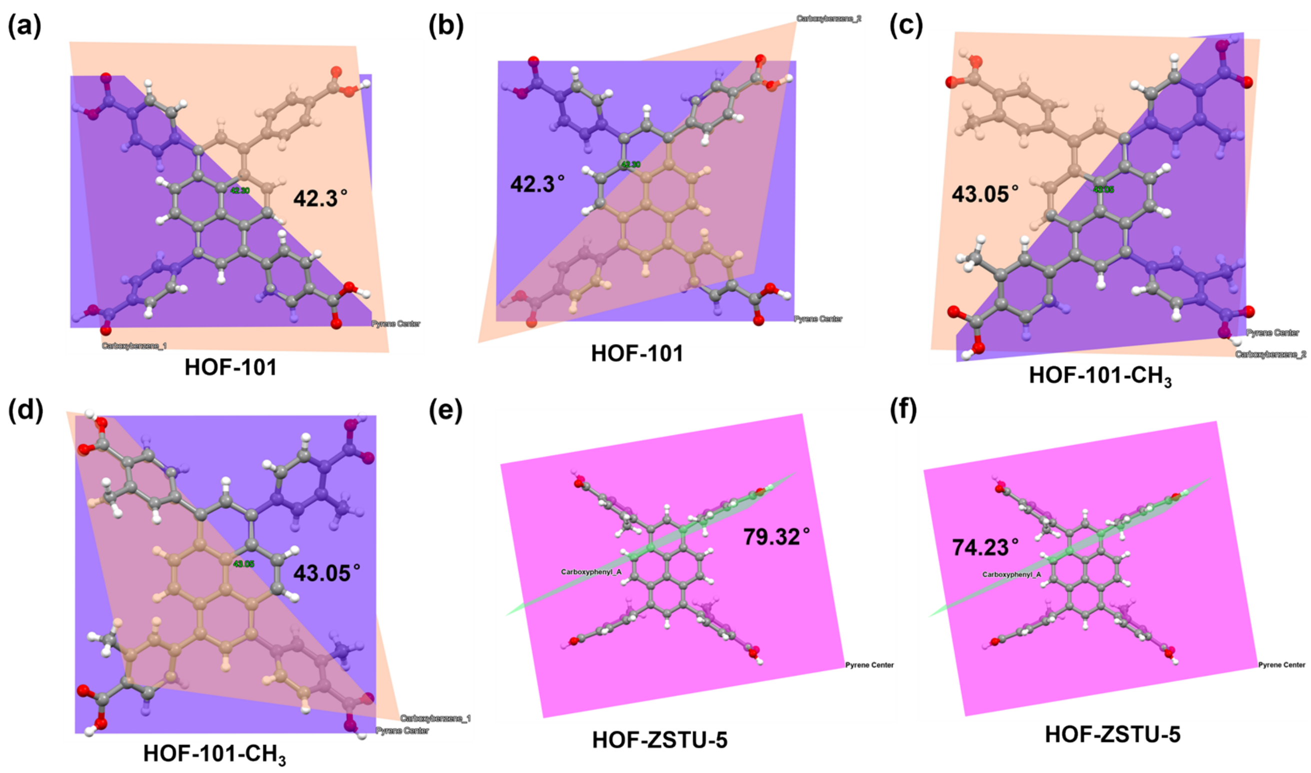

Fig. S2. The dihedral angle between the carboxyphenyl and the pyrene core at adjacent positions of the three HOFs. (a) and (b) **HOF-101**. (c) and (d) **HOF-101-CH$_3$**. (e) and (f) **HOF-ZSTU-5**. Carboxyphenyls in the symmetrical direction have the same properties.

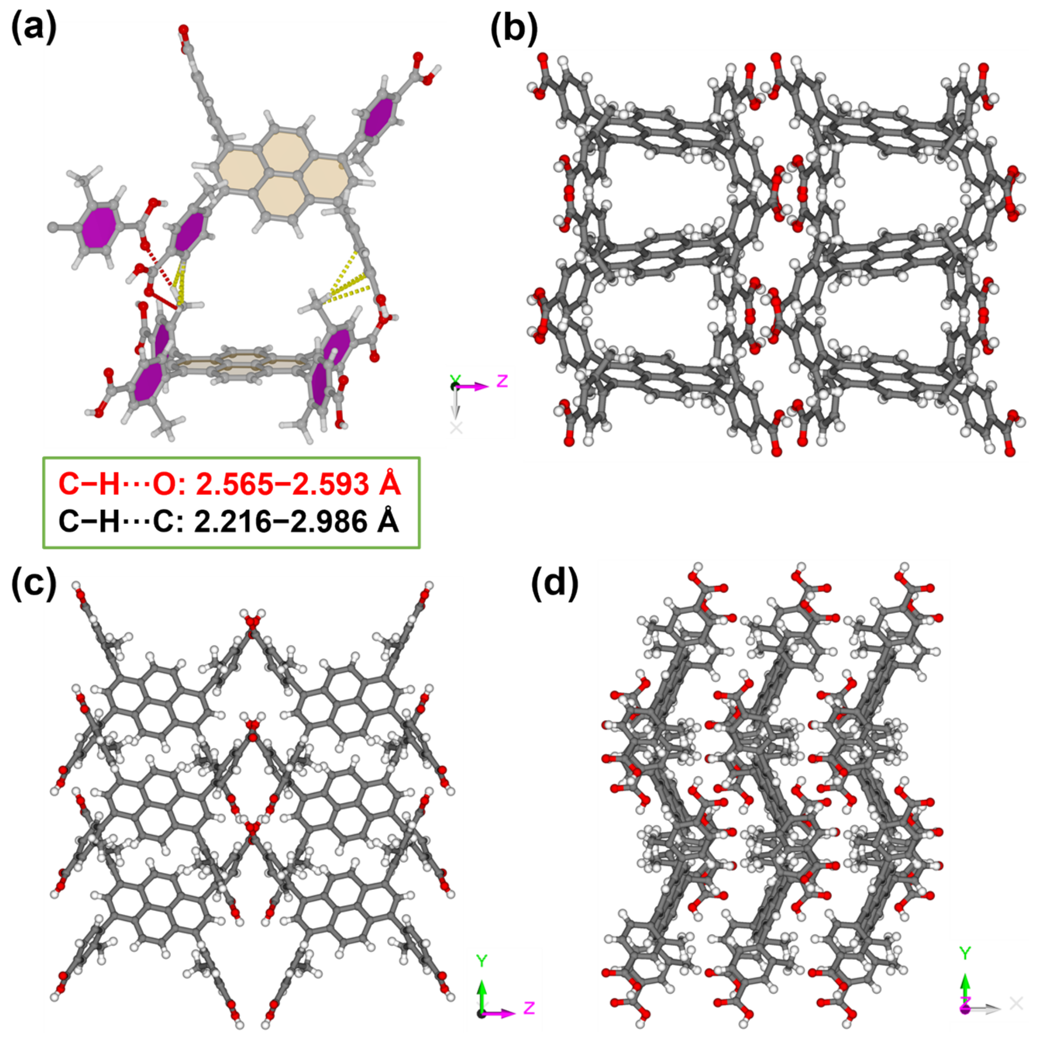

Fig. S3. (a) Multiple hydrogen bonds and strong van der Waals forces between the building units of **HOF-ZSTU-5** (The interaction forms have symmetry at diagonal positions). (b-d) The framework view of **HOF-ZSTU-5** in three directions.

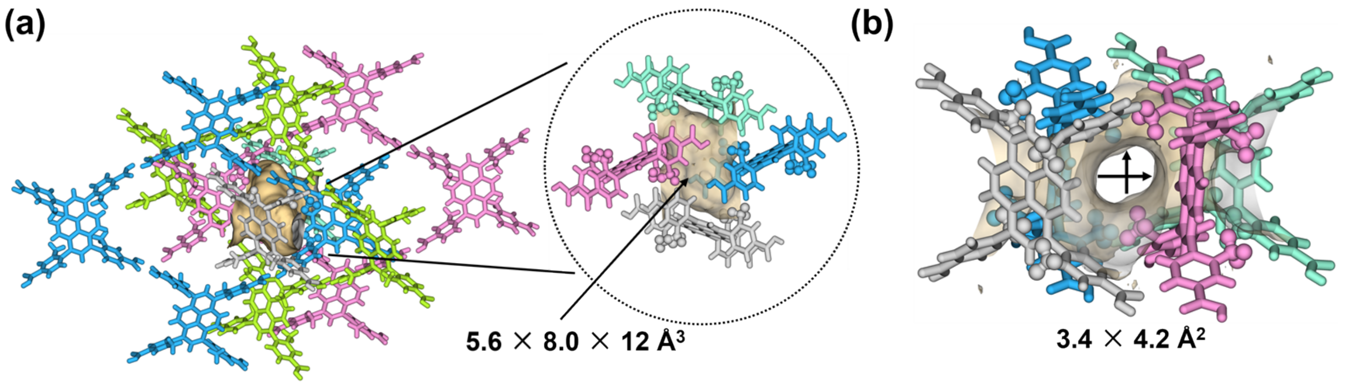

Fig. S4. Pore structure of **HOF-ZSTU-5**. (a) four-directionally crosslinked cavity and its size in the 3-fold interpenetrating hydrogen-bonding network. (b) The opening size between two connected cross-linked cavities.

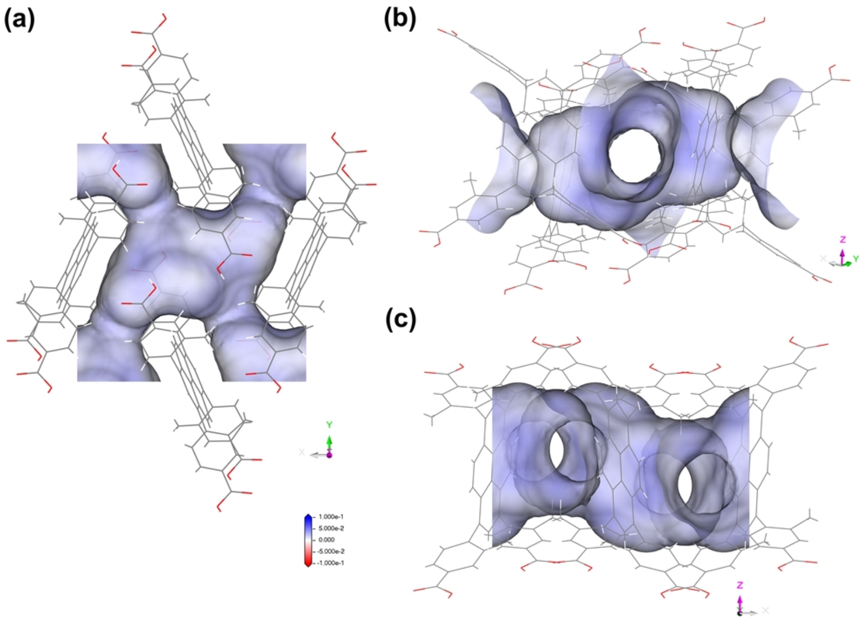

Fig. S5. Pore environment maps of the electrostatic potential were mapped on the Connolly surface (probe radius 1.84 Å) of **HOF-ZSTU-5**, and the cavity was observed from three different angles. (a) Top view of the crosslinked cavity body. (b) Observation of the cavity opening at the side. (c) Observation of the four openings of the cavity from the y-axis direction, shown in semi-transparent.

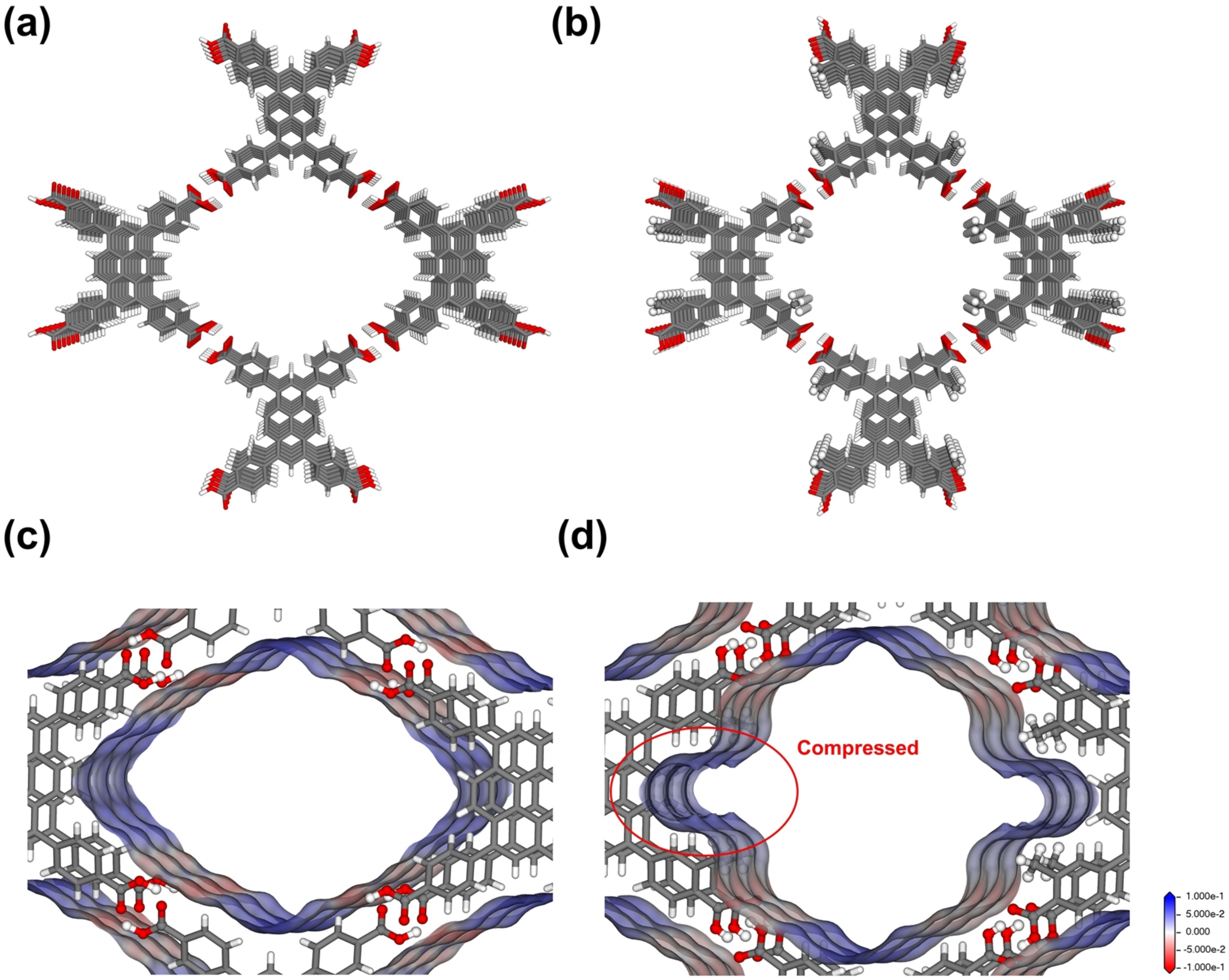

Fig. S6. As a comparative validation of the pore environment in homologous HOFs based on π–π stacking. The 1D pore diagrams of (a) **HOF-101** and (b) **HOF-101-CH₃**. The corresponding pores are formed by interconnecting precursors to form planar hydrogen-bonded layers and then stacked by interlayer conjugation. Mapping the electrostatic potential onto the Connolly surfaces (probe radius of 1.84 Å) of (c) **HOF-101** and (d) **HOF-101-CH₃** shows a pore environment with a spaced distribution of polar and nonpolar regions, where **HOF-101-CH₃** compresses the nonpolar region on the pyrene sidewall due to the introduction of *ortho*−CH_3.

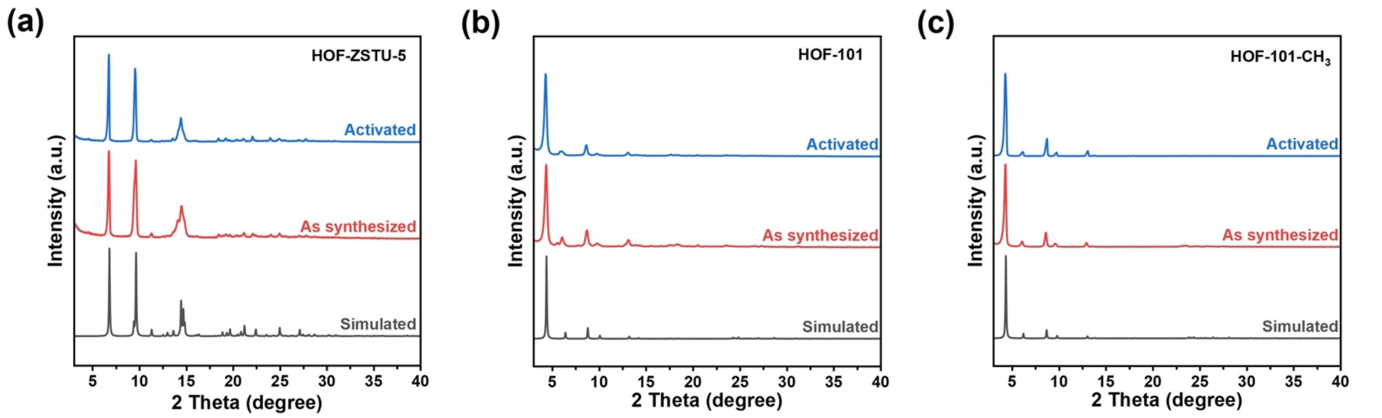

Fig. S7. PXRD spectra of the synthesized and activated samples compared to crystal simulation data: (a) **HOF-ZSTU-5**, (b) **HOF-101**, and (c) **HOF-101-CH$_3$**.

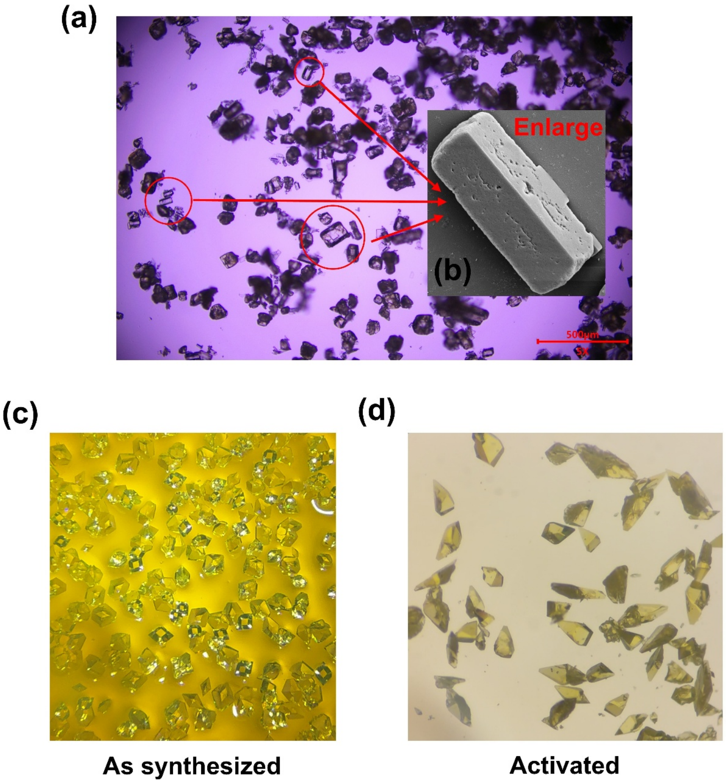

Fig. S8. (a) A sample of **HOF-ZSTU-5** prepared by the direct mixing method was photographed using an optical microscope with a snapshot-taking function, and a large image is shown as the backing. (b) Sample image of **HOF-ZSTU-5** crystals prepared by the direct mixing method obtained using SEM. Snapshots of (c) synthesized and (d) activated **HOF-ZSTU-5** synthesized by interfacial or gaseous diffusion growth taken directly under an optical microscope.

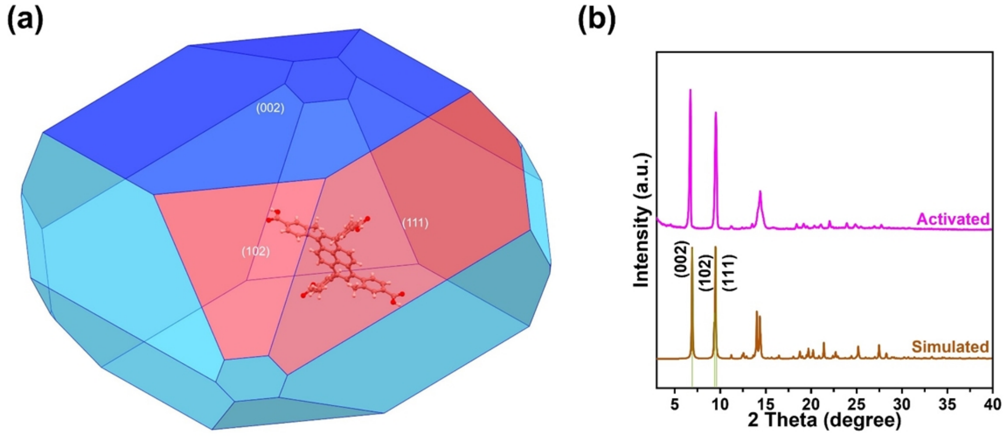

Fig. S9. (a) Crystal morphology of **HOF-ZSTU-5** obtained by simulation based on the BFDH algorithm. The predicted crystal morphology coincides with the crystal morphology obtained by interfacial or gaseous diffusion growth. (b) Correspondence between the simulated crystal morphology facet data and PXRD curves.

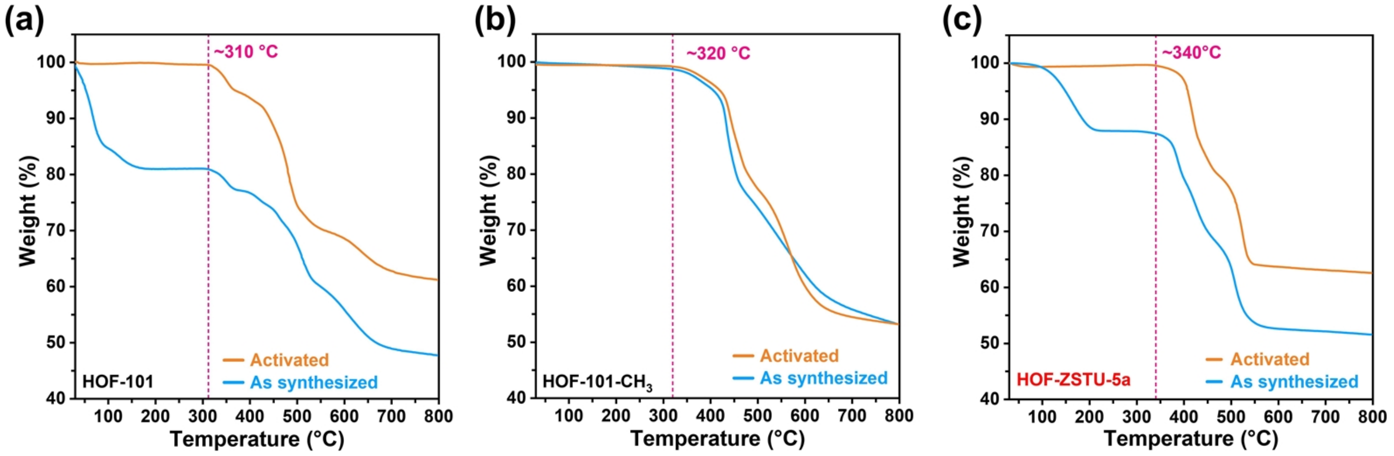

Fig. S10. TGA curves for (a) **HOF-101**, (b) **HOF-101-CH_3** and (c) **HOF-ZSTU-5**. For the TGA curve of **HOF-ZSTU-5**, the weight loss in the initial phase is due to the contact of the activated sample with air.

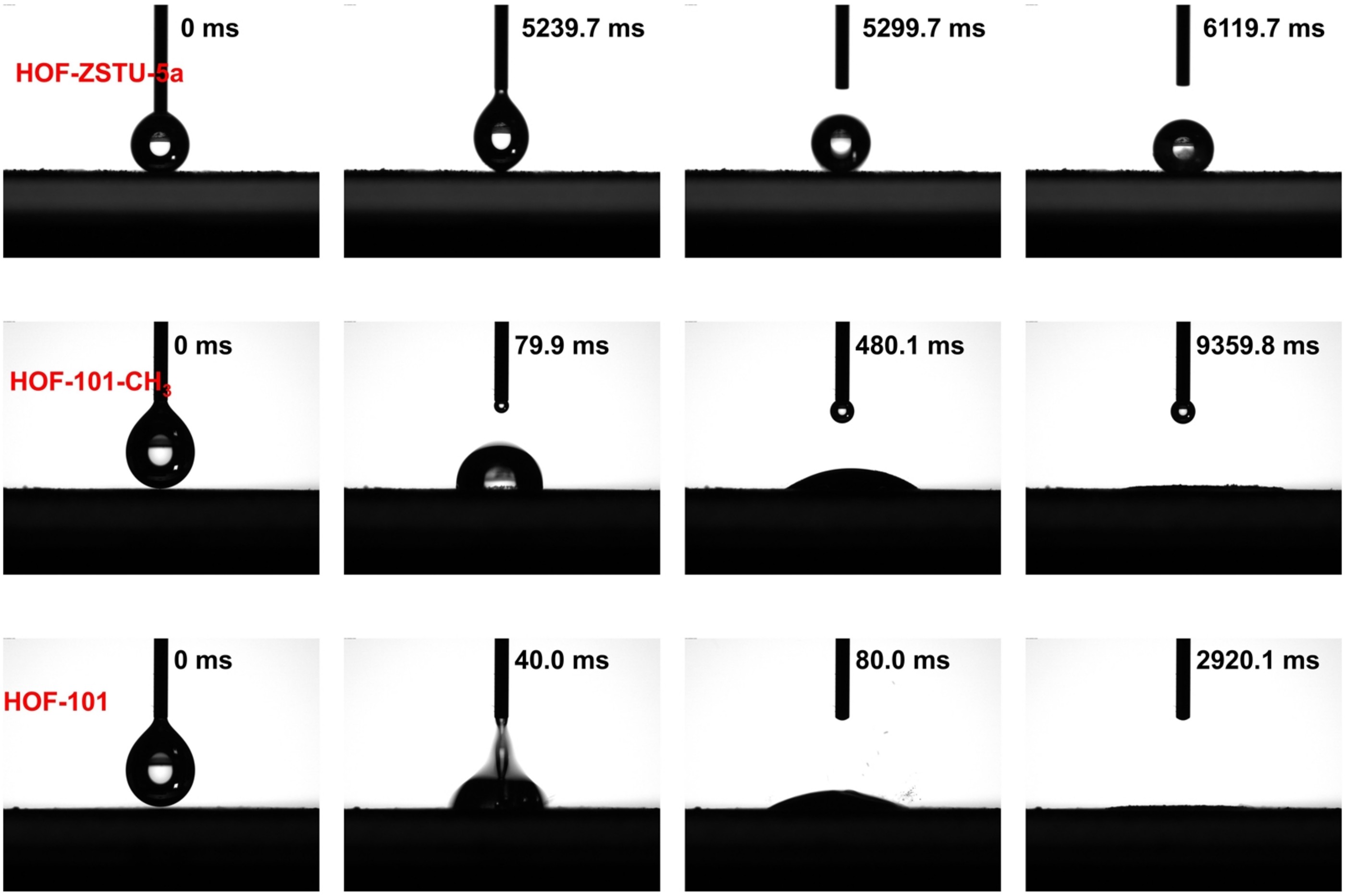

Fig. S11. Transient snapshots of the water contact angle test for **HOF-ZSTU-5**, **HOF-101-CH$_3$**, and **HOF-101**. Each snapshot, from left to right, represents the state of the water droplet before contacting the sample, the first contact with the sample, the infiltration process, and the end of infiltration, and includes the relative instantaneous time. For the superhydrophobic **HOF-ZSTU-5**, due to the strong water repellency of the sample surface, the needle was lifted to force the water droplets to fall onto the sample surface to continue the test. For hydrophilic HOFs, the time required to complete infiltration was calculated.

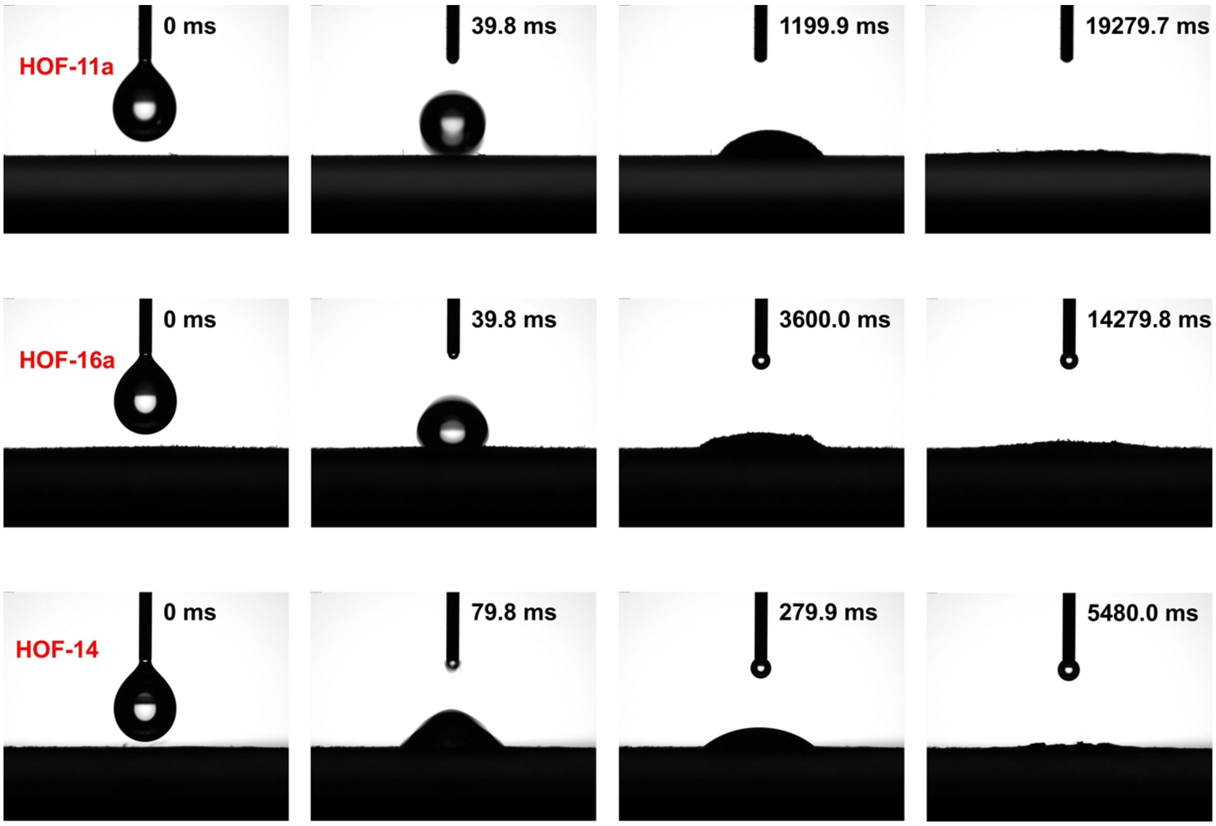

Fig. S12. Transient snapshots of the water contact angle test for **HOF-11a**, **HOF-16a**, and **HOF-14**.

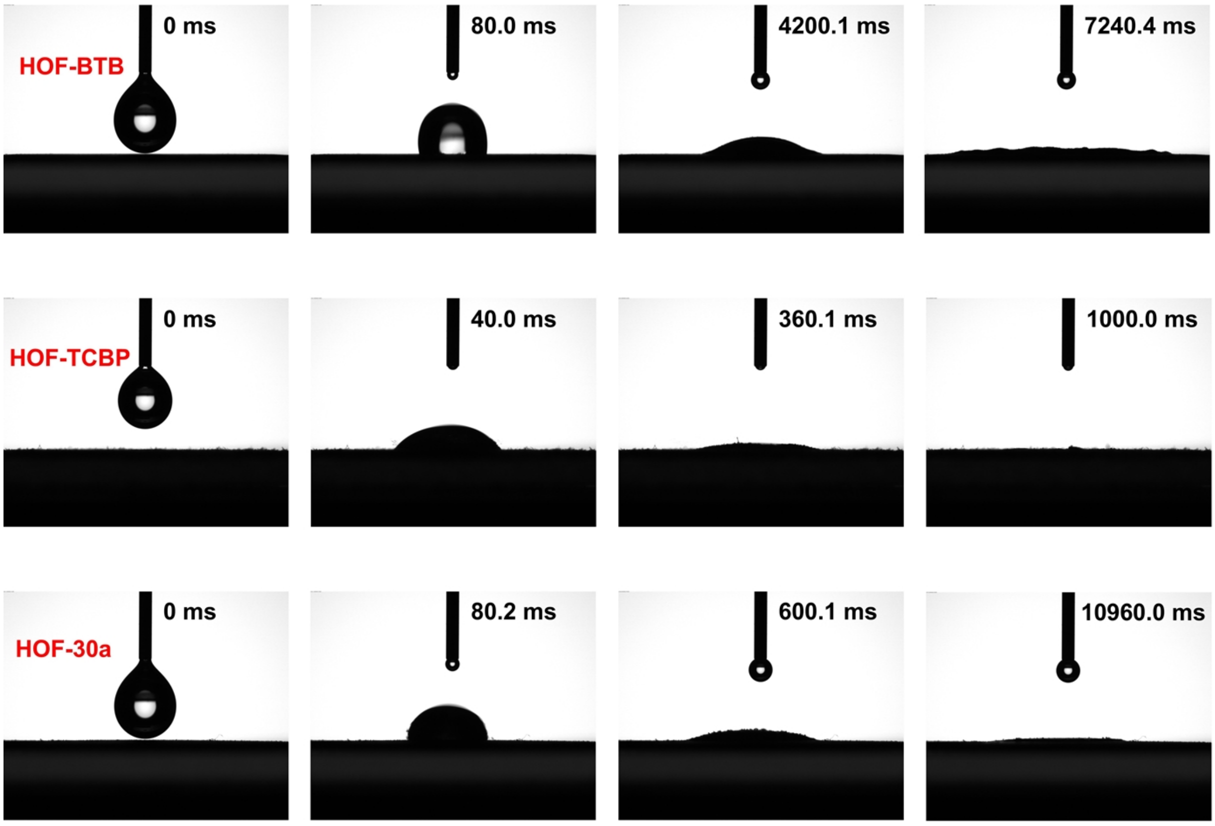

Fig. S13. Transient snapshots of the water contact angle test for **HOF-BTB**, **HOF-TCBP**, and **HOF-30a**.

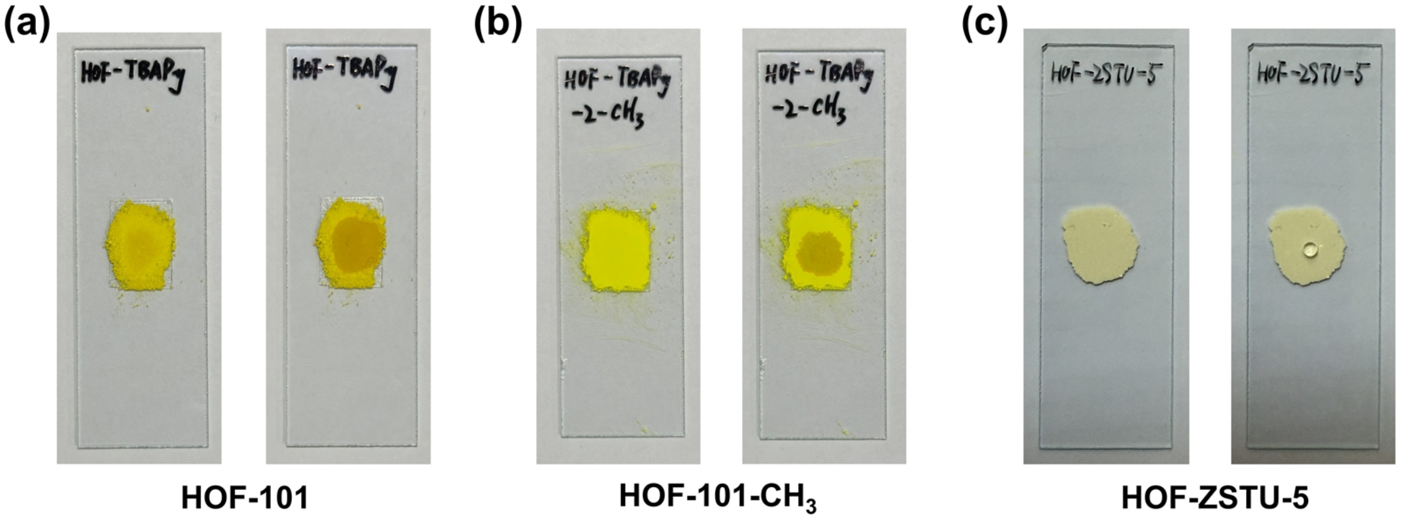

Fig. S14. The snapshots of the sample surfaces before and after the water contact angle test for (a) **HOF-101**, (b) **HOF-101-CH$_3$**, and (c) **HOF-ZSTU-5**. The surface of **HOF-ZSTU-5** hardly observed any extension after contacting a water droplet and the droplet slipped away when the surface was slightly tilted (similar to the hydrophobic effect of lotus leaves).

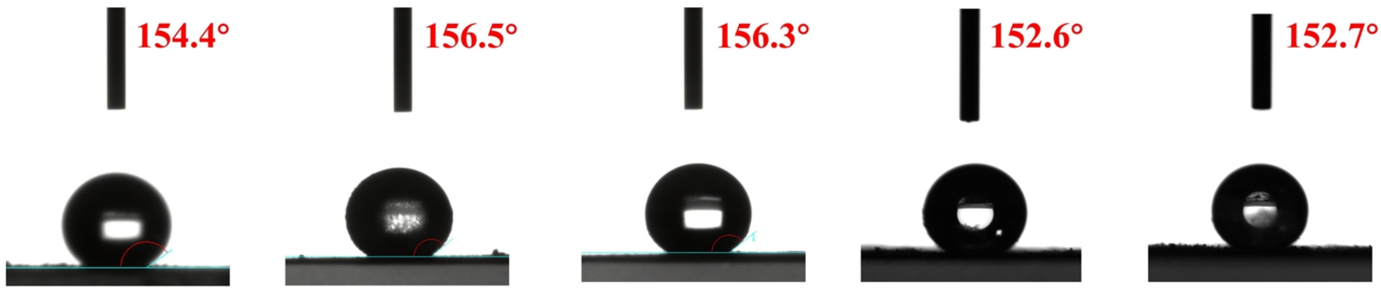

Fig. S15. Water contact angle measurements were repeated five times for **HOF-ZSTU-5**.

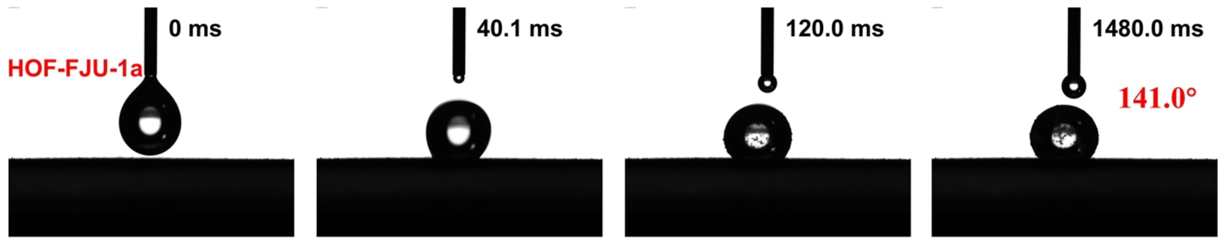

Fig. S16. Transient snapshots of the water contact angle test for **HOF-FJU-1a**. The water repellency of the **HOF-FJU-1a** surface is not as strong as that of the **HOF-ZSTU-5a**, so water droplet can fall normally.

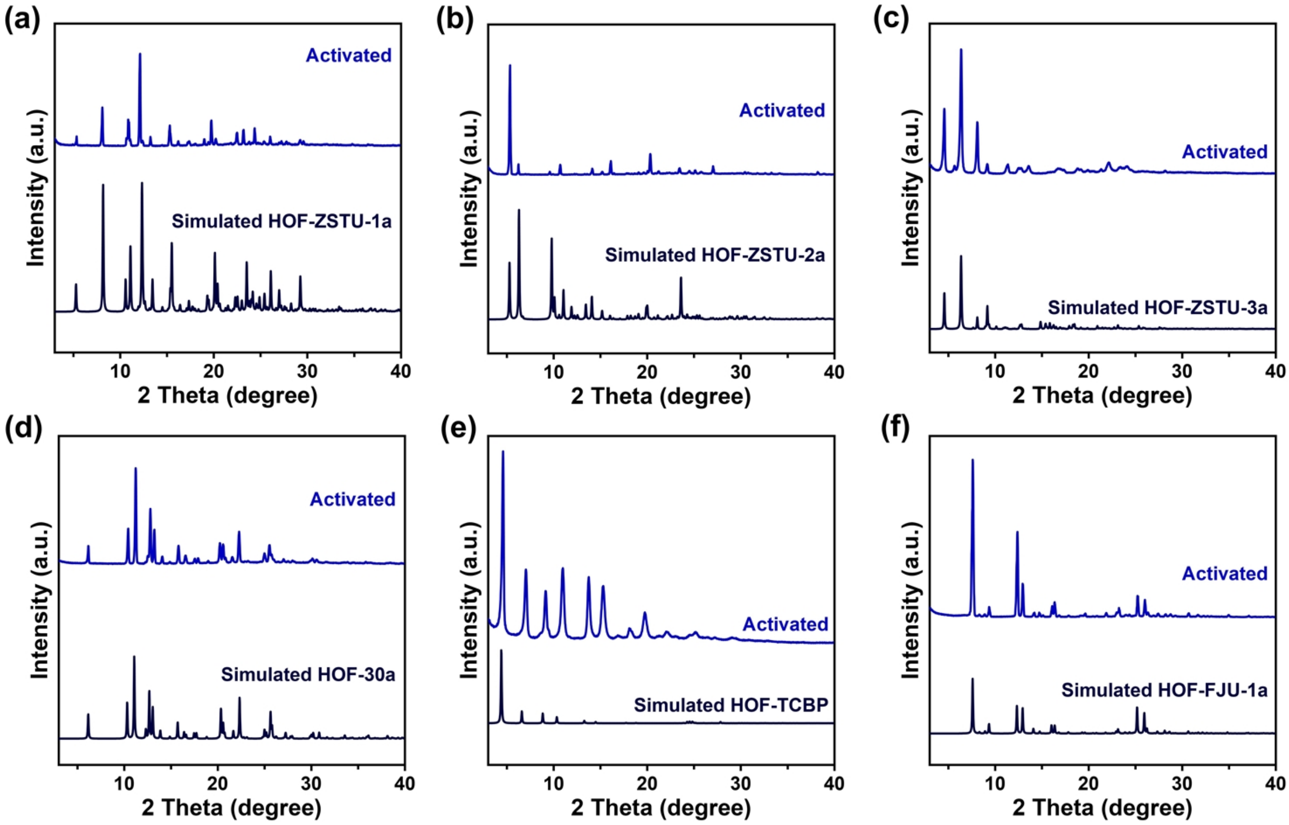

Fig. S17. PXRD of simulated and activated (a) **HOF-ZSTU-1a**, (b) **HOF-ZSTU-2a**, (c) **HOF-ZSTU-3a**, (d) **HOF-30a**, (e) **HOF-TCBP**, and (f) **HOF-FJU-1a**.

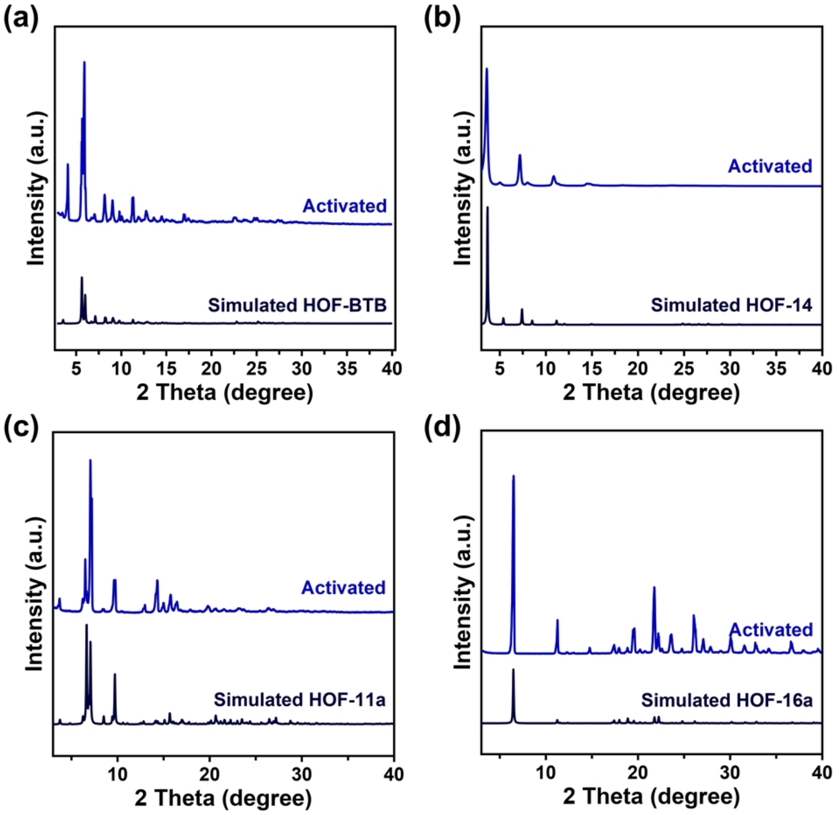

Fig. S18. PXRD of simulated and activated (a) **HOF-BTB**, (b) **HOF-14**, (c) **HOF-11a**, and (d) **HOF-16a**.

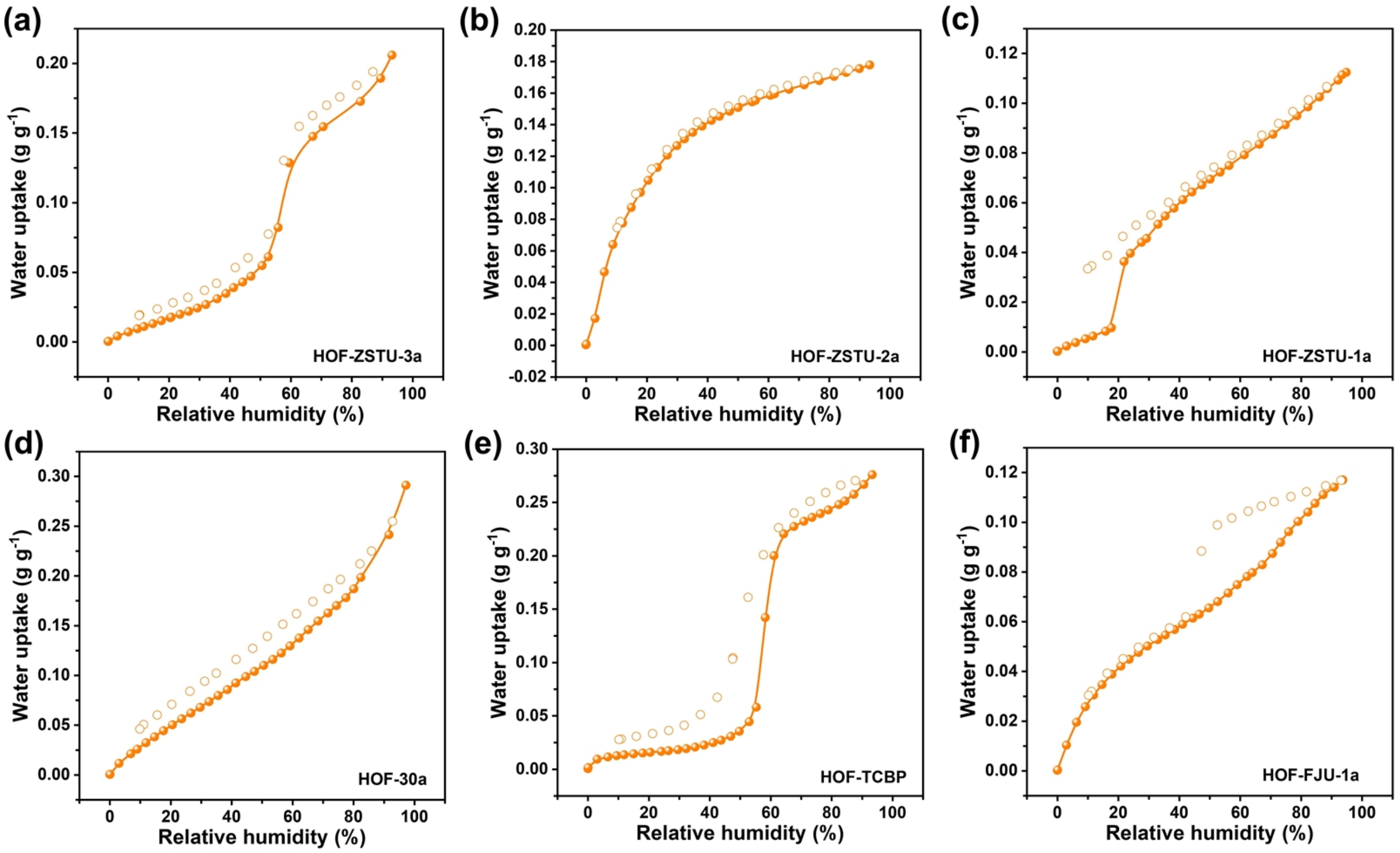

Fig. S19. Water uptakes of (a-e) carboxylic acid-based HOFs and (f) **HOF-FJU-1a** at 298 K.

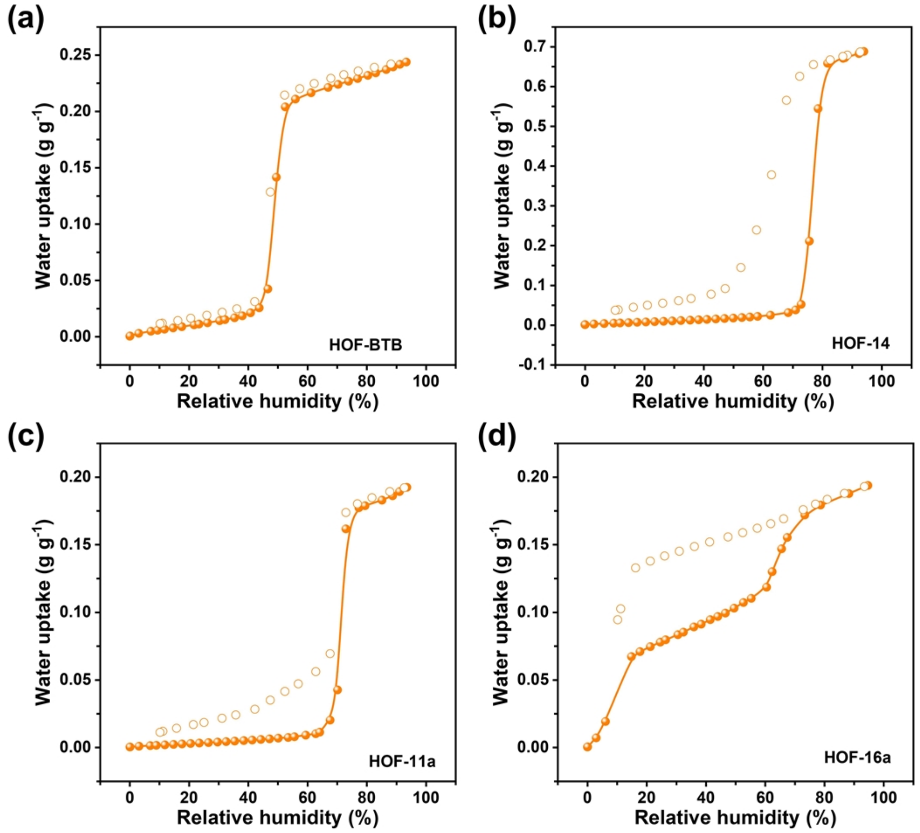

Fig. S20. Water uptakes of carboxylic acid-based HOFs at 298 K.

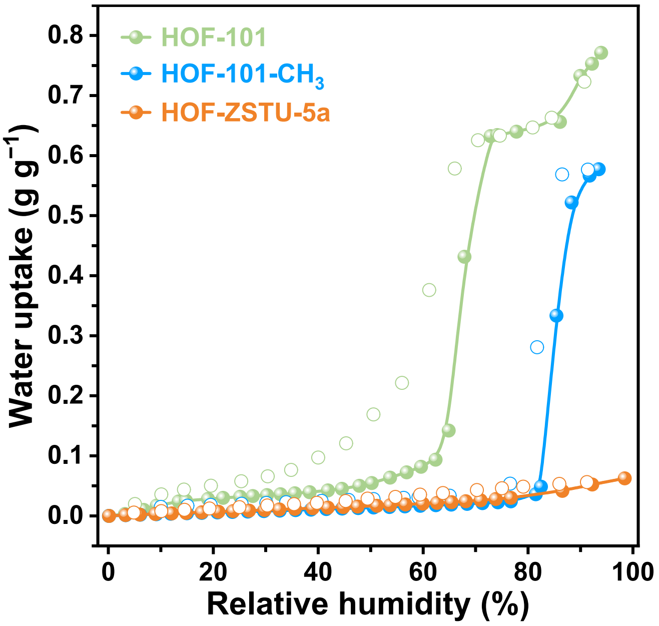

Fig. S21. Water uptakes of **HOF-ZSTU-5a**, **HOF-101** and **HOF-101-CH$_3$** at 298 K.

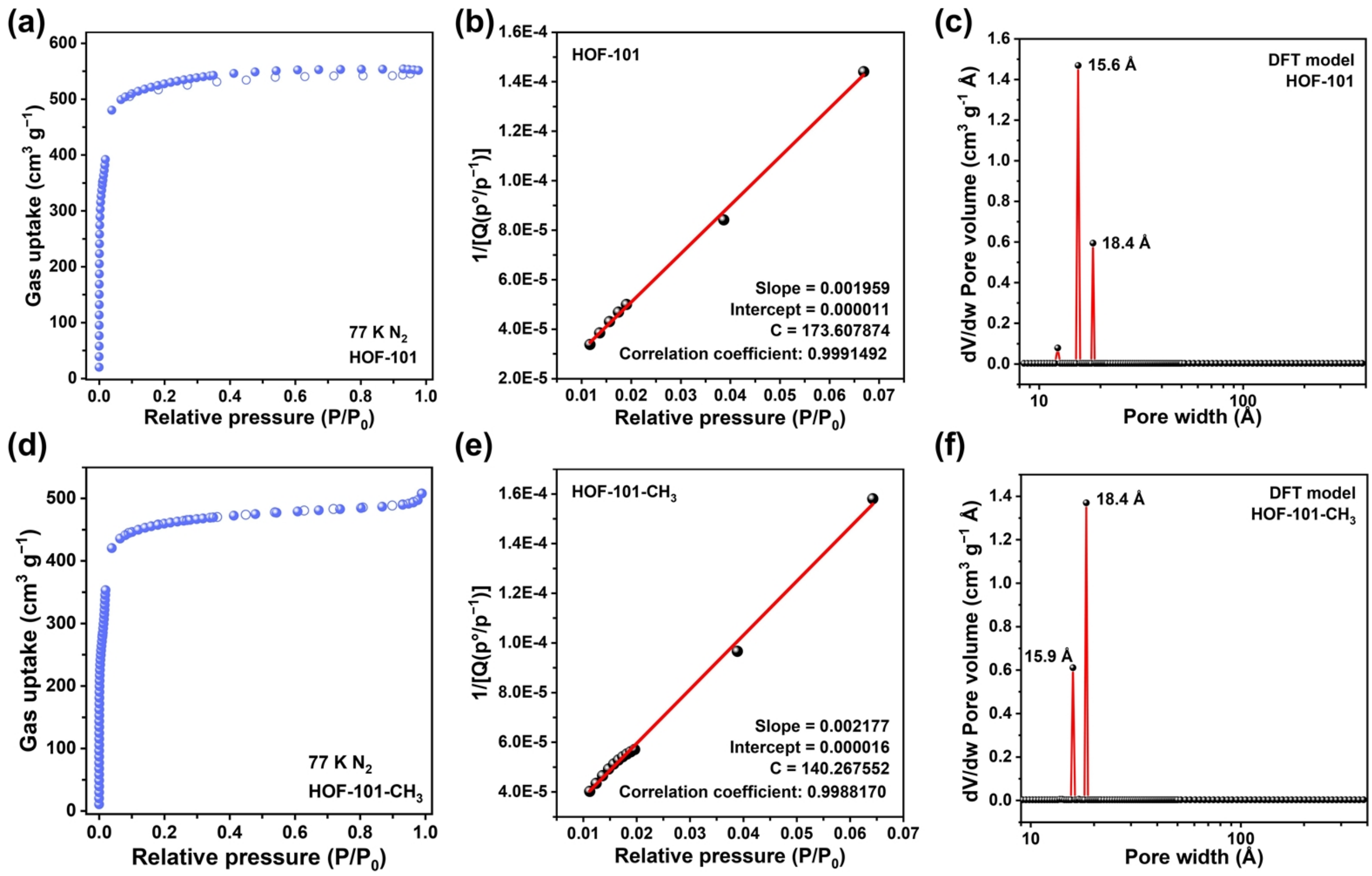

Fig. S22. **HOF-101** of (a) 77 K N_2 sorption isotherm, (b) BET surface area fitting calculations based on the 77 K N_2 isotherm, and (c) pore size distributions obtained using the DFT model. **HOF-101-CH$_3$** of (c) 77 K N_2 sorption isotherm, (d) BET surface area fitting calculations based on the 77 K N_2 isotherm, and (e) pore size distributions obtained using the DFT model.

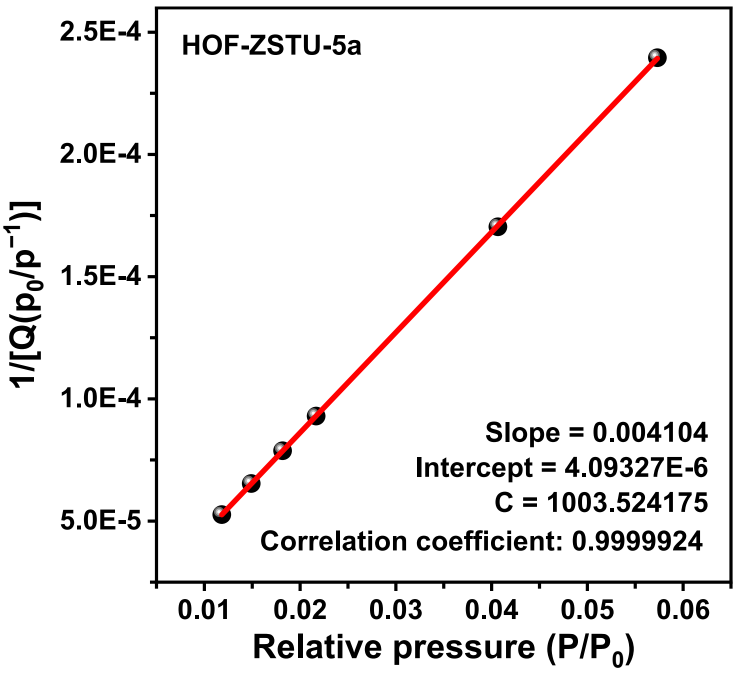

Fig. S23. BET surface area fitting curves for **HOF-ZSTU-5a** using the 77 K N_2 sorption isotherm, taking points in the range of 0.01−0.06 relative pressure.

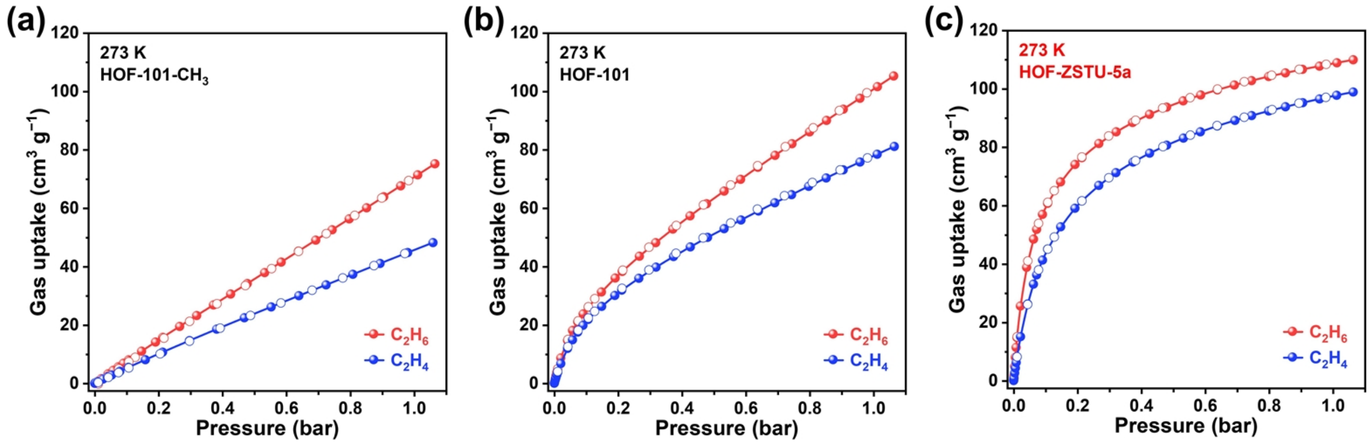

Fig. S24. Single-component gas sorption isotherms at 273 K for (a) **HOF-101-CH$_3$**, (b) **HOF-101**, and (c) **HOF-ZSTU-5a**.

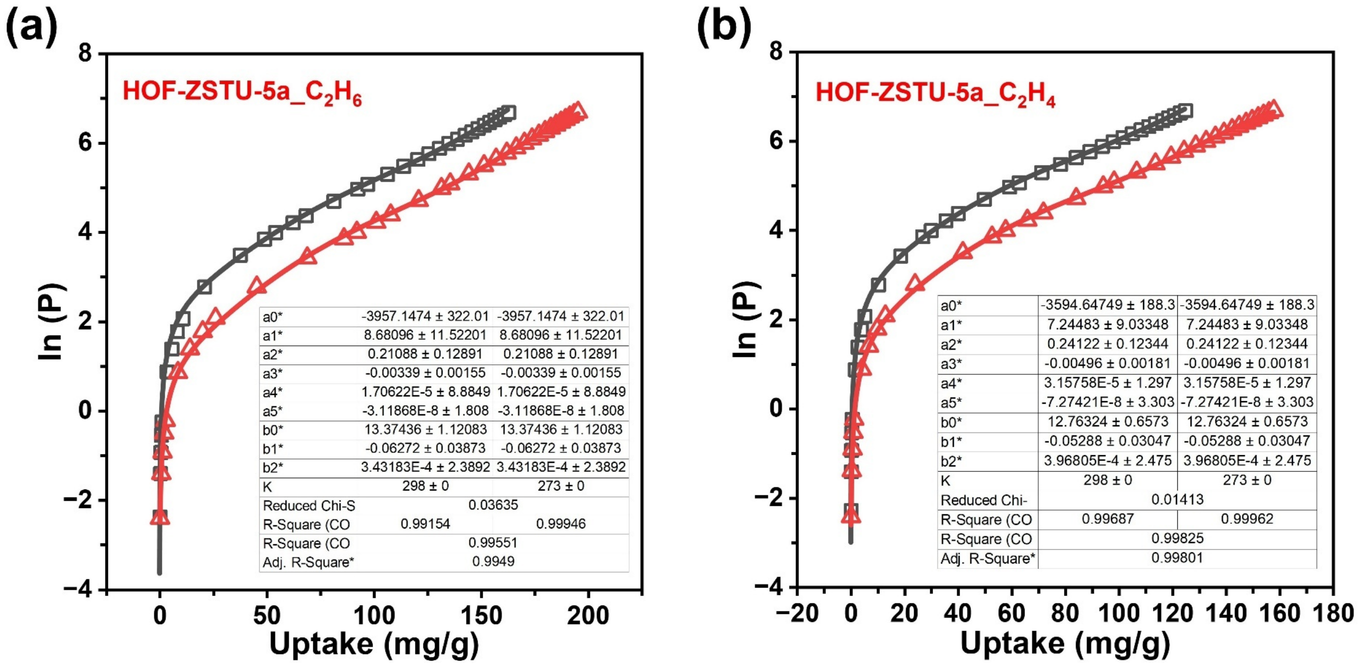

Fig. S25. The fitting results of Q_{st} for (a) C_2H_6 and (b) C_2H_4 in **HOF-ZSTU-5a** by using sorption isotherms at 273 K and 298 K.

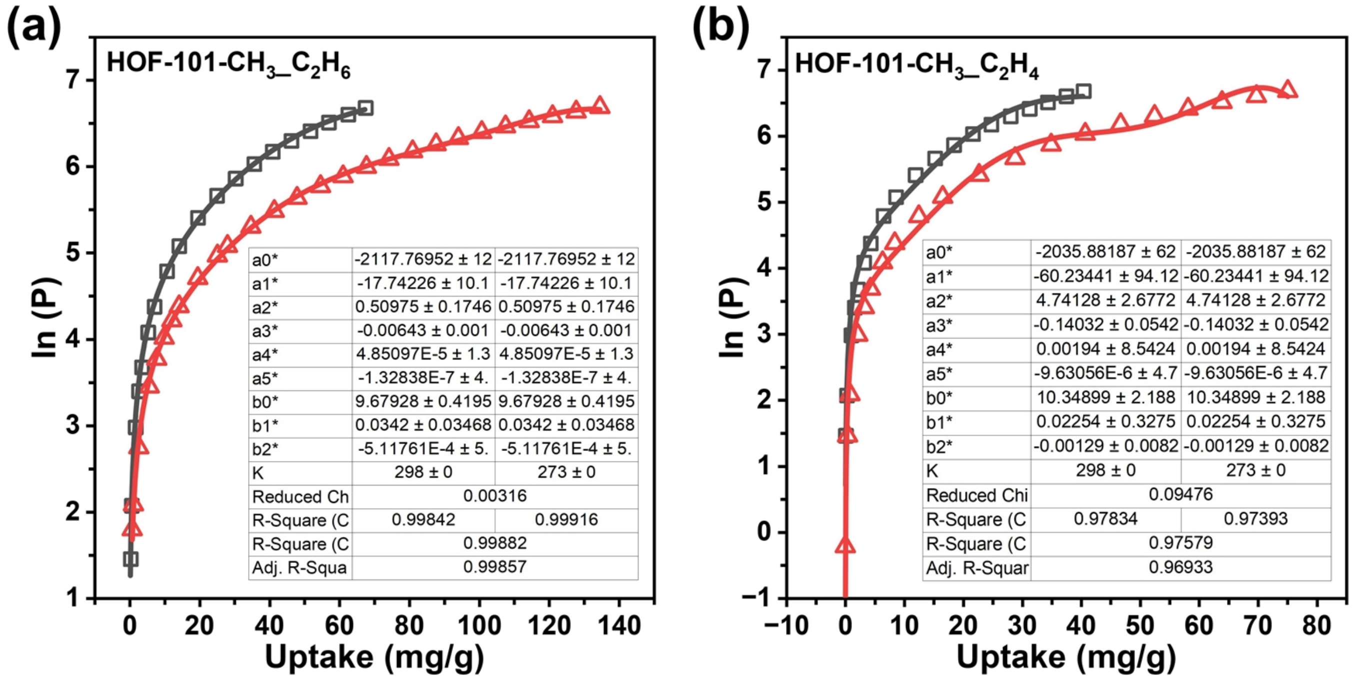

Fig. S26. The fitting results of Q_{st} for (a) C_2H_6 and (b) C_2H_4 in **HOF-101-CH$_3$** by using sorption isotherms at 273 K and 298 K.

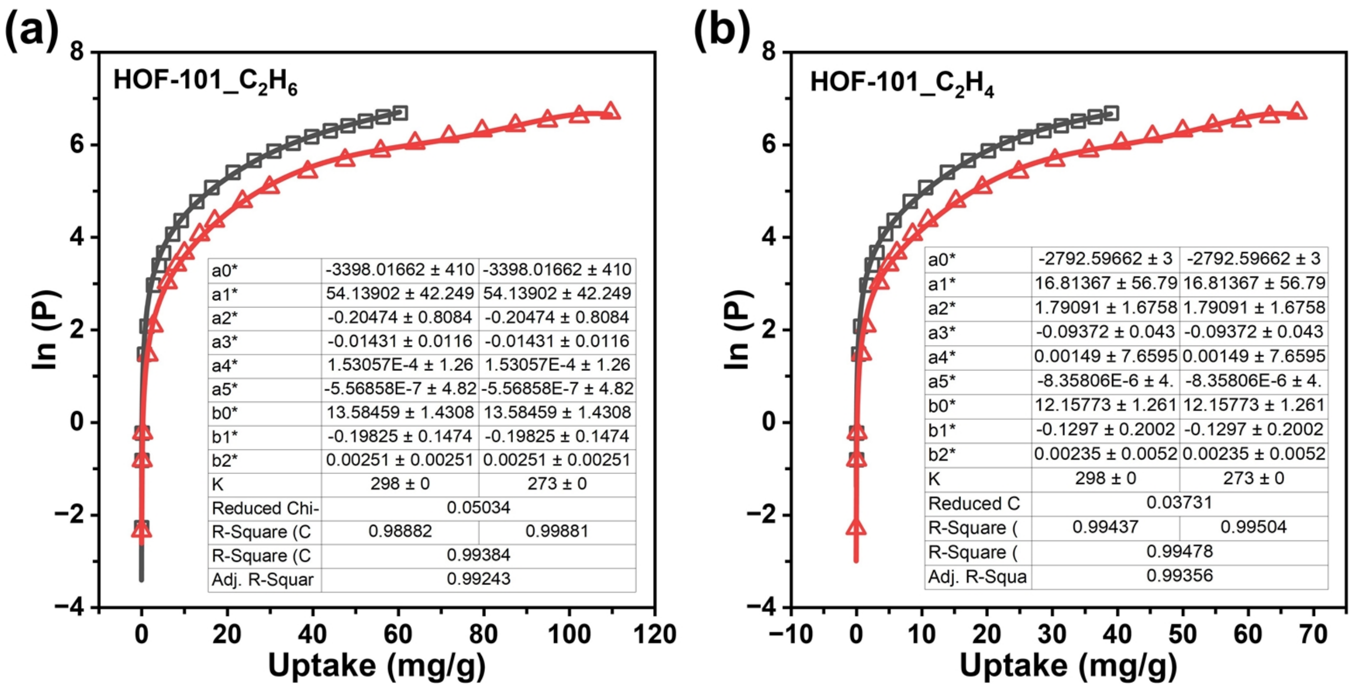

Fig. S27. The fitting results of Q_{st} for (a) C_2H_6 and (b) C_2H_4 in **HOF-101** by using sorption isotherms at 273 K and 298 K.

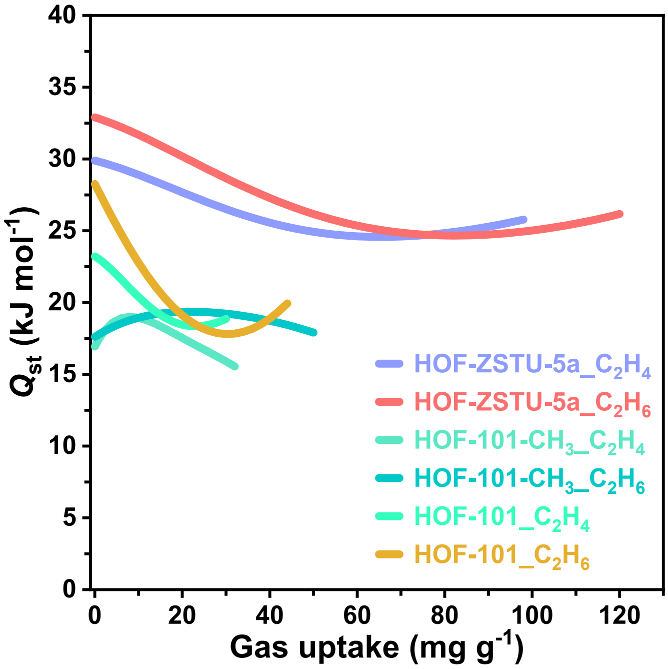

Fig. S28. Isosteric heat of adsorption (Q_{st}) plots for the adsorption of C_2H_6 and C_2H_4 by **HOF-101**, **HOF-101-CH$_3$**, and **HOF-ZSTU-5a**.

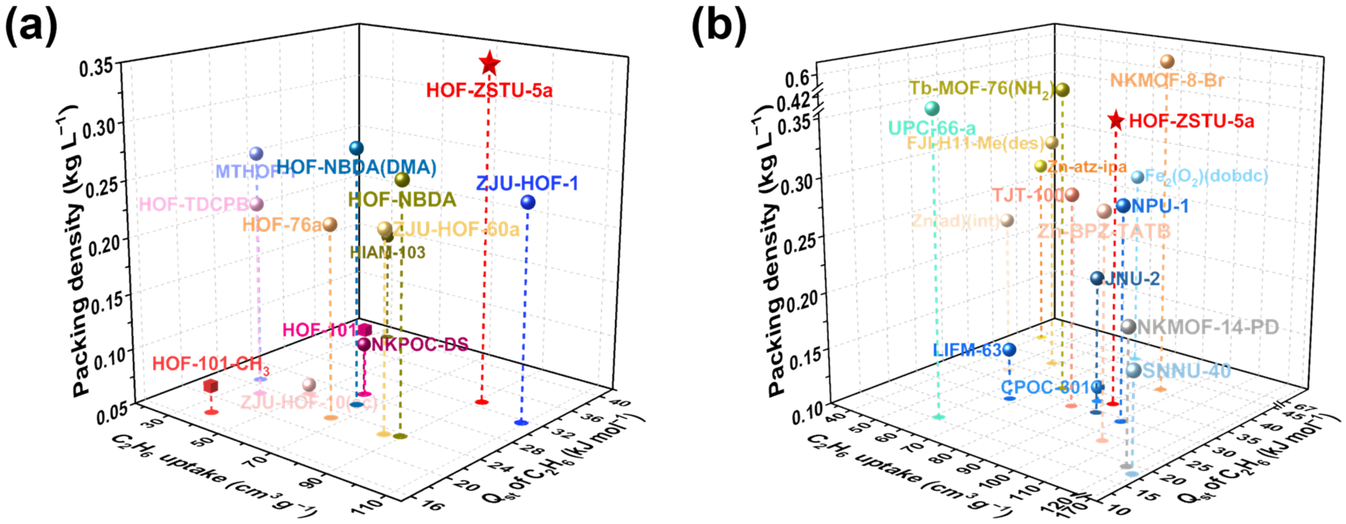

Fig. S29. The uptake, Q_{st} and packing density of C_2H_6 for **HOF-ZSTU-5a** and other C_2H_6-selective (a) HOFs and (b) MOFs were comprehensively compared.

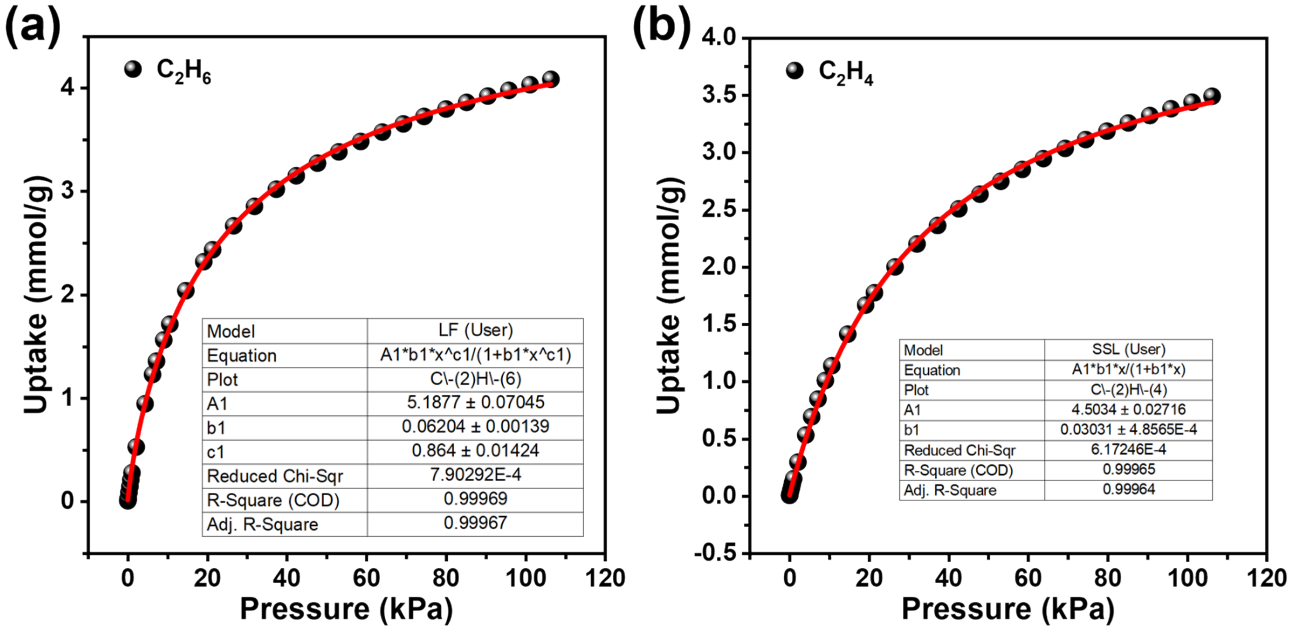

Fig. S30. IAST selectivity for single-component sorption isotherms of **HOF-ZSTU-5a** at 298 K. C_2H_6 was fitted based on single-site Langmuir-Freundlich (SSLF) equation and C_2H_4 was fitted based on single-site Langmuir (SSL) equation.

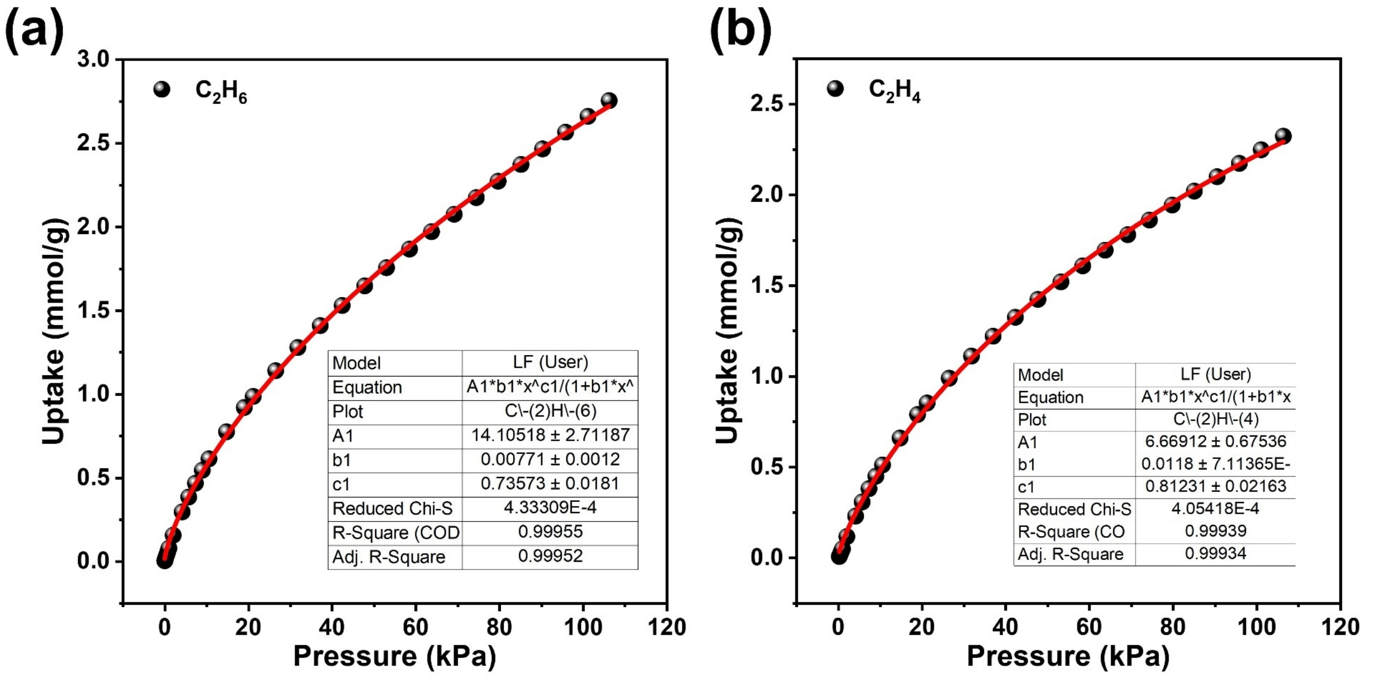

Fig. S31. IAST selectivity fitting based on SSLF equation for the single-component sorption isotherm of **HOF-101** at 298 K.

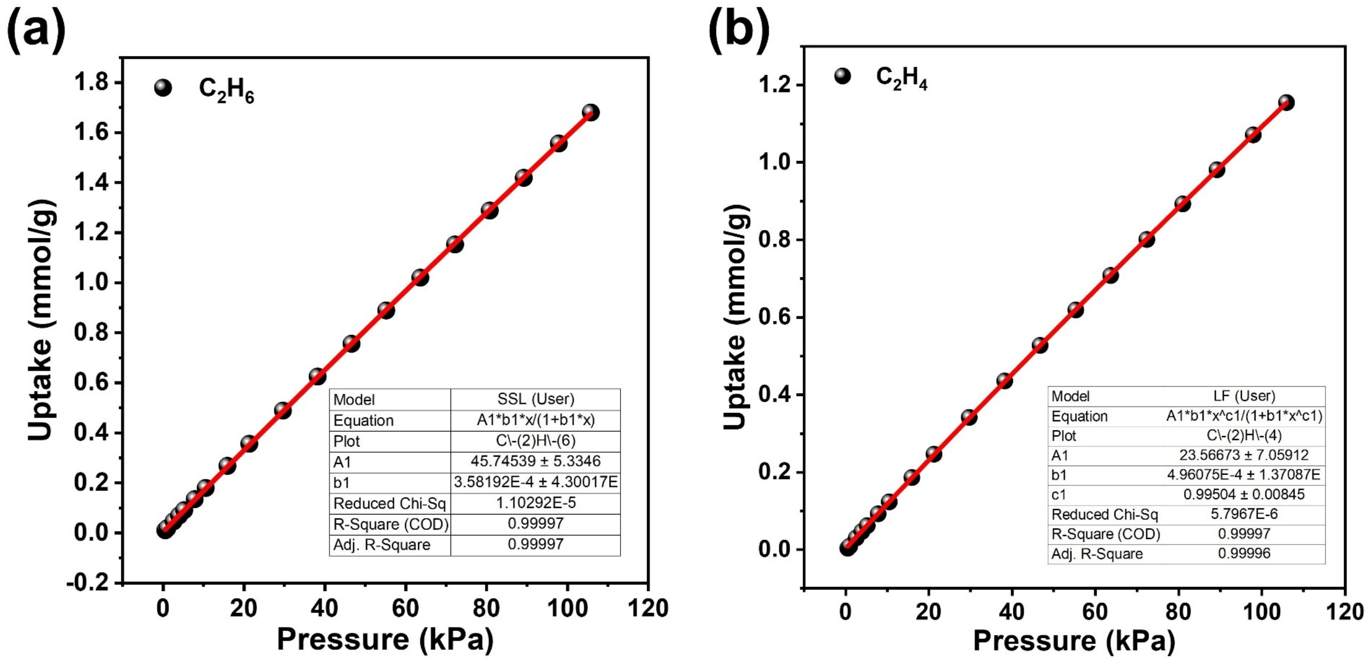

Fig. S32. IAST selectivity for single-component sorption isotherms of **HOF-101-CH$_3$** at 298 K. C_2H_6 was fitted based on SSL equation and C_2H_4 was fitted based on SSLF equation.

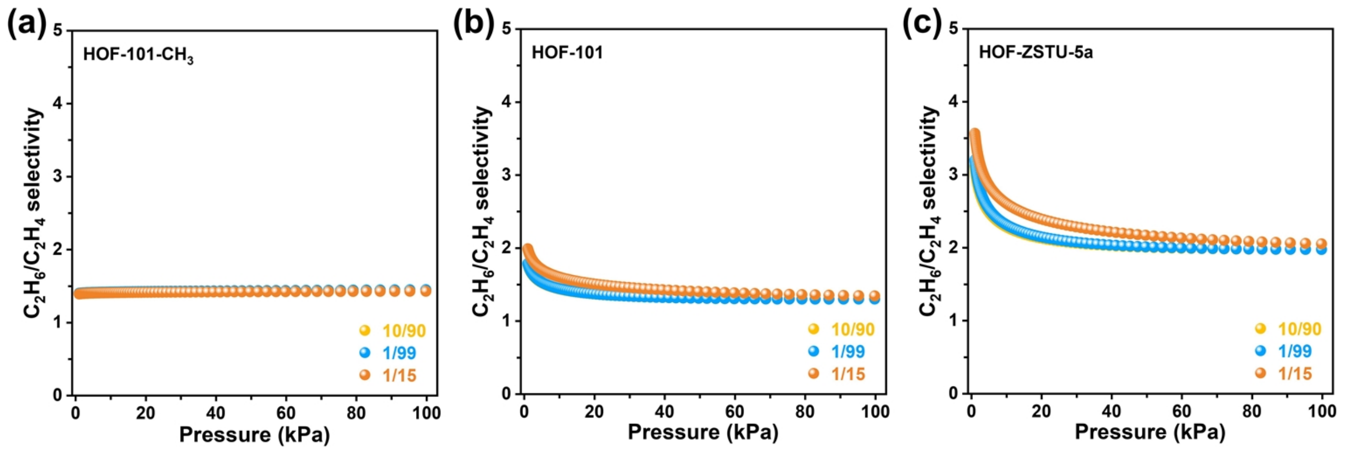

Fig. S33. IAST selectivity calculated for (a) **HOF-101-CH$_3$**, (b) **HOF-101**, and (c) **HOF-ZSTU-5**a at 298 K based on different C_2H_6/C_2H_4 mixture ratios.

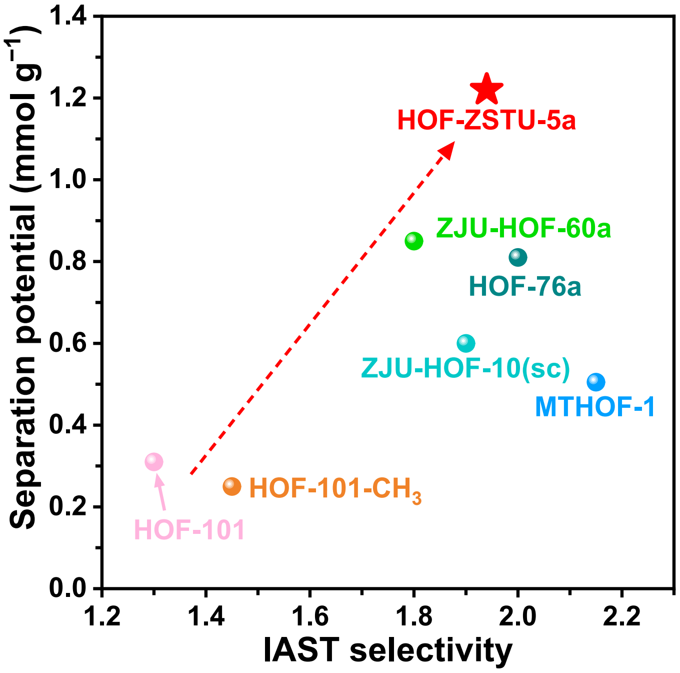

Fig. S34. Comparison of separation potential (Δq) and IAST selectivity of several HOFs at 298 K and 1 bar.

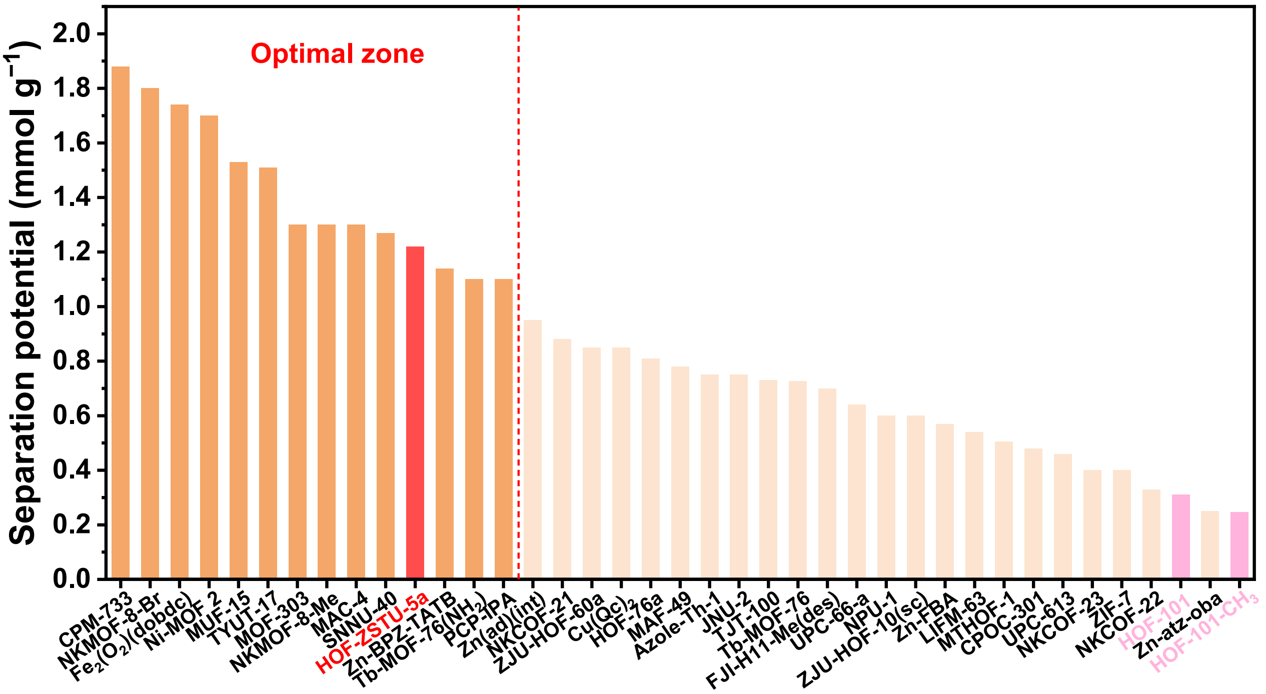

Fig. S35. The separation potential of the three HOFs in this paper and a number of C_2H_6-selective MOFs and HOFs were compared. Taking the interval of this value greater than 1 mmol g^{-1} as the optimal zone.

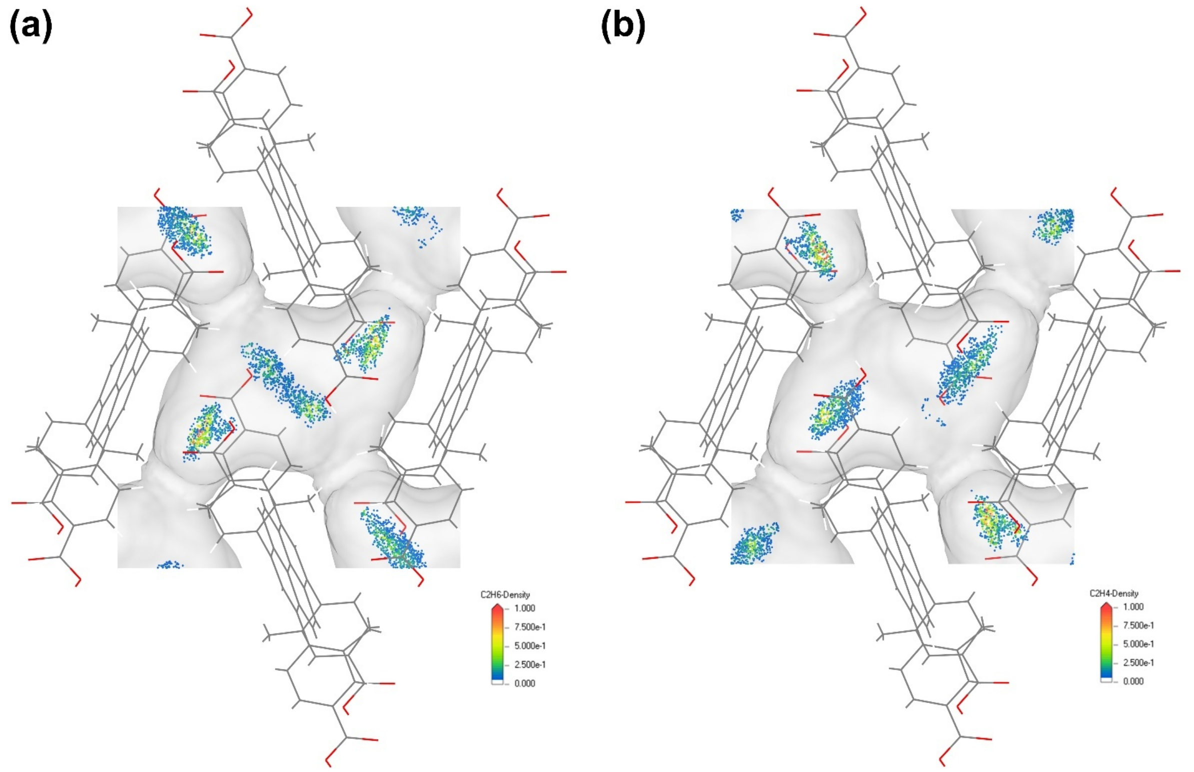

Fig. S36. Trajectory distribution of (a) C_2H_6 and (b) C_2H_4 in the **HOF-ZSTU-5a** crosslinked cavity at 298 K and 100 kPa. For comparison, the same trajectory density was used for both.

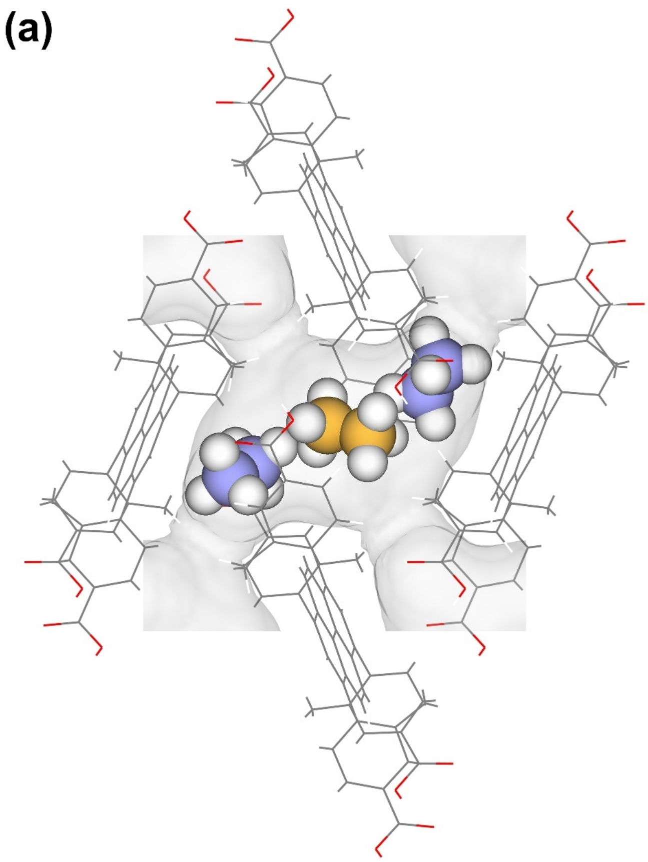

Fig. S37. Guest molecule positions of (a) C_2H_6 and (b) C_2H_4 visualized in the **HOF-ZSTU-5a** crosslinked cavity at 298 K and 100 kPa.

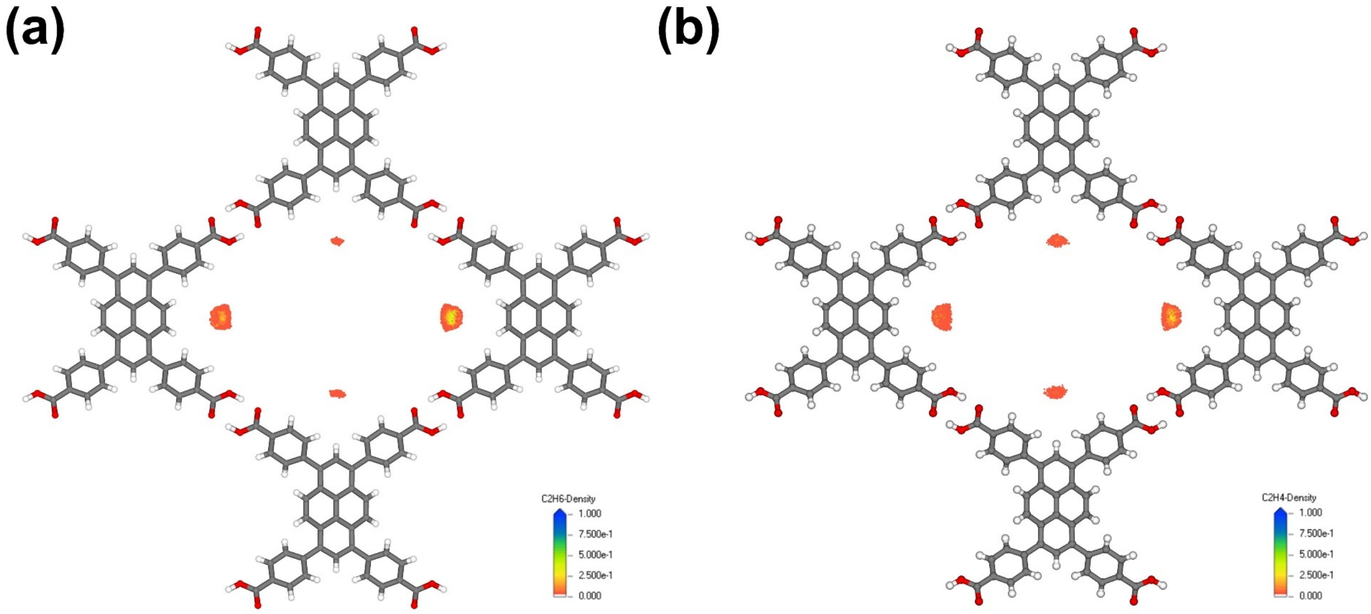

Fig. S38. Trajectory distribution of (a) C_2H_6 and (b) C_2H_4 in the **HOF-101** 1D channel at 298 K and 100 kPa. For comparison, the same trajectory density was used for both.

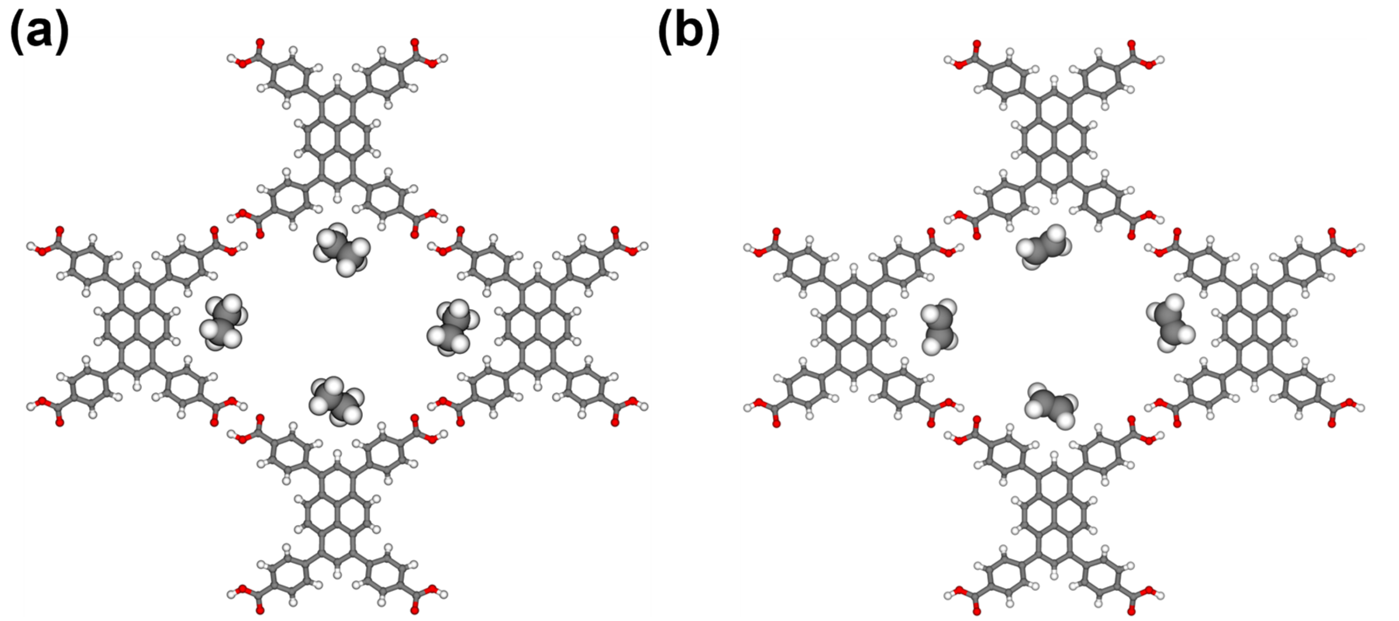

Fig. S39. Guest molecule positions of (a) C_2H_6 and (b) C_2H_4 visualized in the **HOF-101** 1D channel at 298 K and 100 kPa.

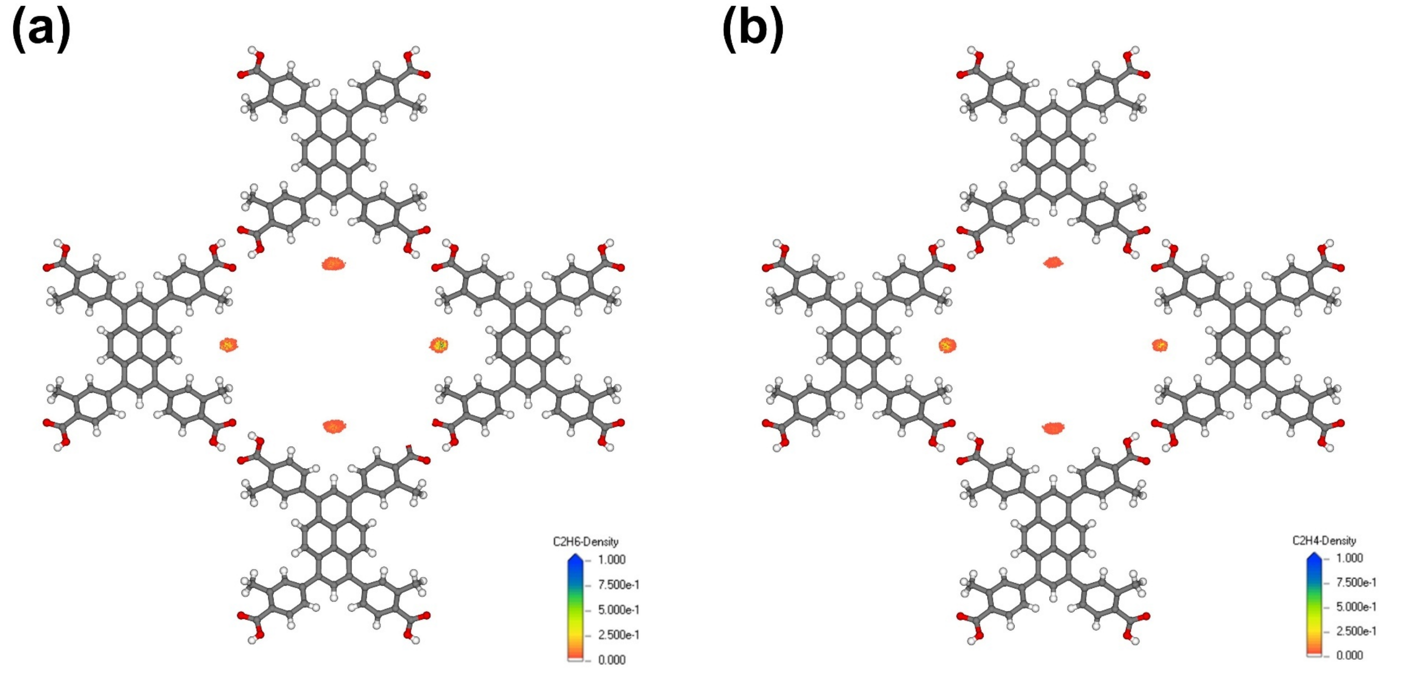

Fig. S40. Trajectory distribution of (a) C_2H_6 and (b) C_2H_4 in the **HOF-101-CH$_3$** 1D channel at 298 K and 100 kPa. For comparison, the same trajectory density was used for both.

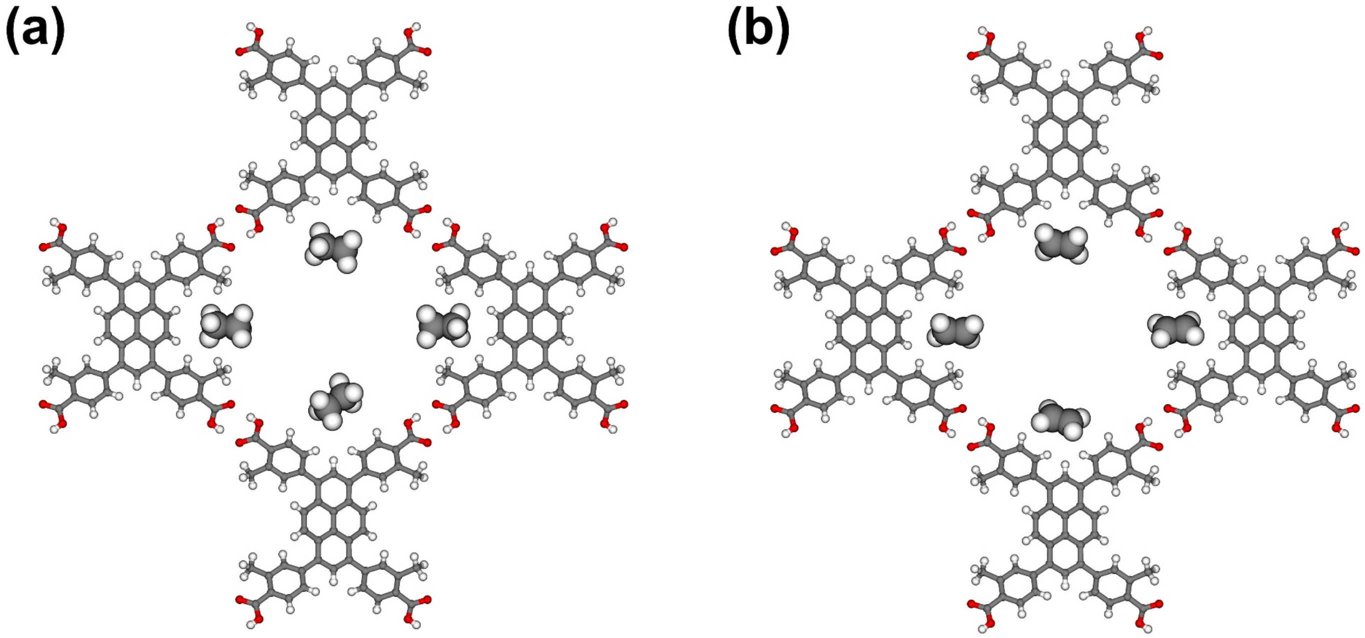

Fig. S41. Guest molecule positions of (a) C_2H_6 and (b) C_2H_4 visualized in the **HOF-101-CH$_3$** 1D channel at 298 K and 100 kPa.

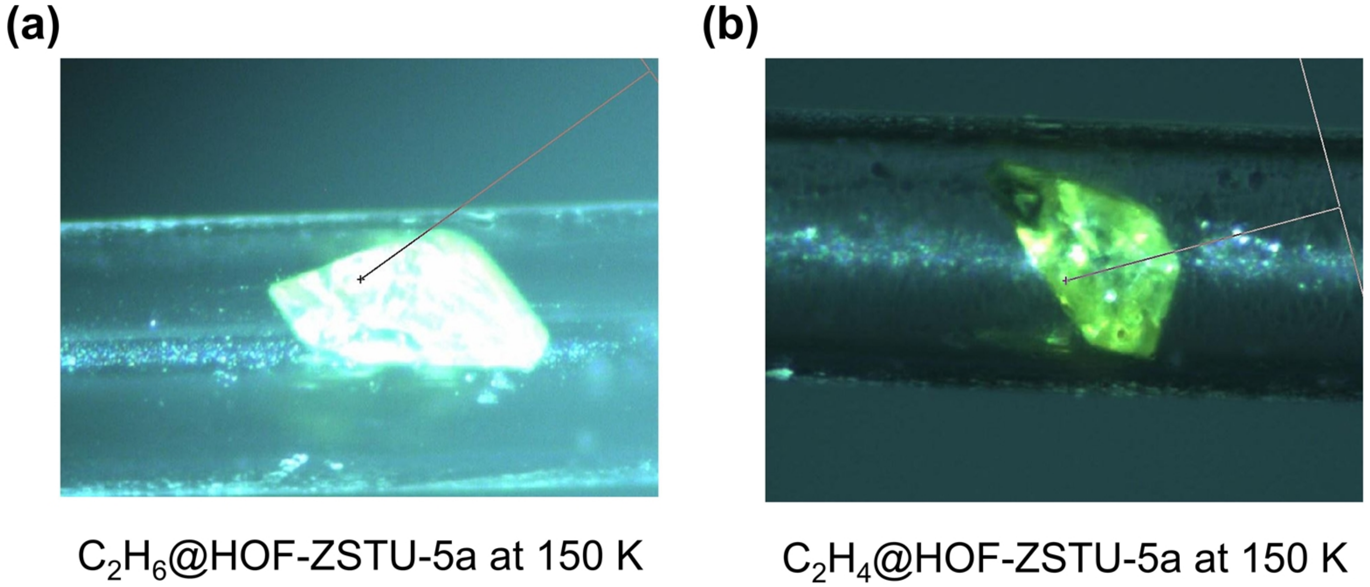

Fig. S42. Snapshots of **HOF-ZSTU-5a** crystals loaded with (a) C_2H_6 and (b) C_2H_4 at 150 K.

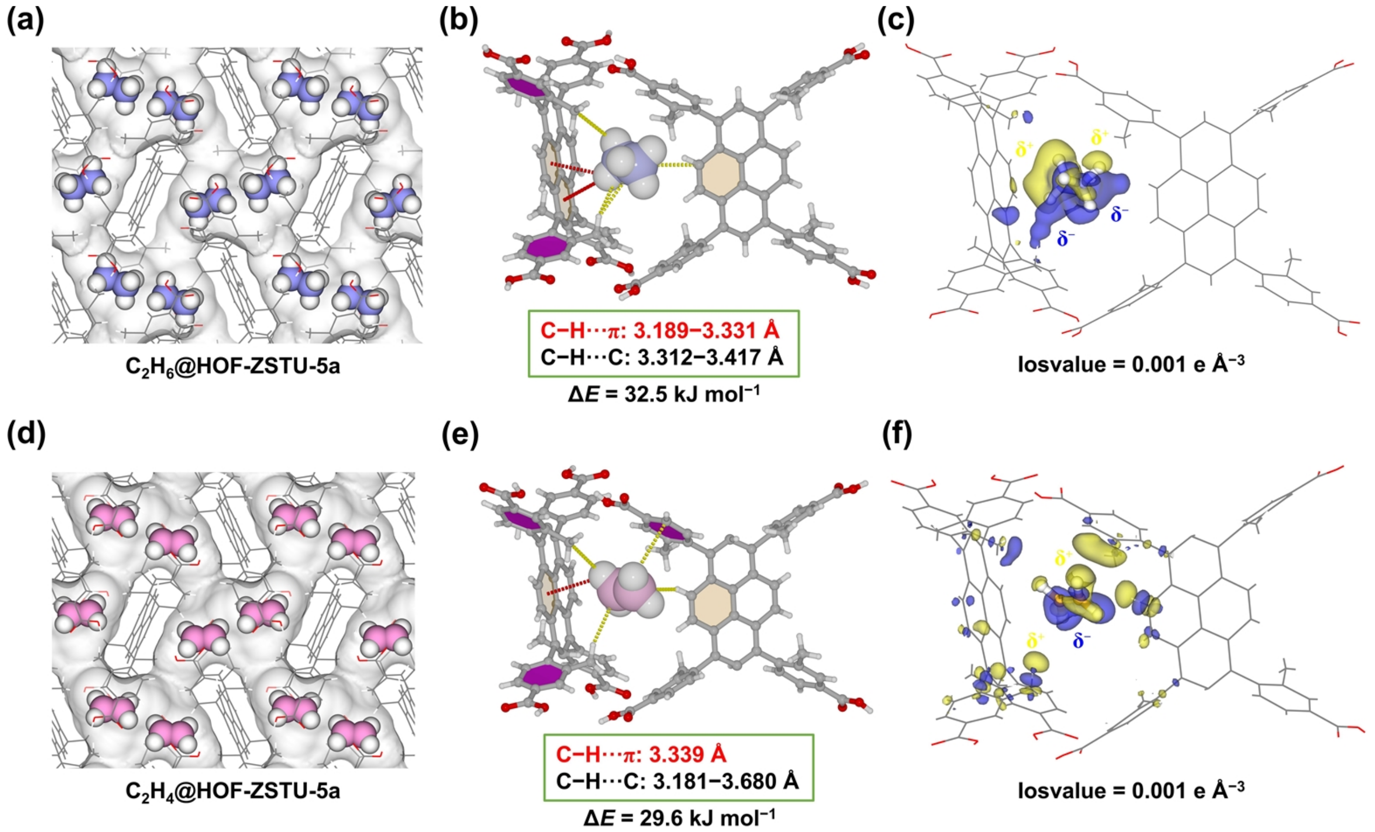

Fig. S43. **HOF-ZSTU-5a** crystal structure of in-situ loaded (a) C_2H_6 and (d) C_2H_4 at ambient temperature. The host−guest interaction forms between the framework and (b) site I C_2H_6, (e) C_2H_4 in the gas-loaded single crystal. Differential electron density map between the framework and (c) C_2H_6, (f) C_2H_4, where the blue region denotes charge aggregation and the yellow region denotes charge dissipation.

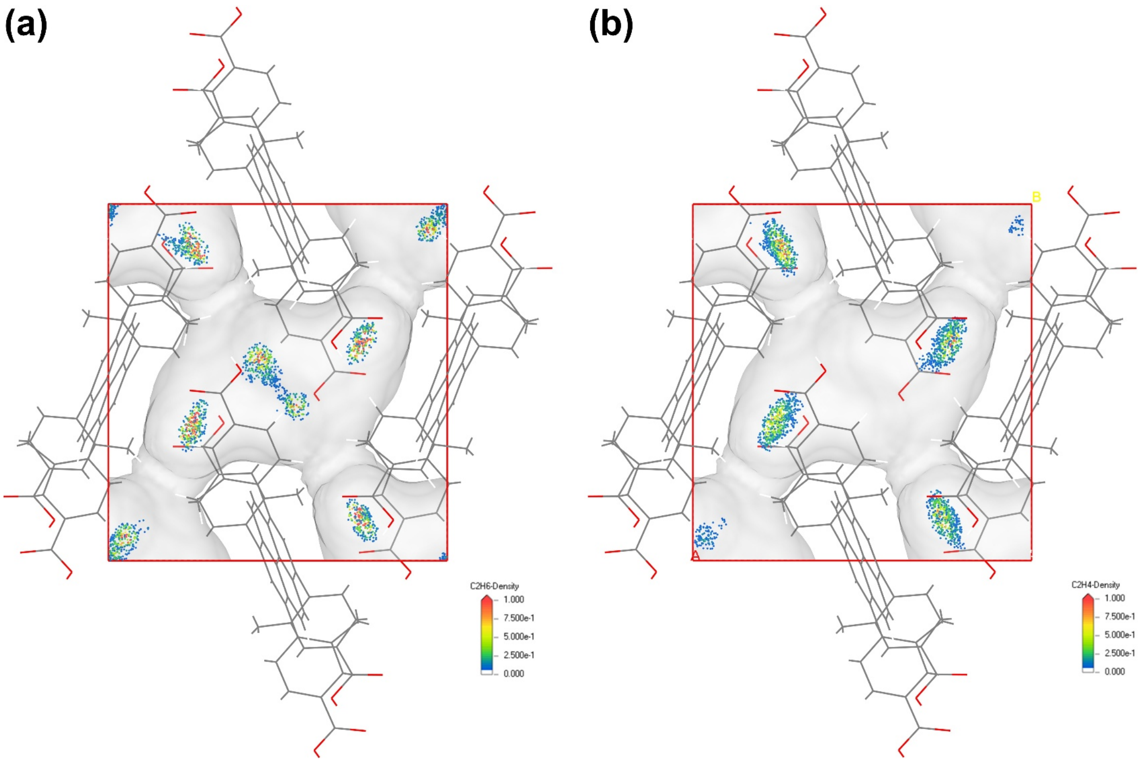

Fig. S44. Trajectory distribution of (a) C_2H_6 and (b) C_2H_4 in the **HOF-ZSTU-5a** crosslinked cavity at 150 K and 100 kPa. For comparison, the same trajectory density was used for both.

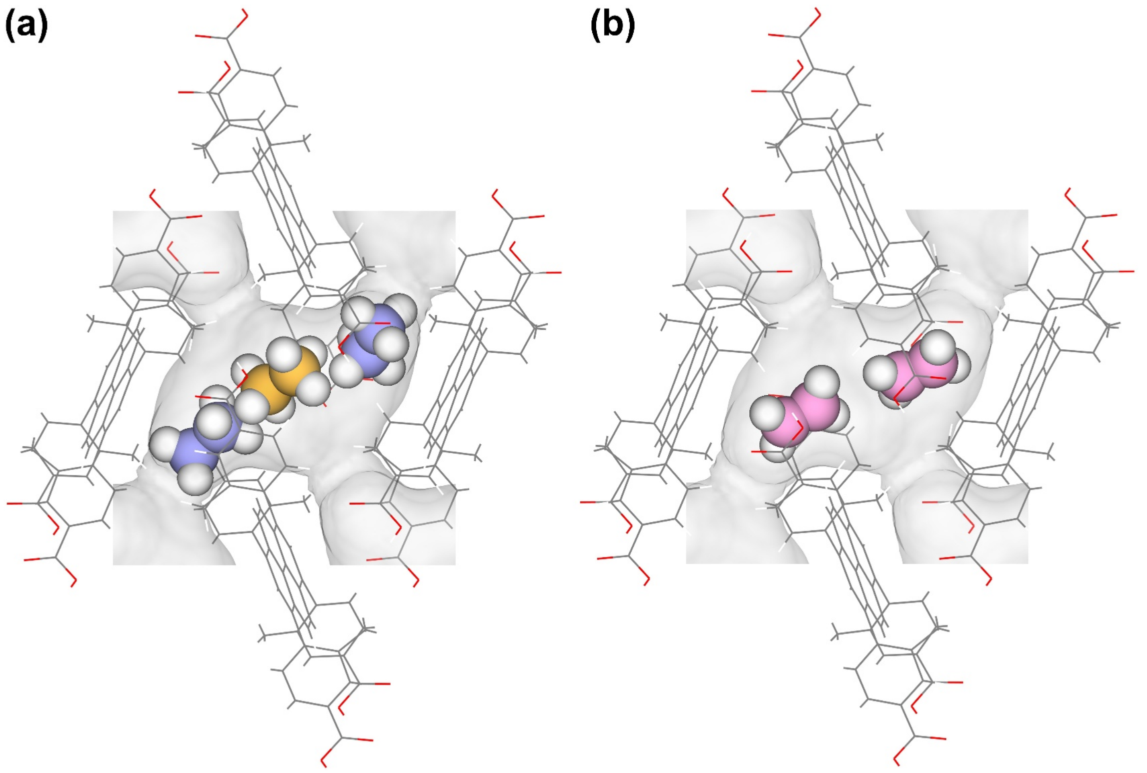

Fig. S45. Guest molecule positions of (a) C_2H_6 and (b) C_2H_4 visualized in the **HOF-ZSTU-5a** crosslinked cavity at 150 K and 100 kPa.

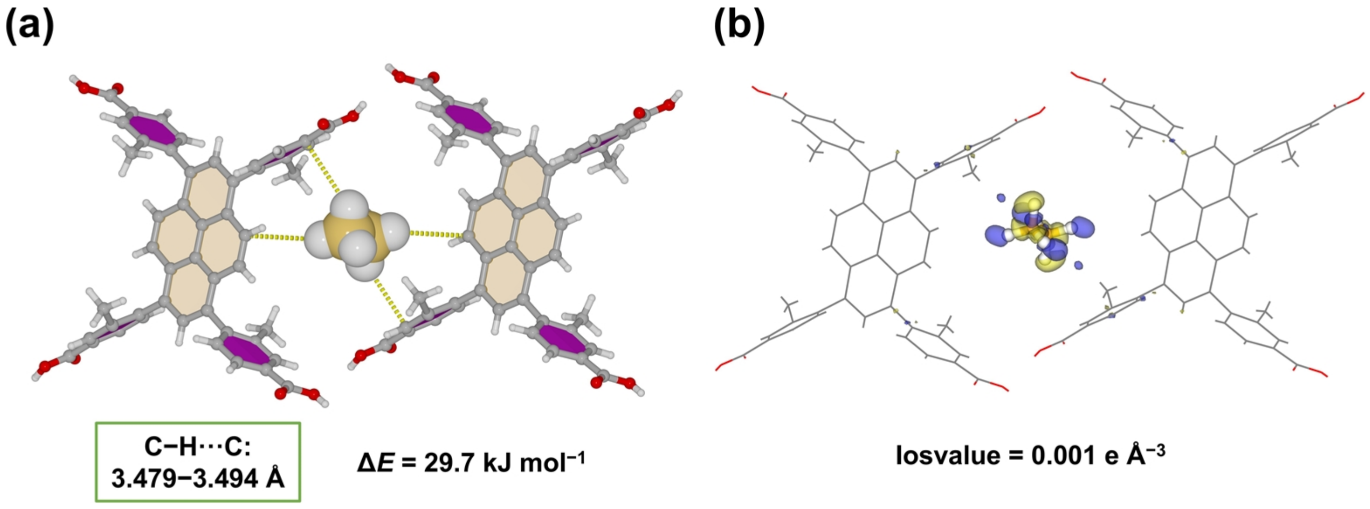

Fig. S46. (a) The host−guest interaction forms between the framework and site II C_2H_6 in the gas-loaded **HOF-ZSTU-5a** at 150 K. (b) Differential electron density map between the framework and site II C_2H_6 where the blue region denotes charge aggregation and the yellow region denotes charge dissipation. Notably, the host framework contributes only partially to the site II C_2H_6. In contrast, the two site I C_2H_6 molecules exhibit more pronounced guest−guest interactions with the site II C_2H_6 molecule sandwiched between them.

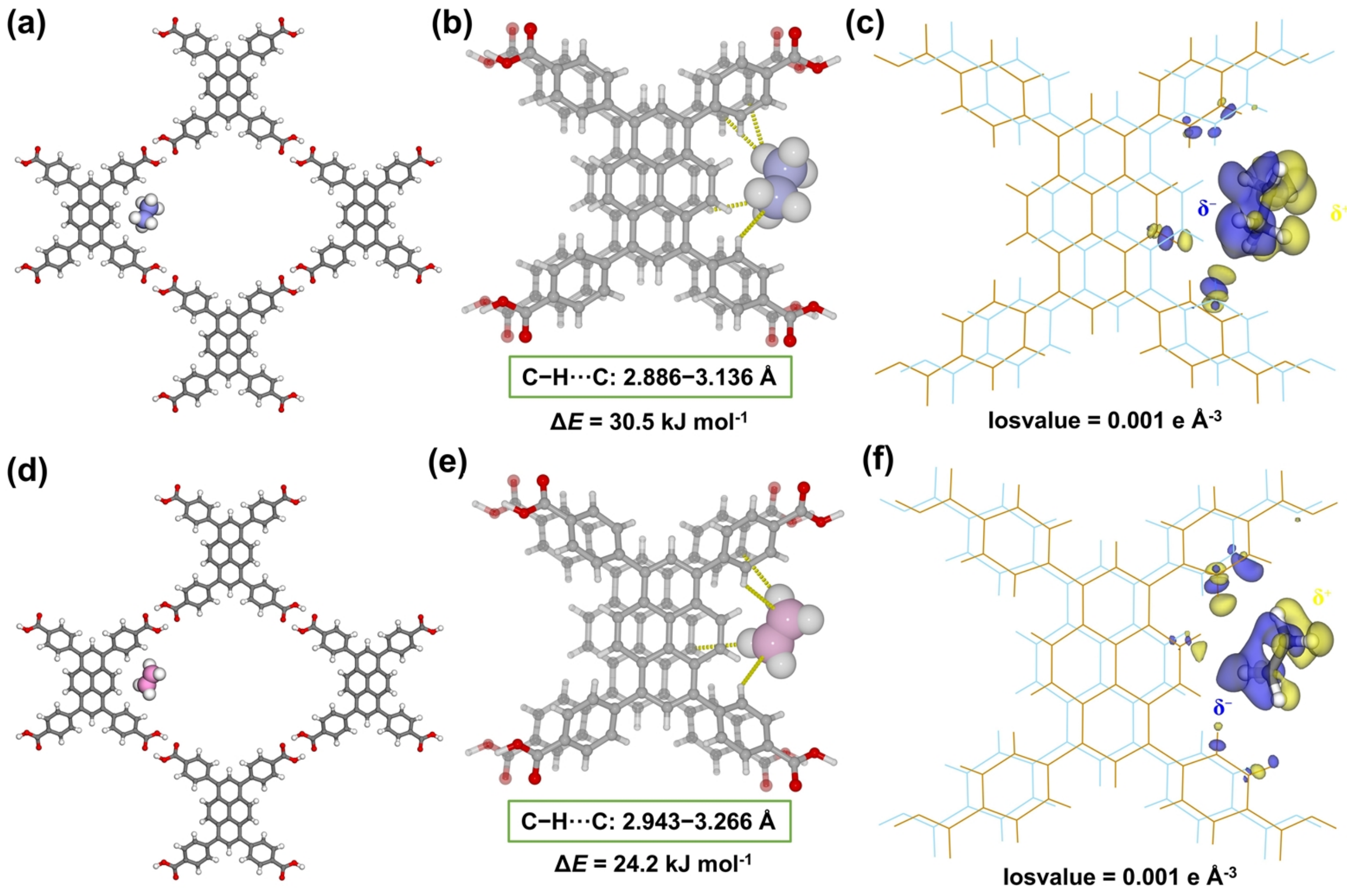

Fig. S47. Preferential binding sites for (a) C_2H_6 and (b) C_2H_4 in the **HOF-101** pore obtained using GCMC simulations. The host−guest interaction forms between the framework and (b) C_2H_6, (e) C_2H_4. Differential electron density map between the framework and (c) C_2H_6, (f) C_2H_4, where the blue region denotes charge aggregation and the yellow region denotes charge dissipation.

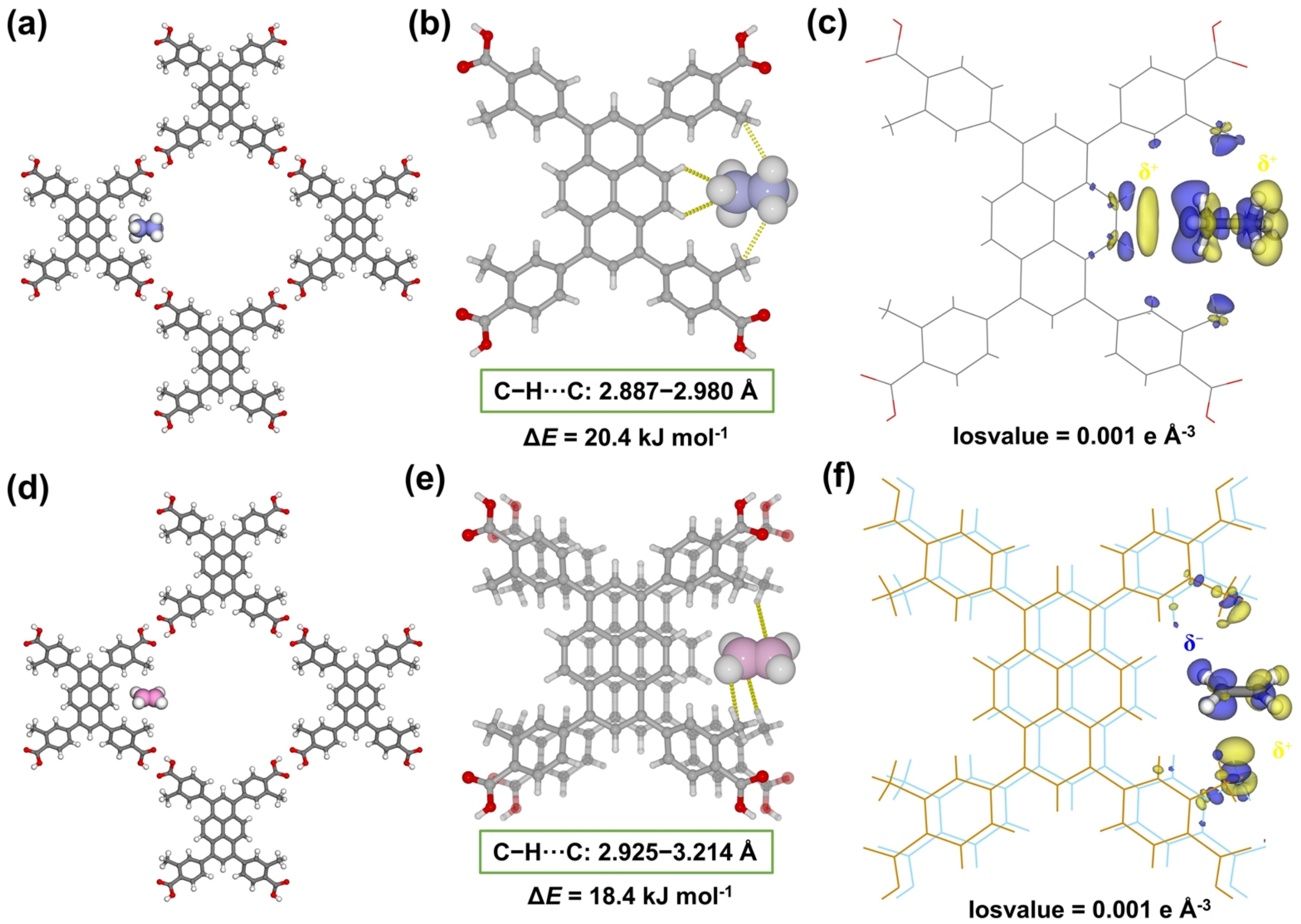

Fig. S48. Preferential binding sites for (a) C_2H_6 and (b) C_2H_4 in the **HOF-101-CH$_3$** pore obtained using GCMC simulations. The host−guest interaction forms between the framework and (b) C_2H_6, (e) C_2H_4. Differential electron density map between the framework and (c) C_2H_6, (f) C_2H_4, where the blue region denotes charge aggregation and the yellow region denotes charge dissipation.

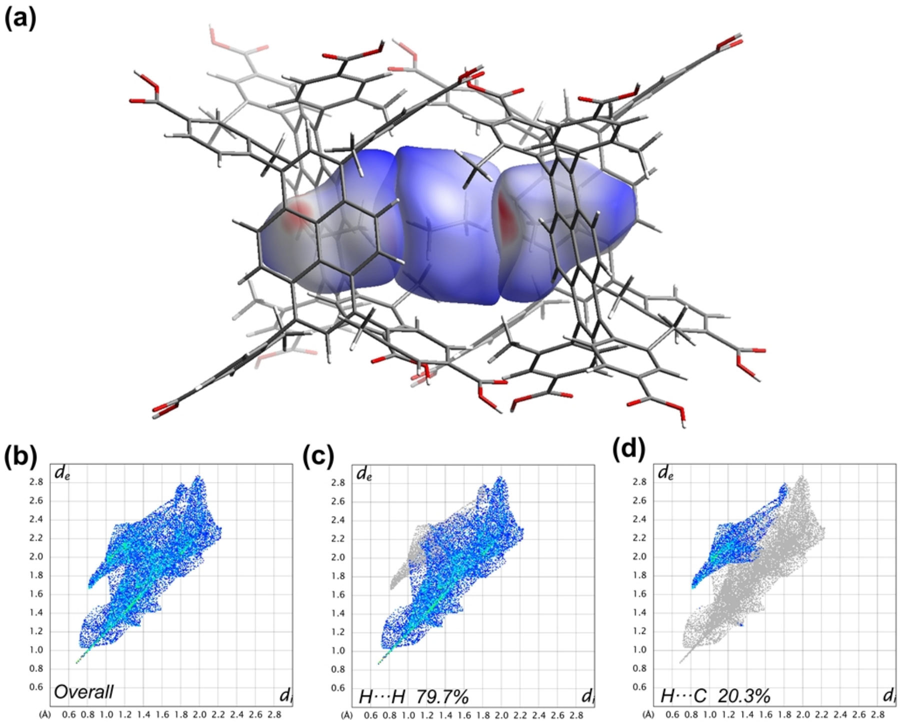

Fig. S49. (a) Hirshfeld surface analysis of **C_2H_6@HOF-ZSTU-5a** mapped with d_{norm}, highlighting strong intermolecular contacts in red (contact distance shorter than the sum of Van der Waals Radius). (b-d) 2D fingerprints of host−guest interactions.

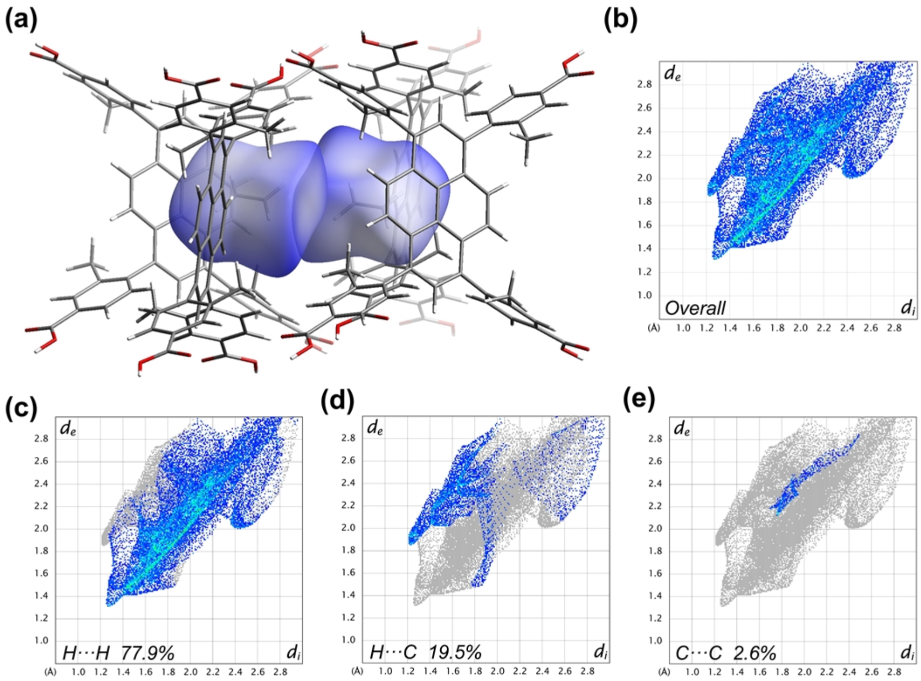

Fig. S50. (a) Hirshfeld surface analysis of **C_2H_4@HOF-ZSTU-5a** mapped with d_{norm}. (b-e) 2D fingerprints of host−guest interactions.

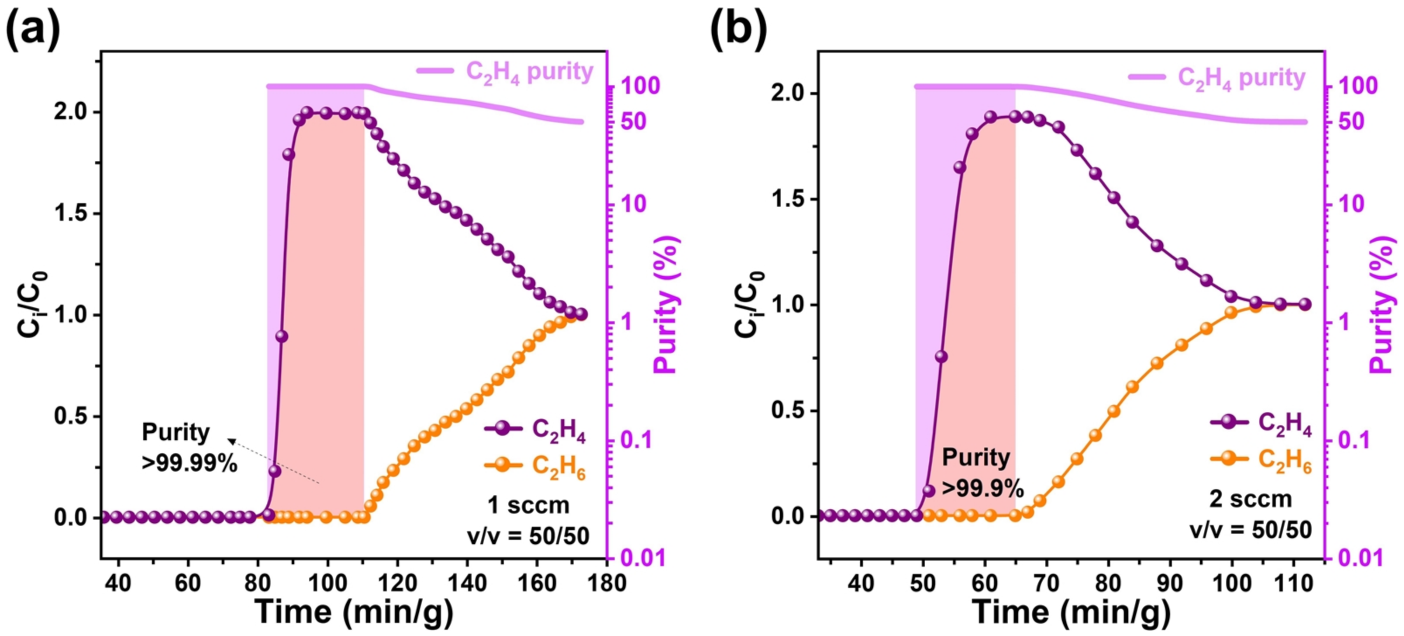

Fig. S51. The breakthrough tests of **HOF-ZSTU-5a** were performed at 298 K and 1 bar for equimolar C_2H_6/C_2H_4 mixtures with flow rates of (a) 1 mL min^{-1} and (b) 2 mL min^{-1} (use half the adsorbent mass).

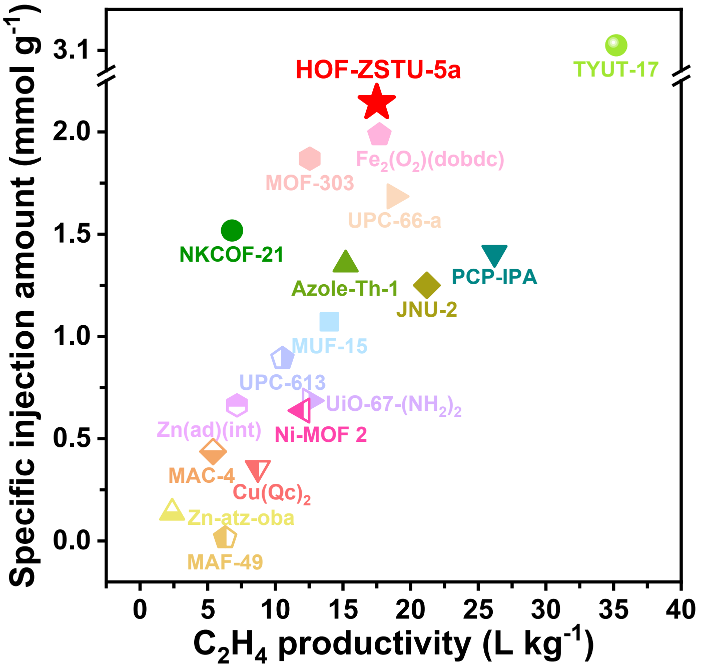

Fig. S52. Performance plots comparing the specific injection amount and C_2H_4 productivity of C_2H_6-selective MOFs under equimolar conditions.

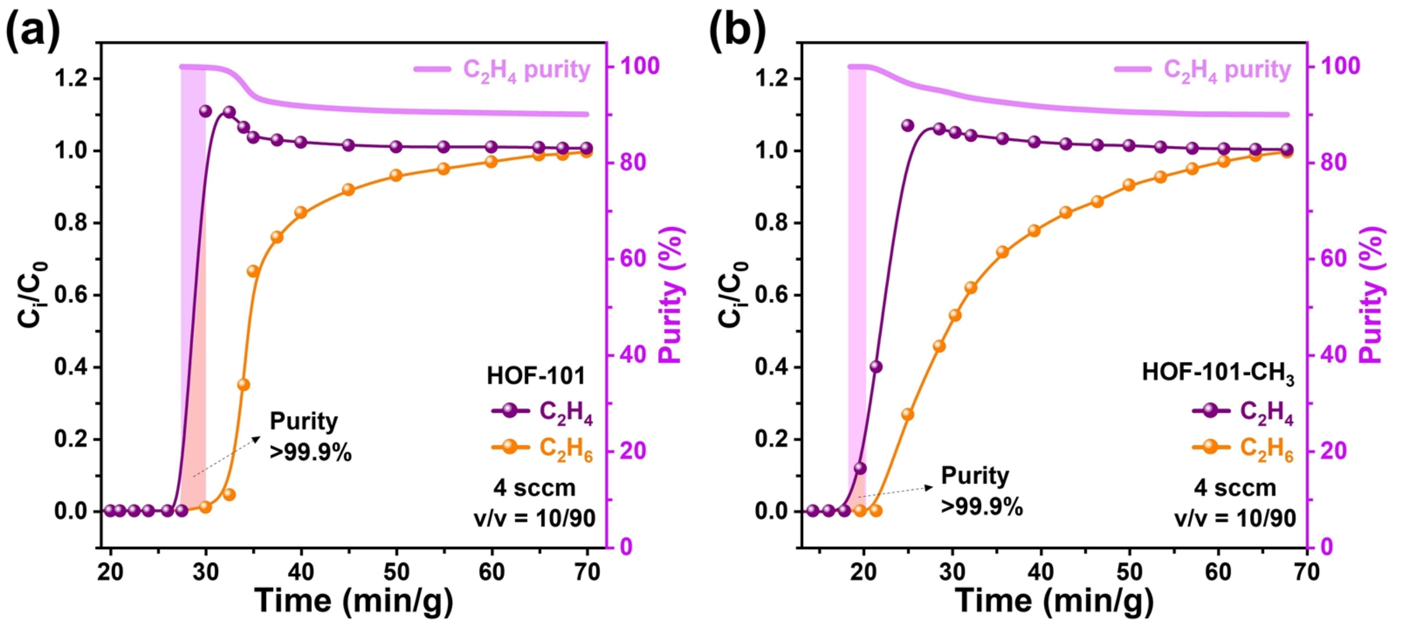

Fig. S53. The breakthrough tests of (a) **HOF-101** and (b) **HOF-101-CH$_3$** were performed at 298 K and 1 bar for 10/90 (v/v) C_2H_6/C_2H_4 mixtures with flow rates of 4 mL min^{-1}.

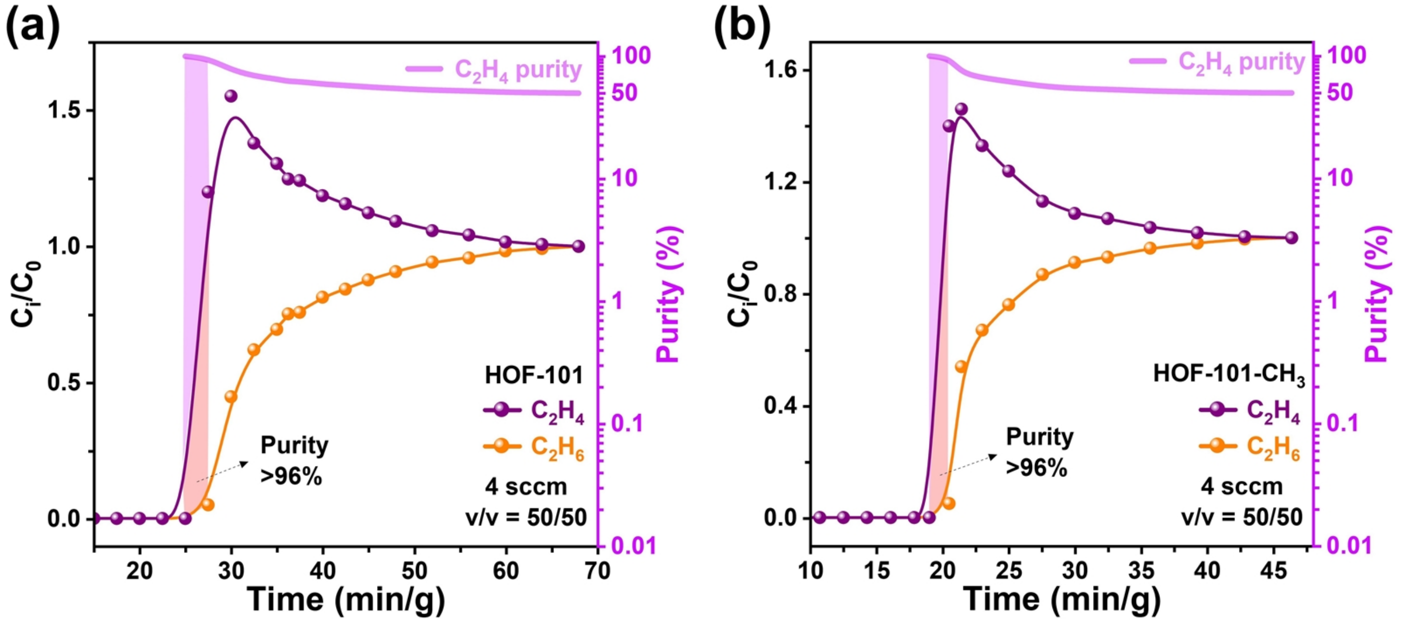

Fig. S54. The breakthrough tests of (a) **HOF-101** and (b) **HOF-101-CH$_3$** were performed at 298 K and 1 bar for equimolar C_2H_6/C_2H_4 mixtures with flow rates of 4 mL min^{-1}.

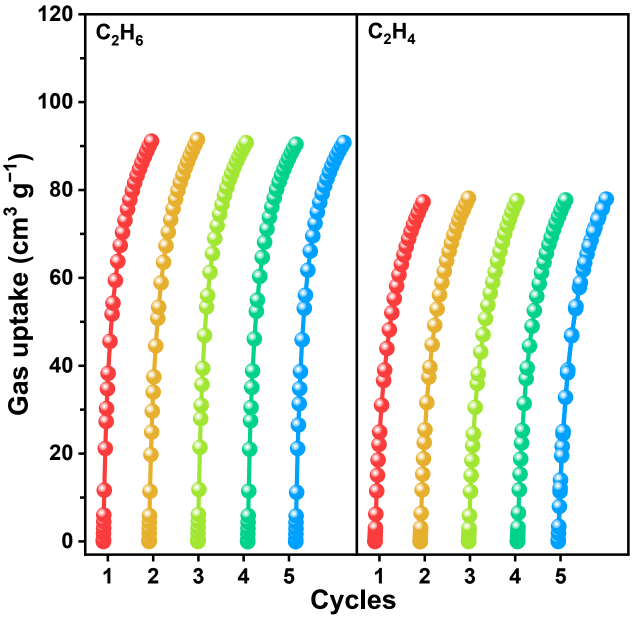

Fig. S55. Five consecutive single-component sorption isotherms of C_2H_6 and C_2H_4 for **HOF-ZSTU-5a** at 298 K.

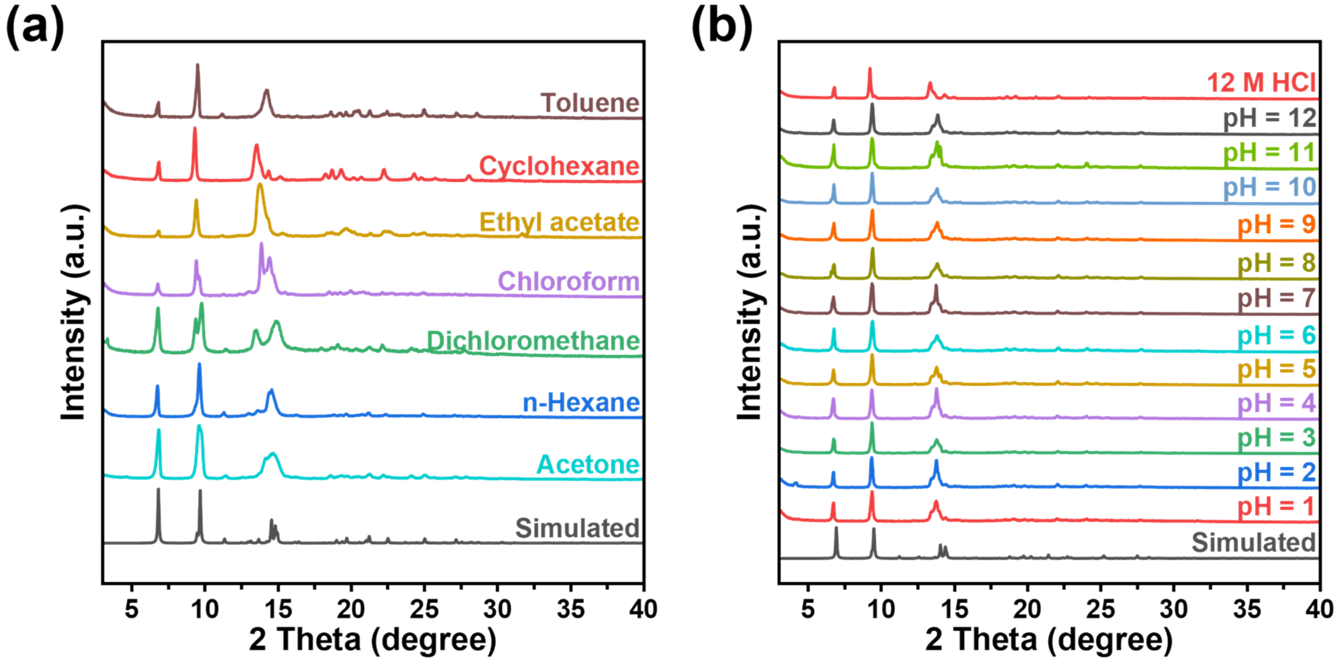

Fig. S56. PXRD plots of **HOF-ZSTU-5a** after (a) one week of immersion in different solvents and (b) one week of immersion in solutions of different pH.

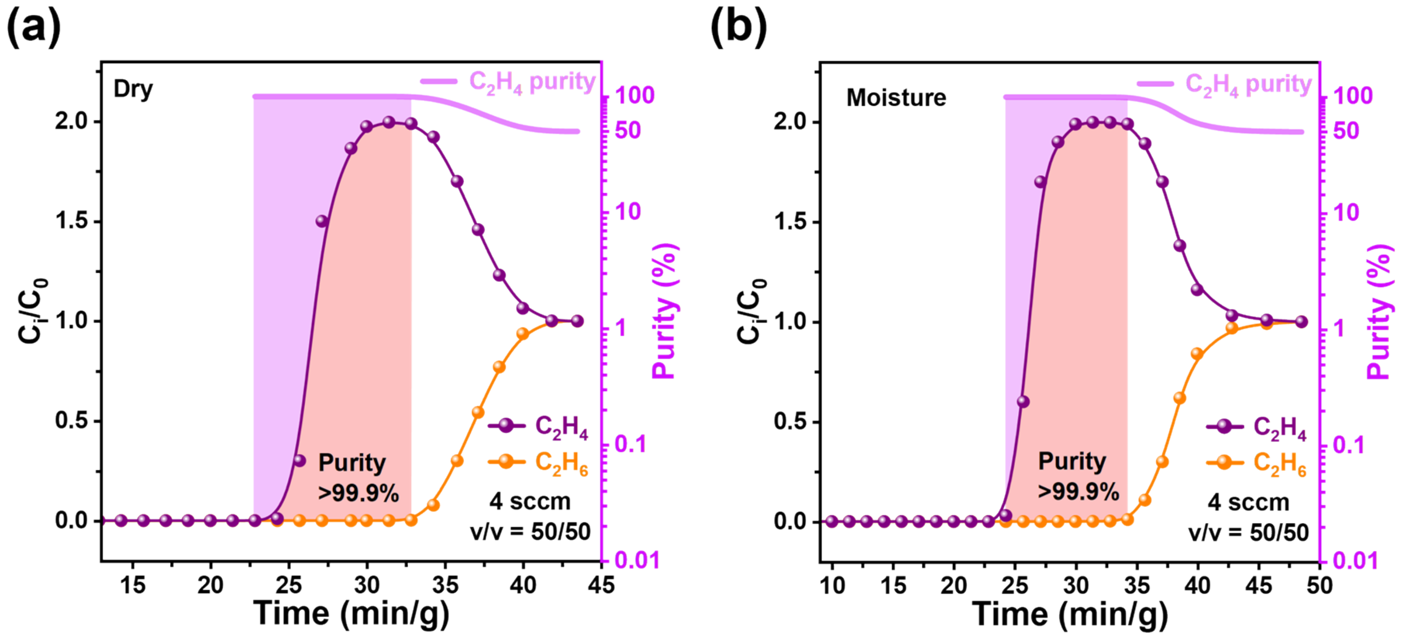

Fig. S57. The breakthrough tests of **HOF-ZSTU-5a** were performed at 298 K and 1 bar for equimolar C_2H_6/C_2H_4 mixtures with flow rates of 4 mL min^{-1}. (a) dry conditions. (b) At near-saturation humidity.

Table S1. The crystallographic data of **HOF-ZSTU-5** and **HOF-ZSTU-5a**.

**Crystal data**	**HOF-ZSTU-5**	**HOF-ZSTU-5a**
CCDC number	2425854	2425853
Crystal system	orthorhombic	orthorhombic
Space group	*Pbca* (61)	*Pbca* (61)
Molecular formula	$C_{60}H_{56}O_8$	$C_{50}H_{42}O_{10}$
Formula weight	905.04	802.83
a / Å	14.9347(7)	13.5872(7)
b / Å	14.1589(5)	14.1035(5)
c / Å	25.2455(8)	25.9503(10)
α/°	90	90
β/°	90	90
γ/°	90	90
Volume/ $Å^3$	5338.4(4)	4972.8(4)
Z	4	4
Z'	0.5	0.5
Density/ g cm^{-3}	1.129	1.072
GOF on F2	0.992	1.036
R1a[I>2σ(I)] (%)	14.77	10.25
ωR2b[I>2σ(I)] (%)	28.72	37.95

Table S2. The crystallographic data of **C_2H_6@HOF-ZSTU-5a** and **C_2H_4@HOF-ZSTU-5a** at ambient temperature.

**Crystal data**	**C_2H_6@HOF-ZSTU-5a**	**C_2H_4@HOF-ZSTU-5a**
CCDC number	2425858	2425857
Crystal system	orthorhombic	orthorhombic
Space group	*Pbca* (61)	*Pbca* (61)
Molecular formula	$C_{50}H_{40}O_8$	$C_{51}H_{34}O_8$
Formula weight	768.82	774.78
a / Å	13.444(3)	13.4346(13)
b / Å	14.142(3)	14.1142(9)
c / Å	25.861(5)	25.8466(17)
α/°	90	90
β/°	90	90
γ/°	90	90
Volume/ Å^3	4916.8(18)	4901.0(7)
Z	4	4
Z'	0.5	0.5
Density/ g cm^{-3}	1.039	1.050
GOF on F2	1.024	1.091
R1a[I>2σ(I)]	8.57	9.61
ωR2b[I>2σ(I)]	30.99	34.77

Table S3. The crystallographic data of **C_2H_6@HOF-ZSTU-5a** and **C_2H_4@HOF-ZSTU-5a** at 150 K.

**Crystal data**	**C_2H_6@HOF-ZSTU-5a**	**C_2H_4@HOF-ZSTU-5a**
CCDC number	2425856	2425855
Crystal system	orthorhombic	orthorhombic
Space group	*Pbca* (61)	*Pbca* (61)
Molecular formula	$C_{52}H_{46}O_8$	$C_{50}H_{34}O_8$
Formula weight	798.89	762.77
a / Å	13.4088(7)	13.4893(5)
b / Å	14.1154(5)	14.0799(4)
c / Å	25.8758(9)	25.9348(8)
α/°	90	90
β/°	90	90
γ/°	90	90
Volume/ Å^3	4897.5(4)	4925.7(3)
Z	4	4
Z'	0.5	0.5
Density/ g cm^{-3}	1.083	1.029
GOF on F2	1.092	1.019
R1a[I>2σ(I)]	9.78	7.90
ωR2b[I>2σ(I)]	35.45	29.18

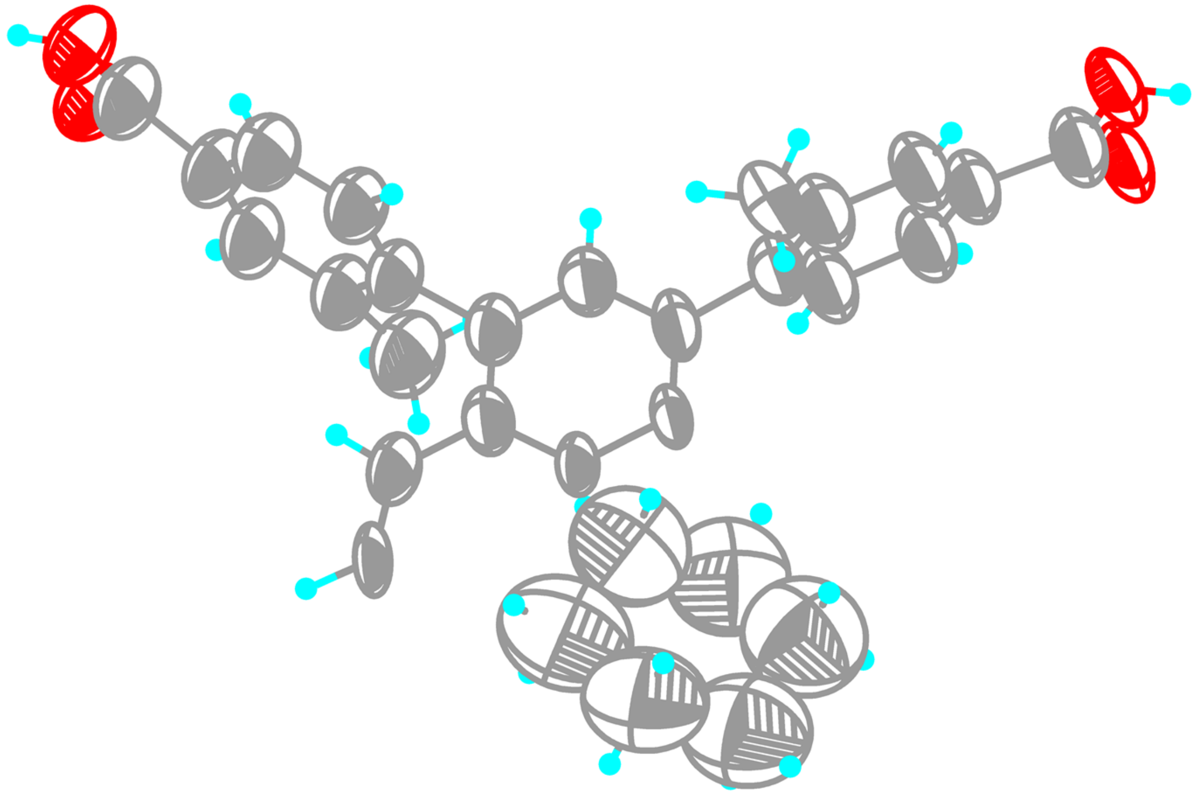

Fig. S58. ORTEP of **HOF-ZSTU-5** at 50% probability ellipsoid. Carbon: Gray, Oxygen: Red, Chloride: green, Hydrogen: Turquoise.

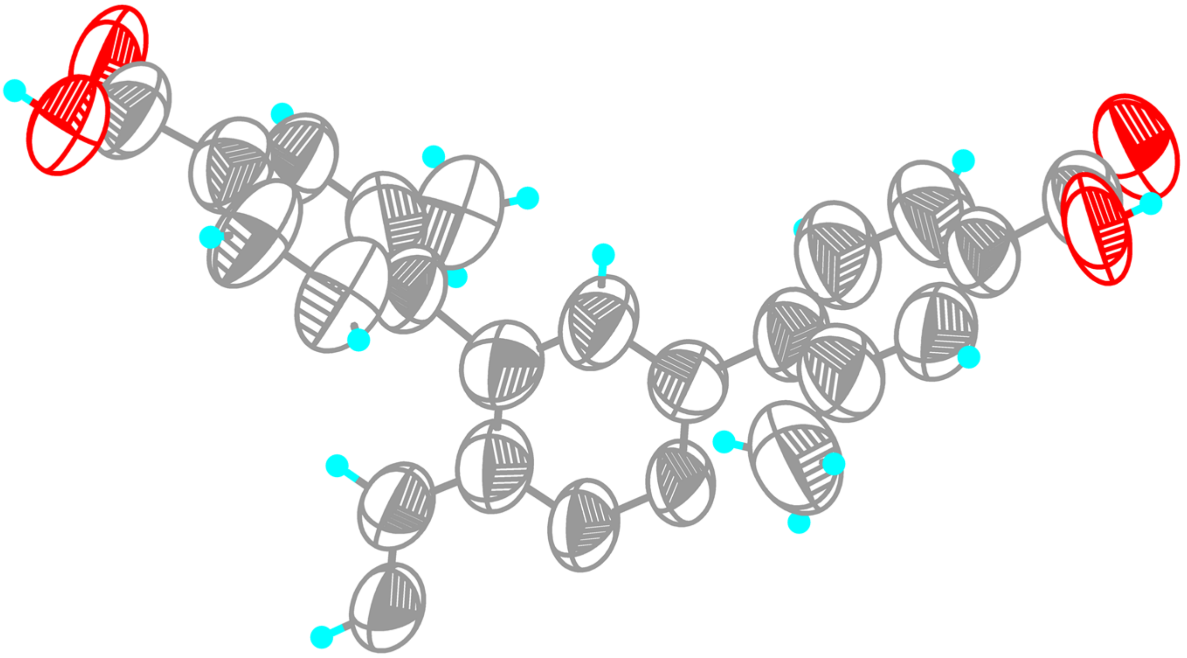

Fig. S59. ORTEP of **HOF-ZSTU-5a** at 50% probability ellipsoid. Carbon: Gray, Oxygen: Red, Chloride: green, Hydrogen: Turquoise.

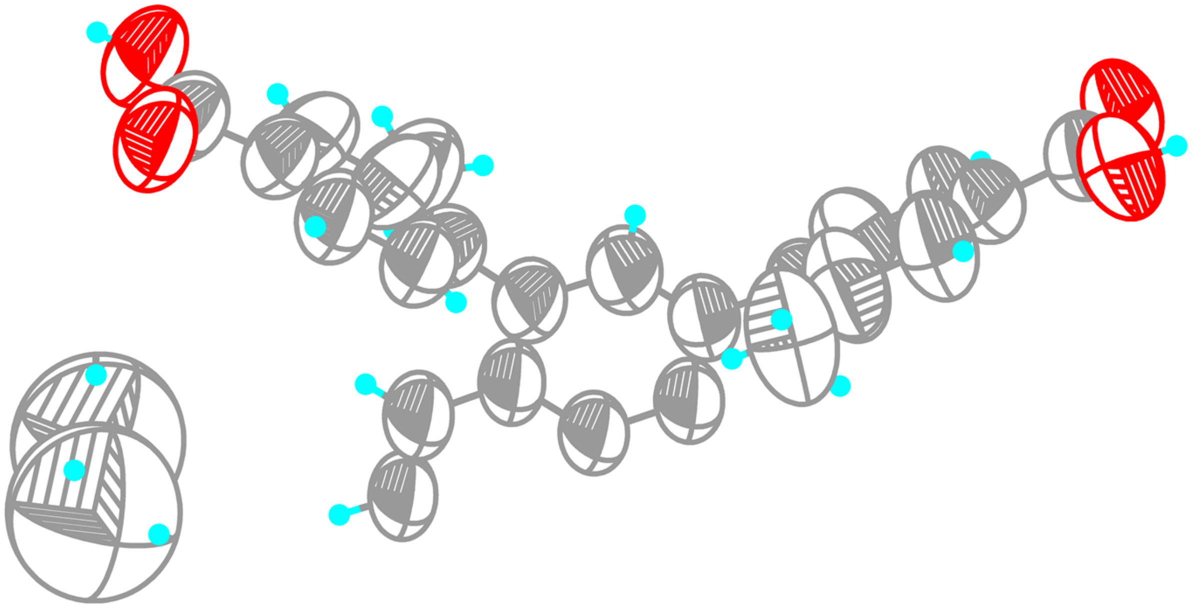

Fig. S60. ORTEP of **C_2H_6@HOF-ZSTU-5a (273 K)** at 50% probability ellipsoid. Carbon: Gray, Oxygen: Red, Chloride: green, Hydrogen: Turquoise.

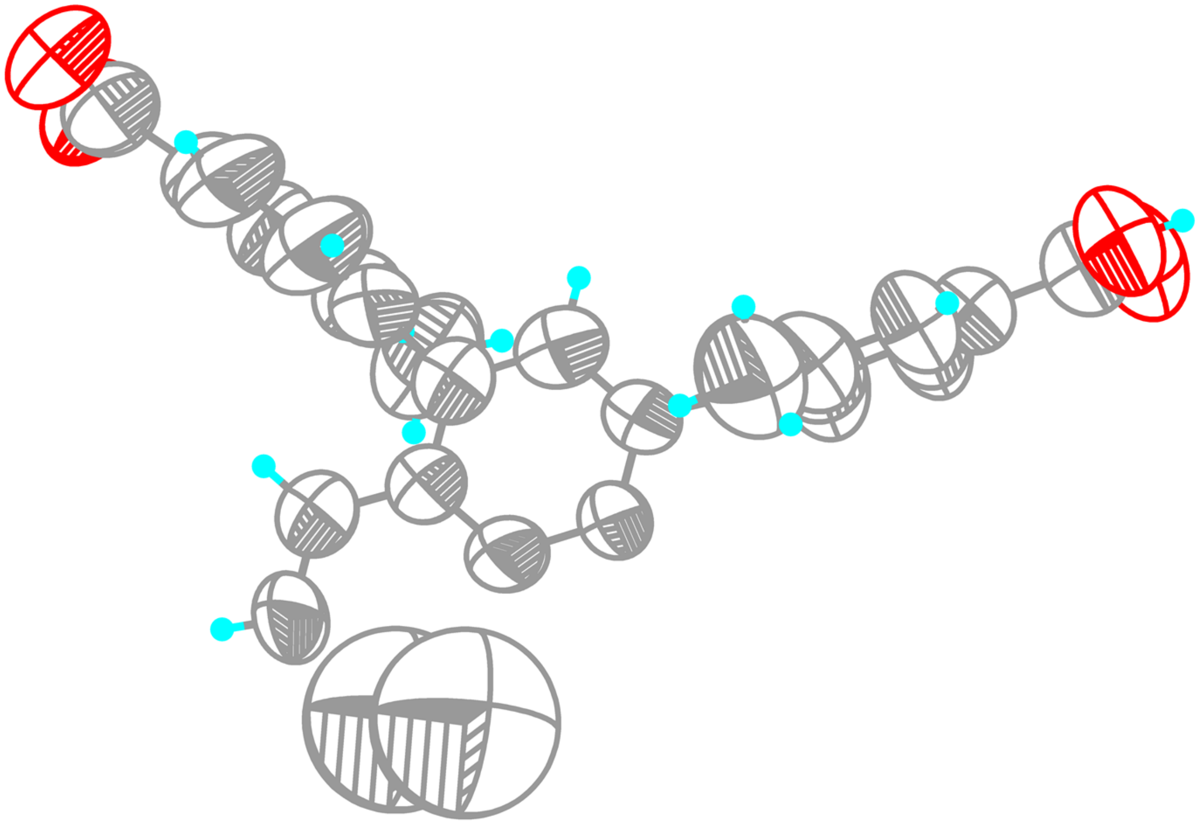

Fig. S61. ORTEP of **C_2H_4@HOF-ZSTU-5a (273 K)** at 50% probability ellipsoid. Carbon: Gray, Oxygen: Red, Chloride: green, Hydrogen: Turquoise.

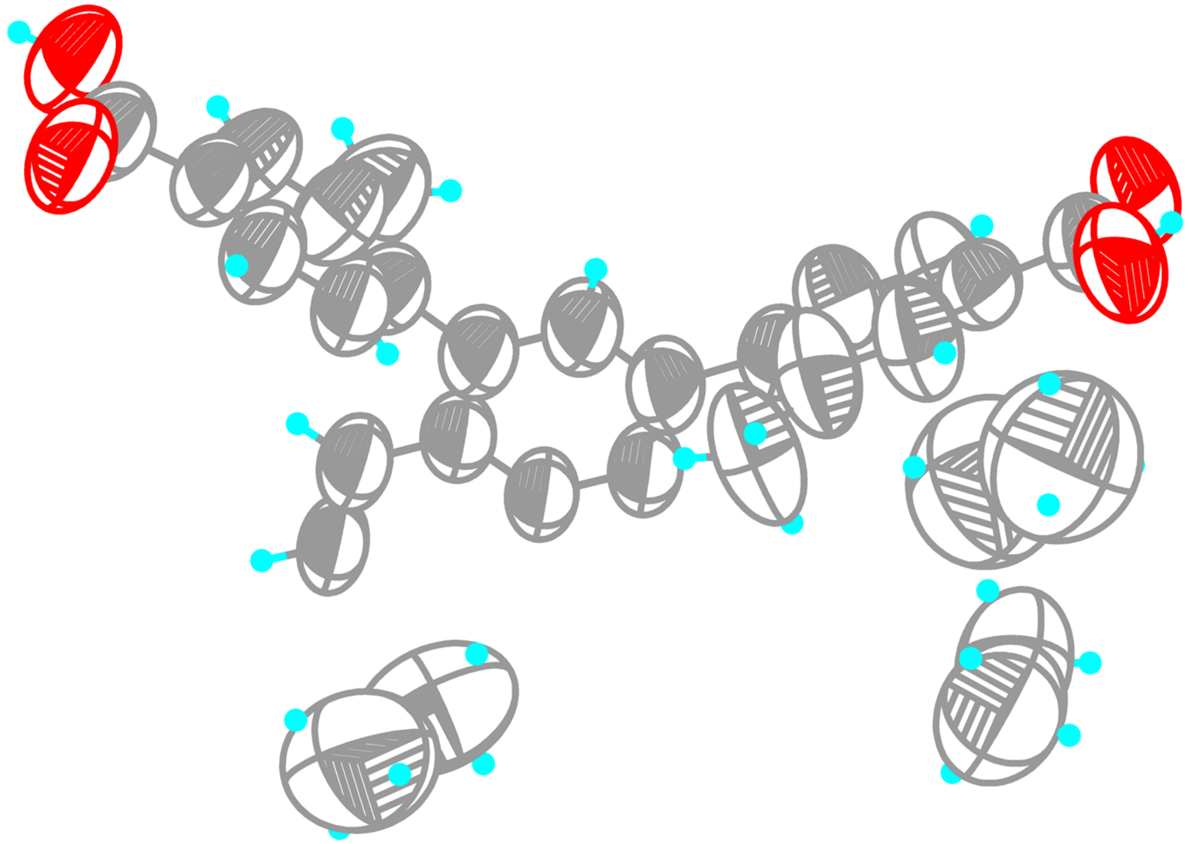

Fig. S62. ORTEP of **C_2H_6@HOF-ZSTU-5a (150 K)** at 50% probability ellipsoid. Carbon: Gray, Oxygen: Red, Chloride: green, Hydrogen: Turquoise.

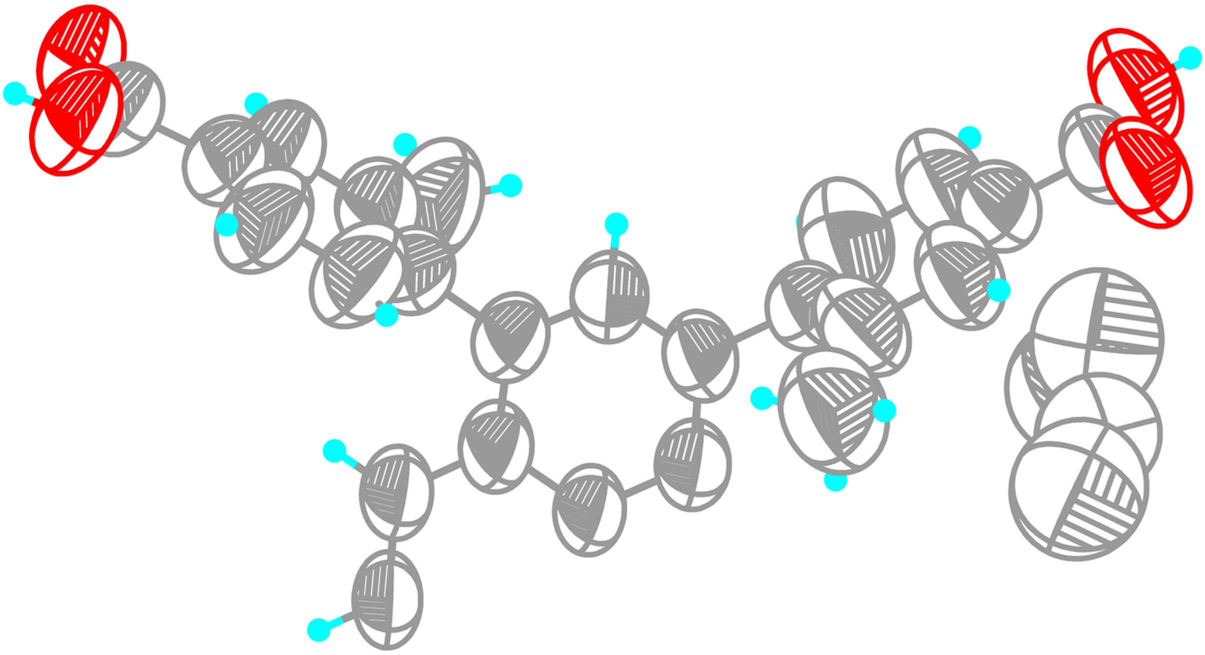

Fig. S63. ORTEP of **C_2H_4@HOF-ZSTU-5a (150 K)** at 50% probability ellipsoid. Carbon: Gray, Oxygen: Red, Chloride: green, Hydrogen: Turquoise.

Table S4. Water contact angles of hydrophobic porous materials with excellent separation properties.

**Materials**	**Water contact angle (°)**
HOF-ZSTU-5a	153
TDTBA-1[18]	131.3
MTHOF-1[19]	121.4
HOF-ZSTU-2a	113
MAF-42-*sp*[20]	141
FDMOF-2[21]	124.5
MAF-41[22]	153
ZnFPCP[23]	137.4
$Ag_2(o\text{-}Hmpba)_2(o\text{-}H_2mpba)_2$[24]	134.5
HOF-NKU-1[25]	87.8
SMS-POC-1[26]	150.4

Table S5. Water uptakes of C_2H_6-selective adsorbents at 60% and near-saturated RH (298 K).

Materials	60% RH ($g\ g^{-1}$)	Near-saturated RH ($g\ g^{-1}$)
HOF-101	0.088	0.773
HOF-101-CH_3	0.0209	0.5774
JNU-2	0.303	0.382
NKMOF-14-PZ	0.0228	0.335
NKMOF-14-PD	0.038	0.171
ZJU-HOF-1	0.0119	0.146
Zn(BDC)(H_2BPZ)[27]	0.067	0.102
SMS-POC-1	0.0118	0.0768 (Dynamic adsorption)
HOF-ZSTU-5a	0.0217	0.065

Table S6. Performance comparison of C_2H_6-selective HOFs and porous organic materials at room temperature.

Materials	C_2H_6 uptake ($cm^3 g^{-1}$)	C_2H_6 storage density ($kg L^{-1}$)	C_2H_6 Q_{st} ($kJ mol^{-1}$)	Δq ($mmol g^{-1}$)	Specific injection amount ($mmol g^{-1}$)	C_2H_4 productivity ($L kg^{-1}$)
HOF-ZSTU-5a	91.5	0.349	32.9	1.22	2.14	17.5
HOF-NBDA[28]	89.20	0.269	23.5	–	1.38[a]	29.2[a]
NKPOC-DS[29]	61.59	0.097	28.13	–	1.38	–
ZJU-HOF-60a[30]	84.90	0.227	23	0.85	1.12	7.8
ZJU-HOF-1[31]	109.00	0.243	31.5	1.78	1.00	21.9
HOF-TDCPB[32]	37.90	0.224	22.9	–	0.92	1
ZJU-HOF-10(sc)[33]	49.06	0.058	25.4	0.60	0.61	6.9
MTHOF-1	30.20	0.260	25.1	0.51	0.58[a]	8.7[a]
7b[34]	17.20	–	28.9	–	0.51	–
HOF-76a[35]	66.08	0.221	22.8	0.81	0.40	7.2
HIAM-103[36]	34.90	0.153	39.8	–	0.40[b]	4.35[b]
HIAM-102[37]	48.25	–	32.1	–	0.20	5.3[c]
HOF-NBDA(DMA)	65.20	0.279	26.1	–	0.28[a]	5.4[a]

Unless otherwise stated, the data used for the calculation of the Δq, Specific injection amount, and C_2H_4 productivity are those obtained with an equimolar mixture.

[a] The C_2H_6/C_2H_4 mixture ratio for the breakthrough test was 1/99 (v/v).

[b] The C_2H_6/C_2H_4 mixture ratio for the breakthrough test was 90/10 (v/v).

[c] The C_2H_6/C_2H_4 mixture ratio for the breakthrough test was 10/90 (v/v).

Table S7. Comparison of C_2H_6 uptake, packing density and Q_{st} of **HOF-ZSTU-5a** with a number of C_2H_6-selective MOFs at room temperature.

**Materials**	**C_2H_6 uptake ($cm^3\ g^{-1}$)**	**C_2H_6 storage density ($kg\ L^{-1}$)**	**C_2H_6 Q_{st} ($kJ\ mol^{-1}$)**
HOF-ZSTU-5a	91.5	0.349	32.9
FJI-H11-Me(des)[38]	57.97	0.30855	38.9
Zn-BPZ-TATB[39]	105.1	0.29325	23.1
TJT-100[40]	83.7	0.28743	29
NPU-1[41]	100.8	0.28723	29.1
Zn-atz-ipa[42]	40.544	0.27015	45.8
CPOC-301[43]	87	0.11312	32.4
JNU-2[44]	91.84	0.21964	29.4
LIFM-63[45]	67.2	0.14516	25.8
$Fe_2(O_2)$(dobdc)[46]	74.3	0.27413	66.8
NKMOF-8-Br[47]	95.01	0.60593	40.8
UPC-66-a[37]	61.7	0.41652	15.61
Tb-MOF-76(NH_2)[48]	73.3	0.41953	32.8
Zn(ad)(int)[49]	51.968	0.24167	33.2
NKMOF-14-PD[50]	119.84	0.21564	19.2
SNNU-40[51]	169	0.18552	18

Table S8. The separation potential (Δq) of **HOF-ZSTU-5a** and several C_2H_6-selective adsorbents were compared at room temperature.

**Materials**	**Δq (mmol g^{-1})**	**Materials**	**Δq (mmol g^{-1})**
CPM-733[52]	1.88	MAF-49[53]	0.78
NKMOF-8-Br	1.8	Azole-Th-1[54]	0.75
$Fe_2(O_2)$(dobdc)	1.74	JNU-2	0.75
Ni-MOF 2[55]	1.7	TJT-100	0.73
MUF-15[56]	1.53	Tb-MOF-76	0.726
TYUT-17[57]	1.51	FJI-H11-Me(des)	0.7
MOF-303[58]	1.3	UPC-66-a[59]	0.64
NKMOF-8-Me	1.3	NPU-1	0.6
MAC-4[60]	1.3	ZJU-HOF-10(sc)	0.6
SNNU-40	1.27	Zn-FBA[61]	0.57
HOF-ZSTU-5a	1.22	LIFM-63	0.54
Zn-BPZ-TATB	1.14	MTHOF-1	0.505
Tb-MOF-76(NH_2)	1.1	CPOC-301	0.48
PCP-IPA[62]	1.1	UPC-613[63]	0.46
Zn(ad)(int)	0.95	NKCOF-23[64]	0.4
NKCOF-21	0.88	ZIF-7[65]	0.4
ZJU-HOF-60a	0.85	NKCOF-22	0.33
$Cu(Qc)_2$[66]	0.85	Zn-atz-oba[67]	0.25
HOF-76a	0.81		

Table S9. Physical properties of C_2H_6 and C_2H_4.[68,69] A molecular model mapping the electrostatic potential shows that the π-electron cloud of C_2H_4 has a more negative electrical character.

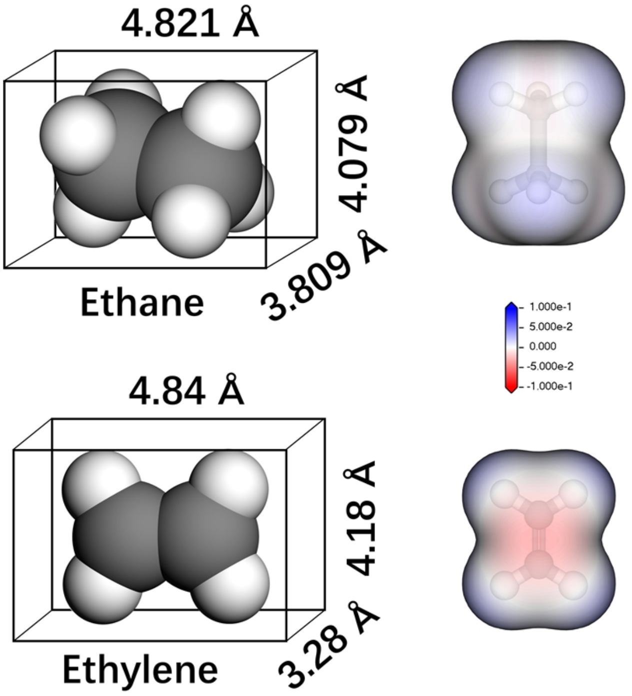

Molecule	Kinetic diameter (Å)	Size	Polarizability ($\times 10^{-25}$ cm^3)	Quadrupole moment ($\times 10^{-26}$ esu cm^2)	Boiling point (K)
C_2H_6	4.443	3.81 × 4.08 × 4.82	44.3−44.7	0.65	184.5
C_2H_4	4.163	3.28 × 4.18 × 4.84	42.52	1.5	169.4

Table S10. Comparison of specific injection amount and C_2H_4 productivity of **HOF-ZSTU-5a** with several C_2H_6-selective MOFs temperature.

**Materials**	**specific injection amount (mmol g^{-1})**	**C_2H_4 productivity (L kg^{-1})**
HOF-ZSTU-5a	2.14286	17.5
JNU-2	1.25	21.2
TYUT-17	3.125	35.2
UPC-66-a	1.68482	18.816
$Fe_2(O_2)$(dobdc)	1.98589	17.7
MOF-303	1.86902	12.544
MAF-49	0.01563	6.3
PCP-IPA	1.4085	26.2
Azole-Th-1	1.35213	15.2
MUF-15	1.07143	14
UiO-67-$(NH_2)_2$[70]	0.68586	12.544
$Cu(Qc)_2$	0.35714	8.71
Zn(ad)(int)	0.66301	7.168
NKCOF-21	1.51786	6.8
MAC-4	0.4375[a]	5.4[a]
Zn-atz-oba	0.13889	2.3744
Ni-MOF 2	0.63776	12
UPC-613	0.89286	10.528

Unless otherwise stated, the data used for the calculation of Specific injection amount and C_2H_4 productivity are those obtained with an equimolar mixture.

[a] The mixture used for the breakthrough test was $C_2H_6/C_2H_4/Ar = 5/5/90$ (v/v/v).

Table S11. A comprehensive comparison of C_2H_6 uptake, binding affinity, selectivity, packing density, separability, stability, and synthesizability of **HOF-ZSTU-5a** and other high-performance carboxylic acid-based HOFs.

Materials	Binding affinity (kJ mol^{-1})	C_2H_6 uptake (cm^3 g^{-1})	Selectivity	Packing density (kg L^{-1})	Synthesizability	Stability	Separability* (mmol g^{-1})
HOF-ZSTU-5a	32.9	91.5	2.0	0.34913	5	5	2.14
HOF-TDCPB	22.9	37.9	1.5	0.22361	3.5	5	0.91518
HOF-NBDA	23.5	89.2	1.75	0.26906	4	4	1.38393
ZJU-HOF-1	31.5	109	2.25	0.2433	3.5	3.5	1.00446
HOF-76a	22.8	66.08	2.0	0.22125	3	4.5	0.40179

*Use of specific injection amount of the gas mixture to represent separability.

Synthesizability and stability were numerically quantified based on literature reports.

HOF-ZSTU-5a

Synthesizability: Single crystal formation in a few hours at room temperature; high solubility of raw materials; low boiling solvent and no solvent exchange required.

Stability: Stable for at least one week in aqueous solutions at pH=1−12; Stable in 12 M HCl for one week; thermally stable to at least 300 °C; superhydrophobic.

HOF-TDCPB

Synthesizability: Single crystal was obtained by diffusion at room temperature for two weeks; DMF and ether are required; MeOH exchange was used to activate the samples; expanded synthesis requires at least one week; requires alkalolysis to recreate new HOFs.

Stability: Stable for one week in a variety of solvents and 12 M HCl; thermally stable to 200 °C; water stable.

HOF-NBDA

Synthesizability: Solvent heat 120 °C for three days; high boiling solvent (DMF and HOAc) and requires solvent exchange (MeOH); enlargeable synthesis.

Stability: Stable for one day in a variety of solvents; stable for one day in 12 M HCl; water stable; heat stable up to 200 °C.

ZJU-HOF-1

Synthesizability: Room temperature diffusion for two weeks; requires large solvent doses (20 mL THF and 100 mL ether).

Stability: Heat stabilized to 200 °C; water stable; stabilized for two days at pH=10 and pH=1.

HOF-76a

Synthesizability: Room temperature diffusion for several weeks with a large solvent volume (15 mL DMSO and 100 mL PA); requires two days of solvent exchange (PA).

Stability: Heat stabilized to 300 °C; water stable; stabilized at pH=10 and pH=1 for one day.

References

1. Yin, Q., Zhao, P., Sa, R.-J., Chen, G.-C., Lü, J., Liu, T.-F., and Cao, R. (2018). An ultra-robust and crystalline redeemable hydrogen-bonded organic framework for synergistic chemo-photodynamic therapy. Angew. Chem. Int. Ed. *57*, 7691-7696. https://doi.org/10.1002/anie.201800354.
2. Ma, K., Li, P., Xin, J.H., Chen, Y., Chen, Z., Goswami, S., Liu, X., Kato, S., Chen, H., Zhang, X., et al. (2020). Ultrastable mesoporous hydrogen-bonded organic framework-based fiber composites toward mustard gas detoxification. Cell Rep. Phys. Sci. *1*, 100024. https://doi.org/10.1016/j.xcrp.2020.100024.
3. Wang, Y., Ma, K., Bai, J., Xu, T., Han, W., Wang, C., Chen, Z., Kirlikovali, K.O., Li, P., Xiao, J., and Farha, O.K. (2022). Chemically engineered porous molecular coatings as reactive oxygen species generators and reservoirs for long-lasting self-cleaning textiles. Angew. Chem. Int. Ed. *61*, e202115956. https://doi.org/10.1002/anie.202115956.
4. Harding, S.D., Liu, T., Chen, L., Yang, S., Borne, I., Fellowes, T., Peters, A.W., Weston, S.C., Ward, J.W., and Cooper, A.I. (2025). Selective CO_2 adsorption in ultrahydrophobic molecular pyrene frameworks by computational design. J. Am. Chem. Soc. *147*, 23160-23169. https://doi.org/10.1021/jacs.5c06861.
5. Yang, W., Wang, J., Wang, H., Bao, Z., Zhao, J.C.-G., and Chen, B. (2017). Highly interpenetrated robust microporous hydrogen-bonded organic framework for gas separation. Cryst. Growth Des. *17*, 6132-6137. https://doi.org/10.1021/acs.cgd.7b01322.
6. Wang, B., Lv, X.-L., Lv, J., Ma, L., Lin, R.-B., Cui, H., Zhang, J., Zhang, Z., Xiang, S., and Chen, B. (2020). A novel mesoporous hydrogen-bonded organic framework with high porosity and stability. Chem. Commun. *56*, 66-69. https://doi.org/10.1039/C9CC07802A.
7. Gao, J., Cai, Y., Qian, X., Liu, P., Wu, H., Zhou, W., Liu, D.-X., Li, L., Lin, R.-B., and Chen, B. (2021). A microporous hydrogen-bonded organic framework for the efficient capture and purification of propylene. Angew. Chem. Int. Ed. *60*, 20400-20406. https://doi.org/10.1002/anie.202106665.
8. Zentner, C.A., Lai, H.W.H., Greenfield, J.T., Wiscons, R.A., Zeller, M., Campana, C.F., Talu, O., FitzGerald, S.A., and Rowsell, J.L.C. (2015). High surface area and Z′ in a thermally stable 8-fold polycatenated hydrogen-bonded framework. Chem. Commun. *51*, 11642-11645. http://doi.org/10.1039/C5CC04219D.
9. Hu, F., Liu, C., Wu, M., Pang, J., Jiang, F., Yuan, D., and Hong, M. (2017). An ultrastable and easily regenerated hydrogen-bonded organic molecular framework with permanent porosity. Angew. Chem. Int. Ed. *56*, 2101-2104. https://doi.org/10.1002/anie.201610901.
10. Cai, Y., Gao, J., Li, J.-H., Liu, P., Zheng, Y., Zhou, W., Wu, H., Li, L., Lin, R.-B., and Chen, B. (2023). Pore modulation of hydrogen-bonded organic frameworks for efficient separation of propylene. Angew. Chem. Int. Ed. *62*, e202308579. https://doi.org/10.1002/anie.202308579.
11. Yu, B., Geng, S., Wang, H., Zhou, W., Zhang, Z., Chen, B., and Jiang, J. (2021). A solid transformation into carboxyl dimers based on a robust hydrogen-bonded organic framework for propyne/propylene separation. Angew. Chem. Int. Ed. *60*, 25942-

25948. https://doi.org/10.1002/anie.202110057.

12. Yang, Y., Li, L., Lin, R.-B., Ye, Y., Yao, Z., Yang, L., Xiang, F., Chen, S., Zhang, Z., Xiang, S., and Chen, B. (2021). Ethylene/ethane separation in a stable hydrogen-bonded organic framework through a gating mechanism. Nat. Chem. *13*, 933-939. https://doi.org/10.1038/s41557-021-00740-z.
13. Delley, B. (2000). From molecules to solids with the $DMol^3$ approach. J. Chem. Phys. *113*, 7756-7764. http://doi.org/10.1063/1.1316015.
14. Delley, B. (1990). An all-electron numerical method for solving the local density functional for polyatomic molecules. J. Chem. Phys. *92*, 508-517. http://doi.org/10.1063/1.458452.
15. Perdew, J.P., Burke, K., and Ernzerhof, M. (1996). Generalized gradient approximation made simple. Phys. Rev. Lett. *77*, 3865-3868. http://doi.org/10.1103/PhysRevLett.77.3865.
16. Clark, S.J., Segall, M.D., Pickard, C.J., Hasnip, P.J., Probert, M.I.J., Refson, K., and Payne, M.C. (2005). First principles methods using CASTEP. Z Krist-Cryst Mater. *220*, 567-570. http://doi.org/10.1524/zkri.220.5.567.65075.
17. Dolomanov, O.V., Bourhis, L.J., Gildea, R.J., Howard, J.A.K., and Puschmann, H. (2009). *OLEX2*: a complete structure solution, refinement and analysis program. J. Appl. Crystallogr. *42*, 339-341. http://doi.org/10.1107/S0021889808042726.
18. Meng, X., Zou, S., Li, J., Chen, C., Zhang, J., and Wu, M. (2025). A noncovalent π-stacked dual-pore molecular crystal for ethanol/water and benzene/methanol azeotrope separation. Angew. Chem. Int. Ed. *64*, e202415346. https://doi.org/10.1002/anie.202415346.
19. Li, H., Chen, C., Li, Q., Kong, X.J., Liu, Y., Ji, Z., Zou, S., Hong, M., and Wu, M. (2024). An ultra-stable supramolecular framework based on consecutive side-by-side hydrogen bonds for one-step C_2H_4/C_2H_6 separation. Angew. Chem. Int. Ed. *63*, e202401754. https://doi.org/10.1002/anie.202401754.
20. Zhang, X.-W., He, H., Gan, Y.-W., Wang, Y., Huang, N.-Y., Liao, P.-Q., Zhang, J.-P., and Chen, X.-M. (2024). High-pressure molecular sieving of high-humidity C_2H_4/C_2H_6 mixture by a hydrophobic flexible metal–organic framework. Angew. Chem. Int. Ed. *63*, e202317648. https://doi.org/10.1002/anie.202317648.
21. Wang, Y., Li, T., Li, L., Lin, R.-B., Jia, X., Chang, Z., Wen, H.-M., Chen, X.-M., and Li, J. (2023). Construction of fluorinated propane-trap in metal–organic frameworks for record polymer-grade propylene production under high humidity conditions. Adv. Mater. *35*, 2207955. https://doi.org/10.1002/adma.202207955.
22. Zhou, D.-D., Chen, P., Wang, C., Wang, S.-S., Du, Y., Yan, H., Ye, Z.-M., He, C.-T., Huang, R.-K., Mo, Z.-W., et al. (2019). Intermediate-sized molecular sieving of styrene from larger and smaller analogues. Nat. Mater. *18*, 994-998. http://doi.org/10.1038/s41563-019-0427-z.
23. Chen, F., Prasetyo, N., Sakaki, S., Otake, K.-i., and Kitagawa, S. (2025). Benchmark paraffin adsorption in a super-hydrophobic porous coordination polymer with blade-like circular phenyl nanotraps. Angew. Chem. Int. Ed. *64*, e202423371. https://doi.org/10.1002/anie.202423371.
24. Ye, Z.-M., Zhang, X.-W., Liao, P.-Q., Xie, Y., Xu, Y.-T., Zhang, X.-F., Wang, C., Liu, D.-X., Huang, N.-Y., Qiu, Z.-H., et al. (2020). A hydrogen-bonded yet hydrophobic porous molecular crystal for molecular-sieving-like separation of butane and isobutane. Angew. Chem. Int. Ed. *59*, 23322-23328. https://doi.org/10.1002/anie.202011300.
25. Li, L., Zhang, X., Lian, X., Zhang, L., Zhang, Z., Liu, X., He, T., Li, B., Chen, B., and Bu, X.-H. (2025). Flue gas desulfurization and SO_2 recovery within a flexible hydrogen-bonded organic framework. Nat. Chem. *17*, 727-733. https://doi.org/10.1038/s41557-025-01744-9.
26. Yi, M., Wang, S., Li, S., Zhang, S., Liu, Y., Zhang, L., You, Z., Liu, X., Li, L., Wang, J., et al. (2025). Superhydrophobic molecular selector for efficient separation of ethane over ethylene under dry and humid conditions. J. Am. Chem. Soc. *147*, 13592-13600. https://doi.org/10.1021/jacs.5c00658.
27. Wang, G.-D., Li, Y.-Z., Shi, W.-J., Hou, L., Wang, Y.-Y., and Zhu, Z. (2022). One-step C_2H_4 purification from ternary $C_2H_6/C_2H_4/C_2H_2$ mixtures by a robust metal–organic framework with customized pore environment. Angew. Chem. Int. Ed. *61*, e202205427. https://doi.org/10.1002/anie.202205427.
28. Zhou, Y., Chen, C., Krishna, R., Ji, Z., Yuan, D., and Wu, M. (2023). Tuning pore polarization to boost ethane/ethylene

separation performance in hydrogen-bonded organic frameworks. Angew. Chem. Int. Ed. *62*, e202305041. https://doi.org/10.1002/anie.202305041.

29. Zhang, L., Lei, Q., Yi, M., Zhang, Z., Lian, X., Xu, J., Zhang, S., Li, L., Li, B., and Bu, X.-H. (2025). Bioinspired "intermolecular pocket" in soft molecular crystal of porous organic cage exhibiting reversible guest recognition. Angew. Chem. Int. Ed. *64*, e202421753. https://doi.org/10.1002/anie.202421753.
30. Wang, Y.-B., Zhang, T.-F., Lin, Y.-X., Wang, J.-X., Wen, H.-M., Zhang, X., Qian, G., and Li, B. (2024). A novel microporous hydrogen-bonded organic framework with electron-rich alkynyl groups for highly efficient C_2H_6/C_2H_4 separation. J. Mater. Chem. A *12*, 25812-25819. http://doi.org/10.1039/D4TA04528A.
31. Zhang, X., Wang, J.-X., Li, L., Pei, J., Krishna, R., Wu, H., Zhou, W., Qian, G., Chen, B., and Li, B. (2021). A rod-packing hydrogen-bonded organic framework with suitable pore confinement for benchmark ethane/ethylene separation. Angew. Chem. Int. Ed. *60*, 10304-10310. https://doi.org/10.1002/anie.202100342.
32. Ji, Z., Li, Q., Zhou, Y., Krishna, R., Hong, M., and Wu, M. (2024). Synergistic C_2H_2 binding sites in hydrogen-bonded supramolecular framework for one-step C_2H_4 purification from ternary C2 mixture. Angew. Chem. Int. Ed. *63*, e202411175. https://doi.org/10.1002/anie.202411175.
33. Wang, J.-X., Gu, X.-W., Lin, Y.-X., Li, B., and Qian, G. (2021). A novel hydrogen-bonded organic framework with highly permanent porosity for boosting ethane/ethylene separation. ACS Mater. Lett. *3*, 497-503. http://doi.org/10.1021/acsmaterialslett.1c00013.
34. Chen, Y., Zhao, Y., Zhao, Y., Chen, X., Liu, X., Li, L., Cao, D., Wang, S., and Zhang, L. (2024). A novel homoconjugated propellane triimide: Synthesis, structural analyses, and gas separation. Angew. Chem. Int. Ed. *63*, e202401706. https://doi.org/10.1002/anie.202401706.
35. Zhang, X., Li, L., Wang, J.-X., Wen, H.-M., Krishna, R., Wu, H., Zhou, W., Chen, Z.-N., Li, B., Qian, G., and Chen, B. (2020). Selective ethane/ethylene separation in a robust microporous hydrogen-bonded organic framework. J. Am. Chem. Soc. *142*, 633-640. https://doi.org/10.1021/jacs.9b12428.
36. Guo, F.-A., Zhou, K., Liu, J., Wang, H., and Li, J. (2023). Robust hydrogen-bonded organic framework with four-fold interpenetration for adsorptive separation of C_2H_6/C_2H_4 and Xe/Kr. Precis. Chem. *1*, 524-529. http://doi.org/10.1021/prechem.3c00040.
37. Liu, J., Miao, J., Ullah, S., Zhou, K., Yu, L., Wang, H., Wang, Y., Thonhauser, T., and Li, J. (2022). A water-resistant hydrogen-bonded organic framework for ethane/ethylene separation in humid environments. ACS Mater. Lett. *4*, 1227-1232. http://doi.org/10.1021/acsmaterialslett.2c00370.
38. Di, Z., Liu, C., Pang, J., Zou, S., Ji, Z., Hu, F., Chen, C., Yuan, D., Hong, M., and Wu, M. (2022). A metal-organic framework with nonpolar pore surfaces for the one-step acquisition of C_2H_4 from a C_2H_4 and C_2H_6 mixture. Angew. Chem. Int. Ed. *61*, e202210343. https://doi.org/10.1002/anie.202210343.
39. Wang, G.-D., Li, Y.-Z., Shi, W.-J., Hou, L., Wang, Y.-Y., and Zhu, Z. (2023). Active sites decorated nonpolar pore-based MOF for one-step acquisition of C_2H_4 and recovery of C_3H_6. Angew. Chem. Int. Ed. *62*, e202311654. https://doi.org/10.1002/anie.202311654.
40. Hao, H.-G., Zhao, Y.-F., Chen, D.-M., Yu, J.-M., Tan, K., Ma, S., Chabal, Y., Zhang, Z.-M., Dou, J.-M., Xiao, Z.-H., et al. (2018). Simultaneous trapping of C_2H_2 and C_2H_6 from a ternary mixture of C_2H_2/C_2H_4/C_2H_6 in a robust metal–organic framework for the purification of C_2H_4. Angew. Chem. Int. Ed. *57*, 16067-16071. https://doi.org/10.1002/anie.201809884.
41. Zhu, B., Cao, J.-W., Mukherjee, S., Pham, T., Zhang, T., Wang, T., Jiang, X., Forrest, K.A., Zaworotko, M.J., and Chen, K.-J. (2021). Pore engineering for one-step ethylene purification from a three-component hydrocarbon mixture. J. Am. Chem. Soc. *143*, 1485-1492. http://doi.org/10.1021/jacs.0c11247.
42. Chen, K.-J., Madden, D.G., Mukherjee, S., Pham, T., Forrest, K.A., Kumar, A., Space, B., Kong, J., Zhang, Q.-Y., and Zaworotko, M.J. (2019). Synergistic sorbent separation for one-step ethylene purification from a four-component mixture. Science *366*, 241-246. https://doi.org/10.1126/science.aax8666.
43. Su, K., Wang, W., Du, S., Ji, C., and Yuan, D. (2021). Efficient ethylene purification by a robust ethane-trapping porous

organic cage. Nat. Commun. *12*, 3703. http://doi.org/10.1038/s41467-021-24042-7.

44. Zeng, H., Xie, X.-J., Xie, M., Huang, Y.-L., Luo, D., Wang, T., Zhao, Y., Lu, W., and Li, D. (2019). Cage-interconnected metal–organic framework with tailored apertures for efficient C_2H_6/C_2H_4 separation under humid conditions. J. Am. Chem. Soc. *141*, 20390-20396. http://doi.org/10.1021/jacs.9b10923.
45. Chen, C.-X., Wei, Z.-W., Pham, T., Lan, P.C., Zhang, L., Forrest, K.A., Chen, S., Al-Enizi, A.M., Nafady, A., Su, C.-Y., and Ma, S. (2021). Nanospace engineering of metal–organic frameworks through dynamic spacer installation of multifunctionalities for efficient separation of ethane from ethane/ethylene mixtures. Angew. Chem. Int. Ed. *60*, 9680-9685. https://doi.org/10.1002/anie.202100114.
46. Li, L., Lin, R.-B., Krishna, R., Li, H., Xiang, S., Wu, H., Li, J., Zhou, W., and Chen, B. (2018). Ethane/ethylene separation in a metal-organic framework with iron-peroxo sites. Science *362*, 443-446. https://doi.org/10.1126/science.aat0586.
47. Geng, S., Lin, E., Li, X., Liu, W., Wang, T., Wang, Z., Sensharma, D., Darwish, S., Andaloussi, Y.H., Pham, T., et al. (2021). Scalable room-temperature synthesis of highly robust ethane-selective metal–organic frameworks for efficient ethylene purification. J. Am. Chem. Soc. *143*, 8654-8660. http://doi.org/10.1021/jacs.1c02108.
48. Wang, G.-D., Krishna, R., Li, Y.-Z., Shi, W.-J., Hou, L., Wang, Y.-Y., and Zhu, Z. (2022). Boosting ethane/ethylene separation by MOFs through the amino-functionalization of pores. Angew. Chem. Int. Ed. *61*, e202213015. https://doi.org/10.1002/anie.202213015.
49. Ding, Q., Zhang, Z., Liu, Y., Chai, K., Krishna, R., and Zhang, S. (2022). One-step ethylene purification from ternary mixtures in a metal–organic framework with customized pore chemistry and shape. Angew. Chem. Int. Ed. *61*, e202208134. https://doi.org/10.1002/anie.202208134.
50. Liu, W., Geng, S., Li, N., Wang, S., Jia, S., Jin, F., Wang, T., Forrest, K.A., Pham, T., Cheng, P., et al. (2023). Highly robust microporous metal-organic frameworks for efficient ethylene purification under dry and humid conditions. Angew. Chem. Int. Ed. *62*, e202217662. https://doi.org/10.1002/anie.202217662.
51. Li, Y.-P., Zhao, Y.-N., Li, S.-N., Yuan, D.-Q., Jiang, Y.-C., Bu, X., Hu, M.-C., and Zhai, Q.-G. (2021). Ultrahigh-uptake capacity-enabled gas separation and fruit preservation by a new single-walled nickel–organic framework. Adv. Sci. *8*, 2003141. https://doi.org/10.1002/advs.202003141.
52. Yang, H., Wang, Y., Krishna, R., Jia, X., Wang, Y., Hong, A.N., Dang, C., Castillo, H.E., Bu, X., and Feng, P. (2020). Pore-space-partition-enabled exceptional ethane uptake and ethane-selective ethane–ethylene separation. J. Am. Chem. Soc. *142*, 2222-2227. http://doi.org/10.1021/jacs.9b12924.
53. Liao, P.-Q., Zhang, W.-X., Zhang, J.-P., and Chen, X.-M. (2015). Efficient purification of ethene by an ethane-trapping metal-organic framework. Nat. Commun. *6*, 8697. https://doi.org/10.1038/ncomms9697.
54. Xu, Z., Xiong, X., Xiong, J., Krishna, R., Li, L., Fan, Y., Luo, F., and Chen, B. (2020). A robust th-azole framework for highly efficient purification of C_2H_4 from a $C_2H_4/C_2H_2/C_2H_6$ mixture. Nat. Commun. *11*, 3163. http://doi.org/10.1038/s41467-020-16960-9.
55. Ye, Y., Xie, Y., Shi, Y., Gong, L., Phipps, J., Al-Enizi, A.M., Nafady, A., Chen, B., and Ma, S. (2023). A microporous metal-organic framework with unique aromatic pore surfaces for high performance C_2H_6/C_2H_4 separation. Angew. Chem. Int. Ed. *62*, e202302564. https://doi.org/10.1002/anie.202302564.
56. Qazvini, O.T., Babarao, R., Shi, Z.-L., Zhang, Y.-B., and Telfer, S.G. (2019). A robust ethane-trapping metal–organic framework with a high capacity for ethylene purification. J. Am. Chem. Soc. *141*, 5014-5020. http://doi.org/10.1021/jacs.9b00913.
57. Zhang, L., Yu, B., Wang, M., Chen, Y., Wang, Y., Sun, L.-B., Zhang, Y.-B., Zhang, Z., Li, J., and Li, L. (2025). Ethane triggered gate-opening in a flexible-robust metal-organic framework for ultra-high purity ethylene purification. Angew. Chem. Int. Ed. *64*, e202418853. https://doi.org/10.1002/anie.202418853.
58. Wen, H.-M., Yu, C., Liu, M., Lin, C., Zhao, B., Wu, H., Zhou, W., Chen, B., and Hu, J. (2023). Construction of negative electrostatic pore environments in a scalable, stable and low-cost metal-organic framework for one-step ethylene purification from ternary mixtures. Angew. Chem. Int. Ed. *62*, e202309108. https://doi.org/10.1002/anie.202309108.

59. Wang, Y., Fu, M., Zhou, S., Liu, H., Wang, X., Fan, W., Liu, Z., Wang, Z., Li, D., Hao, H., et al. (2022). Guest-molecule-induced self-adaptive pore engineering facilitates purification of ethylene from ternary mixture. Chem *8*, 3263-3274. https://doi.org/10.1016/j.chempr.2022.08.014.
60. Wang, G.-D., Li, Y.-Z., Krishna, R., Zhang, W.-Y., Hou, L., Wang, Y.-Y., and Zhu, Z. (2024). Scalable synthesis of robust MOF for challenging ethylene purification and propylene recovery with record productivity. Angew. Chem. Int. Ed. *63*, e202319978. https://doi.org/10.1002/anie.202319978.
61. Yang, L., Yan, L., Niu, W., Feng, Y., Fu, Q., Zhang, S., Zhang, Y., Li, L., Gu, X., Dai, P., et al. (2022). Adsorption in reversed order of C_2 hydrocarbons on an ultramicroporous fluorinated metal-organic framework. Angew. Chem. Int. Ed. *61*, e202204046. https://doi.org/10.1002/anie.202204046.
62. Zhang, P., Yang, L., Liu, X., Wang, J., Suo, X., Chen, L., Cui, X., and Xing, H. (2022). Ultramicroporous material based parallel and extended paraffin nano-trap for benchmark olefin purification. Nat. Commun. *13*, 4928. https://doi.org/10.1038/s41467-022-32677-3.
63. Wang, Y., Hao, C., Fan, W., Fu, M., Wang, X., Wang, Z., Zhu, L., Li, Y., Lu, X., Dai, F., et al. (2021). One-step ethylene purification from an acetylene/ethylene/ethane ternary mixture by cyclopentadiene cobalt-functionalized metal–organic frameworks. Angew. Chem. Int. Ed. *60*, 11350-11358. https://doi.org/10.1002/anie.202100782.
64. Jin, F., Lin, E., Wang, T., Geng, S., Wang, T., Liu, W., Xiong, F., Wang, Z., Chen, Y., Cheng, P., and Zhang, Z. (2022). Bottom-up synthesis of 8-connected three-dimensional covalent organic frameworks for highly efficient ethylene/ethane separation. J. Am. Chem. Soc. *144*, 5643-5652. http://doi.org/10.1021/jacs.2c01058.
65. Gücüyener, C., van den Bergh, J., Gascon, J., and Kapteijn, F. (2010). Ethane/ethene separation turned on its head: selective ethane adsorption on the metal−organic framework ZIF-7 through a gate-opening mechanism. J. Am. Chem. Soc. *132*, 17704-17706. http://doi.org/10.1021/ja1089765.
66. Lin, R.-B., Wu, H., Li, L., Tang, X.-L., Li, Z., Gao, J., Cui, H., Zhou, W., and Chen, B. (2018). Boosting ethane/ethylene separation within isoreticular ultramicroporous metal–organic frameworks. J. Am. Chem. Soc. *140*, 12940-12946. https://doi.org/10.1021/jacs.8b07563.
67. Cao, J.-W., Mukherjee, S., Pham, T., Wang, Y., Wang, T., Zhang, T., Jiang, X., Tang, H.-J., Forrest, K.A., Space, B., et al. (2021). One-step ethylene production from a four-component gas mixture by a single physisorbent. Nat. Commun. *12*, 6507. http://doi.org/10.1038/s41467-021-26473-8.
68. Li, J.-R., Kuppler, R.J., and Zhou, H.-C. (2009). Selective gas adsorption and separation in metal–organic frameworks. Chem. Soc. Rev. *38*, 1477-1504. https://doi.org/10.1039/B802426J.
69. Wang, H., Liu, Y., and Li, J. (2020). Designer metal–organic frameworks for size-exclusion-based hydrocarbon separations: progress and challenges. Adv. Mater. *32*, 2002603. https://doi.org/10.1002/adma.202002603.
70. Gu, X.-W., Wang, J.-X., Wu, E., Wu, H., Zhou, W., Qian, G., Chen, B., and Li, B. (2022). Immobilization of lewis basic sites into a stable ethane-selective MOF enabling one-step separation of ethylene from a ternary mixture. J. Am. Chem. Soc. *144*, 2614-2623. http://doi.org/10.1021/jacs.1c10973.
